# In Silico Psycho-Oncology: Understanding Resilience Pathways in Breast Cancer—Determinants of Longitudinal Depression and Quality-of-Life Trajectories

**DOI:** 10.3390/jpm16040209

**Published:** 2026-04-07

**Authors:** Eleni Kolokotroni, Paula Poikonen-Saksela, Ruth Pat-Horenczyk, Berta Sousa, Albino J. Oliveira-Maia, Ketti Mazzocco, Haridimos Kondylakis, Georgios S. Stamatakos

**Affiliations:** 1 In Silico Oncology and In Silico Medicine Group, Institute of Communication and Computer Systems, School of Electrical and Computer Engineering, National Technical University of Athens, Iroon Polytechniou 9, 15780 Zografos, Greece; 2 Department of Oncology, Faculty of Medicine, Helsinki University Hospital, University of Helsinki, 00029 HUS Helsinki, Finland; paula.poikonen-saksela@hus.fi; 3 School of Social Work and Social Welfare, Hebrew University of Jerusalem, Jerusalem 91905, Israel; ruth.pat-horenczyk@mail.huji.ac.il; 4 Breast Unit, Champalimaud Clinical Centre, Champalimaud Foundation, 1400-038 Lisbon, Portugal; berta.sousa@fundacaochampalimaud.pt; 5 Champalimaud Research & Clinical Centre, Champalimaud Foundation, 1400-038 Lisbon, Portugal; albino.maia@neuro.fchampalimaud.org; 6 Istituto Europeo di Oncologia, 20141 Milano, Italy; ketti.mazzocco@ieo.it; 7 Foundation for Research and Technology–Hellas, 70013 Heraklion, Greece; kondylak@ics.forth.gr

**Keywords:** in silico medicine, in silico psycho-oncology, machine learning, artificial intelligence, breast cancer, resilience, depression, quality of life

## Abstract

**Background/Objectives:** Patients with breast cancer show substantial heterogeneity in terms of psychological adjustment following diagnosis. We aimed to characterize longitudinal trajectories of quality of life (QoL) and depressive symptoms during the first 18 months post-diagnosis and to identify robust clinical, psychosocial, and behavioral predictors associated with distinct adjustment pathways. **Methods:** Women (*N* = 538; mean age 55.4 years; range 40–70) with operable breast cancer (stages I–III) were drawn from the multicenter BOUNCE cohort. QoL (Global Health Status/QoL scale of the European Organisation for Research and Treatment of Cancer Quality of Life Questionnaire Core 30) and depressive symptoms (depression subscale of the Hospital Anxiety and Depression Scale) were assessed at baseline and months 3, 6, 9, 12, 15 and 18. Latent class growth analysis and growth mixture modeling identified distinct trajectory classes. Associations between early predictors and trajectory membership were examined using logistic regression combined with elastic net regularization. **Results:** Depression trajectories demonstrated heterogeneity, with groups characterized by persistent resilience (59.7%), stable moderate/high (25.3%), delayed onset (5.0%), and recovery (10.0%). QoL trajectories ranged from stable excellent (13.2%) and stable high (40.7%) to moderate (31.4%) and persistent low/deteriorating (6.9%), as well as a distinct recovering trajectory (7.8%). Trajectory differentiation was primarily driven by psychological resources, symptom burden, functional status, and coping processes, alongside specific contributions from clinical factors. **Conclusions:** Distinct subgroups of women with breast cancer follow divergent adjustment pathways. These findings highlight the multidimensional nature of resilience and support the need for tailored interventions that promote long-term well-being beyond simple risk reduction.

## 1. Introduction

Patient resilience is a critical determinant of cancer treatment outcomes, influencing both psychological adaptation and overall quality of life (QoL) [1]. Resilience does not imply the absence of distress; rather, it reflects a dynamic process of adaptation through which individuals mobilize internal and external resources to maintain or restore well-being when facing adversity such as a cancer diagnosis [2,3]. Resilience can be understood from three complementary perspectives: as a capacity, as an adaptive process, and as an outcome [2,4]. As a capacity, resilience involves the integration of psychological and social resources—such as optimism, self-efficacy, cognitive flexibility, acceptance, and social support—that shape how individuals cope with adversity. As a process, resilience reflects adaptive adjustment over time, where individuals may experience temporary disruptions in functioning but gradually return to stable psychological and behavioral functioning. Finally, resilience can be viewed as an outcome, reflected in the maintenance of subjective well-being and satisfactory QoL despite exposure to significant stressors.

Consistent with this framework, higher levels of resilience have been associated with internal psychological resources such as optimism, self-efficacy and adaptive emotion regulation strategies and external resources such as social support, as well as with younger age, female sex, higher socioeconomic status and being married [5,6,7]. Positive cognitive strategies, including acceptance and positive thinking, are linked to enhanced psychological well-being, whereas maladaptive strategies such as rumination and catastrophizing are consistently associated with adverse emotional outcomes [8,9,10,11,12,13,14]. Among women with breast cancer, optimism and self-efficacy are predictors of better psychological well-being, improved QoL and lower distress over time [15,16], while perceived social and family support facilitates adaptive coping and resilience [17,18,19,20].

While these psychosocial factors are known correlates of well-being, most studies rely on static, cross-sectional data that fails to capture the heterogeneous pathways of psychological adaptation over time. Divergent pathways typically range from stable resilience (maintaining high well-being throughout) and recovery (initial distress followed by a return to the baseline level), to chronic impairment or delayed deterioration [2,21,22]. In the present study, resilience is conceptualized as being reflected in longitudinal patterns of adjustment, such as the maintenance of high QoL or the absence of persistent depressive symptoms over time [21,23]. Identifying the factors associated with favorable versus maladaptive adjustment patterns is essential for improving the prediction of psychological outcomes and informing targeted supportive interventions.

Within this context, in the framework of the European Commission (EU)-funded BOUNCE project (https://www.bounce-project.eu/; accessed on 20 December 2025), a prospective multicenter clinical study was conducted across Finland, Italy, Portugal and Israel in order to investigate predictors of resilience trajectories among women with breast cancer. The study collected information about a thorough range of sociodemographic, clinical, psychological and functional factors with the aim of improving the ability to predict resilience in response to breast cancer and, ultimately, to inform personalized interventions that promote effective psychological recovery.

The aim of the current study is twofold: (1) to identify distinct longitudinal trajectories of depression and QoL over the 18 months following diagnosis and/or surgery and (2) to identify factors associated with these trajectories, with particular focus on patterns reflecting resilience, recovery, deterioration and persistent impairment. In order to address the first aim, we applied latent class growth analysis (LCGA) and growth mixture modeling (GMM), which are trajectory-based methods widely used in psycho-oncology that allow the identification of latent subgroups of individuals who follow distinct longitudinal patterns rather than assuming a single average trajectory for the entire population. Unlike approaches that classify patients based on simple change scores between the baseline and follow-up assessments, these methods use all available repeated measurements to capture heterogeneous patterns of psychological adaptation over time. To address the second aim, we used logistic regression models to examine associations between candidate predictors and trajectory membership, allowing results to remain clinically interpretable. In addition to univariate analyses, we performed multivariable variable selection using elastic net regularization. Variable selection is used to identify parsimonious sets of predictors that jointly differentiate trajectory groups, while accounting for correlations between variables and limiting overfitting. This approach moves beyond isolated univariate associations and supports a clearer interpretation of factors relevant for personalized risk assessment and intervention.

To capture clinically meaningful heterogeneity in longitudinal mental health and quality-of-life (QoL) outcomes, we focused on selected comparisons between trajectory groups that address complementary research questions. Specifically, we examined:

- Who remains resilient over time, compared with individuals who experience persistently elevated depressive symptoms or persistently lower QoL, thereby capturing differences in long-term outcome levels.
- Who is at risk for persistently poor depression or QoL outcomes, providing insight into profiles associated with sustained vulnerability.
- Who deteriorates despite early resilience; a contrast that is less confounded by baseline outcome levels and enables the identification of early warning markers relevant to preventive strategies, clinical monitoring and early intervention.
- Who recovers among individuals with comparable baseline levels; a comparison that is likewise less influenced by baseline outcome levels and highlights factors associated with improvement rather than symptom burden, with potential implications for therapeutic intervention.

This study contributes to the psycho-oncology literature by drawing on the multinational BOUNCE cohort to examine heterogeneous trajectories of psychological adaptation following breast cancer diagnosis. Using a computational (“in silico”) data-driven analytical framework, including trajectory clustering (LCGA/GMM) and penalized regression-based variable selection, applied to a comprehensive set of sociodemographic, clinical, and psychological factors, the study focuses on resilience-oriented contrasts to identify the most relevant predictors of favorable and maladaptive adjustment patterns over time.

## 2. Materials and Methods

### 2.1. Participants

The BOUNCE project (“Predicting Effective Adaptation to Breast Cancer to Help Women to BOUNCE Back”) was a multicenter prospective study designed to identify factors associated with psychological adaptation following breast cancer diagnosis and treatment (https://www.bounce-project.eu/; accessed on 20 December 2025). Data were collected between January 2019 and April 2022. Women diagnosed with breast cancer were recruited from four clinical sites across Europe: Helsinki University Hospital (Finland), Shaare Zedek Medical Center and Rabin Medical Center coordinated by the Hebrew University of Jerusalem (Israel), European Institute of Oncology (Italy) and Champalimaud Clinical Centre (Portugal). For simplicity, clinical sites are referred to by country throughout the manuscript. Participants were enrolled approximately three to four weeks after diagnosis.

Eligible participants were female patients aged 40–70 years at diagnosis with histologically confirmed operable invasive breast cancer (tumor stage I–III), who were receiving surgery as part of local treatment and any form of systemic therapy for breast cancer and who provided written informed consent signed by both the patient and the treating physician. This specific age range was selected in order to capture the age groups in which breast cancer incidence is most common [24], while minimizing potential confounding related to age-associated comorbidities (prevalent in patients > 70 years) and the distinct psychosocial life-stage factors, such as fertility concerns and early-career development, typically observed in patients < 40 years. Exclusion criteria included refusal to consent; presence of metastatic disease or a history of another malignancy within the previous five years; a history of serious medical conditions; major surgery within the four weeks preceding enrollment; ongoing treatment for another invasive cancer or major illness; and pregnancy or breastfeeding at the time of recruitment. Furthermore, psychiatric or neurological conditions with onset before age 40 were excluded in order to minimize the influence of long-standing pre-existing mental health trajectories and to ensure a stable baseline for modeling psychological adjustment following breast cancer diagnosis. Ethical approval was obtained from the Ethics Committee of the European Institute of Oncology (Approval No. R868/18-IEO916) and from the corresponding ethics committees of all participating centers, while all participants provided written informed consent prior to inclusion.

Data were collected at seven assessment points at three-month intervals, from baseline (M0) assessment to 18 months of follow-up (M18). Psychological symptoms and subjective health status were assessed at all time points, whereas sociodemographic, lifestyle and medical or disease-related variables considered in this analysis were collected at baseline assessment, with the exception of treatment data, which were collected at month 6 (M6) or month 12 (M12) assessment (study protocol at [25]). Of the 790 participants with records in the BOUNCE dataset, 57 (7.2%) were excluded based on eligibility criteria and 195 (24.7%) due to insufficient data completeness. The final analytic sample consisted of 538 patients with baseline psychological assessments, at least one long-term follow-up assessment (month 12, 15, or 18) and no more than three missing assessments during the 18-month follow-up period.

To assess potential selection bias, baseline characteristics of included participants (*n* = 538) and excluded patients due to insufficient data completeness (*n* = 195) were compared. Categorical variables were analyzed using χ^2^ tests stratified by clinical site, while continuous variables were compared using analysis of variance (ANOVA) adjusted for clinical site. No significant differences were observed in baseline levels of depression, sociodemographic characteristics, cancer-related clinical factors or treatment types (all *p* > 0.05). Minor differences were observed for baseline quality of life (*p* = 0.008) and menopausal status prior to therapy in one clinical site (*p* = 0.01). Overall, the clinical profiles of included and excluded participants were largely comparable, suggesting limited evidence of systematic baseline differences between the groups.

### 2.2. Measures

#### 2.2.1. Outcome Variables

Two outcome variables are considered, reflecting psychological distress and health-related QoL, and these are assessed at seven time points during an 18-month follow-up period.

Psychological distress was measured using the depression subscale of the Hospital Anxiety and Depression Scale [26] (HADS). It derives from seven items on the HADS questionnaire, with higher scores indicating greater severity of depressive symptoms. Following the standardized data structure of the multicenter BOUNCE project, scores are reported as the mean item score (range 0–3), corresponding to the traditional summed score ranging from 0 to 21. HADS depression score was interpreted using established thresholds, with scores of 0–1 indicating low depressive symptoms (corresponding to 0–7 on the 0–21 scale), 1.14–1.42 moderate symptoms (8–10 on the 0–21 scale) and ≥1.57 high symptomatology (≥11 on the 0–21 scale) [27]. Longitudinal changes were interpreted based on published minimally important difference criteria, whereby changes of approximately 0.2–0.43 points were considered clinically meaningful and changes of ≥0.43 points indicative of moderate to large clinical change [28,29,30]. In this study, the internal consistency of the depression scale was high, with Cronbach’s alpha (α) ranging from 0.83 to 0.86 across time points.

Overall health-related QoL was assessed using the Global Health Status/Quality of Life [31] (GHS/QoL) scale of the European Organisation for Research and Treatment of Cancer Quality of Life Questionnaire C30 (EORTC QLQ-C30). This scale reflects patients’ self-reported overall health and QoL and is derived from two items on the EORTC QLQ-C30, with higher scores (range 0–100) indicating better well-being. As no official clinical cut-offs are defined, interpretation of absolute GHS/QoL levels was guided by published empirical thresholds, with scores ≥ 70 considered indicative of good to excellent QoL, scores between approximately 50 and 69 reflecting moderate QoL and scores < 50 indicating poor QoL [32,33]. Longitudinal changes in GHS/QoL were interpreted using established minimally important difference criteria, with changes of 5–10 points considered small but clinically meaningful and changes ≥10 points considered moderate to large [34]. Internal consistency for the GHS/QoL scale remained stable and high across time points (α = 0.85–0.92).

#### 2.2.2. Sociodemographic, Lifestyle and Clinical Data

The analysis incorporated socioeconomic and lifestyle factors, health-related background variables and tumor characteristics collected at baseline assessment. Treatment data were collected at the month 6 (M6) or month 12 (M12) assessment, when treatments were either completed or ongoing.

Socioeconomic and lifestyle data collected at baseline assessment included age, body mass index (BMI), clinical site (Portugal; Italy; Finland; or Israel), educational attainment (non-university or university), marital status (single/engaged; married/common-law; or divorced/widowed), employment status (employed full-/part-time or self-employed; unemployed/housewife; or retired) and monthly household income (low; middle; or high). To account for cross-country differences in income levels and cost of living, country-specific thresholds were used to approximate comparable income categories across clinical sites. Countries were grouped into relatively higher-income settings (Finland and Israel) and comparatively lower-income settings (Portugal and Italy) based on international economic indicators (World Bank, 2023) [35]. Accordingly, low monthly income was defined as ≤1000 EUR in Portugal and Italy and ≤1500 EUR in Finland and Israel, whereas high monthly income was defined as >3000 EUR and >3500 EUR, respectively.

Lifestyle factors included physical activity level (none; low/moderate; or heavy), dietary pattern (no specific diet; mediterranean/vegetarian-type diet; or special diet), alcohol consumption (no consumption; moderate consumption; or heavy consumption) and smoking status (current smoker; never a smoker; or former smoker). Heavy alcohol consumption was defined as intake of more than three drinks on any day or more than seven drinks per week. Physical activity was categorized using an adaptation of the WHO Guidelines on Physical Activity and Sedentary Behaviour [36]. Heavy physical activity was defined as ≥200 min per week of moderate aerobic activity; ≥100 min per week of vigorous aerobic activity; ≥5 weekly strength-training sessions; or combined aerobic and strength activity meeting ≥100–180 min per week of moderate (or ≥50–90 min per week of vigorous) aerobic exercise with ≥1–4 weekly strength sessions.

Health history variables included presence of chronic diseases (no/yes), metabolic diseases (no/yes), mental illness (no/yes), exposure to negative life events (none; one event; or two or more events) and family history of breast cancer (no/yes). These variables were considered background health factors potentially affecting treatment tolerance and psychological outcomes.

Clinical and cancer-related data included menopausal status prior to cancer diagnosis (pre-/perimenopausal or postmenopausal), use of hormone replacement therapy before diagnosis (no/yes), tumor stage (I, II, or III), tumor grade (I, II, or III) and histological subtype (ductal; lobular; or other). Tumor biomarker characteristics comprised estrogen receptor (ER) status (negative/positive), progesterone receptor (PR) status (negative/positive), human epidermal growth factor receptor 2 (HER2) status (negative/positive) and Ki67 proliferation index (<20% or ≥20%). Molecular subtypes were defined as luminal A-like (ER+, PR+, HER2−, or low Ki67 < 20%); luminal B-like (HER2−: ER+, PR+/− with high Ki67 ≥ 20% or ER+, PR− with any Ki67; or HER2+: ER+, PR+/− with any Ki67); HER2-positive non-luminal (ER−, PR−, or HER2+); and triple-negative (ER−, PR−, or HER2−). Treatment-related variables included type of surgery (lumpectomy or mastectomy), receipt of radiotherapy (no/yes), systemic treatment modality (chemotherapy only [±anti-HER2 therapy], endocrine therapy only, or combined chemotherapy and endocrine therapy [±anti-HER2 therapy]), use of anti-HER2 therapy (no/yes) and administration of neoadjuvant chemotherapy (no/yes).

#### 2.2.3. Psychological Scales

Psychological variables were assessed using validated self-report questionnaires administered at predefined time points throughout follow-up. Relatively stable personality and psychosocial characteristics were assessed at baseline, including optimism using the Life Orientation Test–Revised [37] (LOT-R; 10 items; Cronbach’s α = 0.74), sense of coherence using the Sense of Coherence Scale [38] (SOC; 13 items assessing meaningfulness, comprehensibility and manageability; α = 0.65, 0.63 and 0.64, respectively), trait resilience using the Connor–Davidson Resilience Scale [39] (CD-RISC; 10 items; α = 0.90), dispositional mindfulness using the Mindful Attention Awareness Scale [40] (MAAS; 15 items; α = 0.87) and general coping capacity using the Perceived Ability to Cope with Trauma Scale [41] (PACT; 20 items assessing forward focus and trauma focus; α = 0.91 and 0.78, respectively). Cancer coping self-efficacy was measured using the brief Cancer Behavior Inventory [42] (CBI-B; 12 items; α = 0.89) and fear of cancer recurrence was also assessed using the Fear of Cancer Recurrence Scale–Short Form [43] (FCRI-SF; 9 items; α = 0.89), both at baseline. Finally, the cognitive strategies an individual uses to manage their emotions were measured at baseline using the Cognitive Emotion Regulation Questionnaire [44] (CERQ; 18 items assessing self-blame, other-blame, rumination, catastrophizing, putting into perspective, positive refocusing, positive reappraisal, acceptance and planning; α = 0.63, 0.71, 0.60, 0.76, 0.69, 0.75, 0.69, 0.75 and 0.66, respectively). Coping responses to cancer were assessed at three months post-baseline assessment using the Mini-Mental Adjustment to Cancer Scale [45] (mini-MAC; 29 items assessing helplessness/hopelessness, anxious preoccupation, cognitive avoidance, fighting spirit and fatalism; α = 0.88, 0.85, 0.80, 0.60 and 0.48, respectively). Post-traumatic growth was measured at three months using the Post-Traumatic Growth Inventory–Short Form [46] (PTGI-SF; 10 items assessing relating to others, new possibilities, personal strength, spiritual change and appreciation of life; α = 0.77, 0.78, 0.80, 0.78 and 0.78, respectively). Family resilience was measured with Family Resilience Questionnaire [19] (FARE; 12 items assessing communication and cohesion and family coping; α = 0.93 and 0.88, respectively) and perceived social support was measured with Modified Medical Outcomes Study Social Support Survey [47] (mMOS-SS; 8 items assessing instrumental support and emotional support; α = 0.93 and 0.86, respectively).

Anxiety symptoms were assessed every three months using the Hospital Anxiety and Depression Scale [26] (HADS; 7 items; α = 0.80 and 0.85 at baseline and month 3, respectively). Emotional functioning was assessed longitudinally every three months using the Positive and Negative Affect Schedule [48] (PANAS; 20 items assessing positive affect and negative affect; α = 0.75 and 0.82 at baseline, and 0.78 and 0.84 at month 3, respectively), while health-related QoL, including patients’ functioning status and symptom burden (e.g., arm symptoms and treatment-related side effects), was assessed every three months using the European Organisation for Research and Treatment of Cancer Quality of Life Questionnaire-Core 30 [31] (EORTC QLQ-C30; 30 items; α = 0.66–0.85 at baseline and 0.70–0.86 at month 3, except for the nausea and vomiting scale with α = 0.48 and 0.55, respectively) and its breast cancer-specific module [49] (EORTC QLQ-BR23; 23 items; α = 0.71–0.87 at baseline and 0.71–0.88 at month 3).

Several single-item measures were additionally collected to capture specific psychosocial and behavioral aspects. These included a single item assessing what patients reported doing to cope with cancer, a general self-efficacy item and a perceived social support item, all assessed every three months. Adherence to medical advice was assessed using item 5 from the Medical Outcomes Study [47] (MOS) adherence questionnaire. Single-item indicators have been shown to provide acceptable validity for clearly defined, unidimensional constructs in large survey studies while reducing participant burden and survey fatigue [50,51]. Where relevant, these brief indicators complemented established multi-item questionnaires and allowed exploratory evaluation of their predictive value within the BOUNCE study.

We retained all scales regardless of α values to maintain the clinical relevance and standardized structure of the instruments, while allowing the elastic net variable selection procedure to determine each variable’s predictive utility without risking omitted variable bias.

### 2.3. Statistical Analysis

Analyses were conducted using R version 4.3.1.

#### 2.3.1. Missing Data

Of the 538 participants included in the study, 523 (97.2%) contributed data at 3 months, 529 (98.3%) at 6 months, 509 (94.6%) at 9 months, 511 (95.0%) at 12 months, 486 (90.3%) at 15 months, and 483 (89.8%) at 18 months. Because participants could miss individual assessment waves and still contribute data at later time points, the number of observations varied across follow-up assessments. The seven assessment waves were used for trajectory clustering of the depression and quality-of-life outcomes.

The average missingness in our predictor dataset (baseline and 3-month variables) was low (3.7%). Only a small subset of variables (*n* = 7) exceeded 10% missingness, with a maximum of 22%. Missing data were addressed using multiple imputation by chained equations (mice R package, version 3.16.0 [52]). A total of 30 imputed datasets were generated, which exceeded the number required based on model-based evaluation (via the howManyImputations R package (version 0.2.5) [53]), providing a conservative margin and supporting stable estimation in the subsequent analyses. Multiple imputation approach assumes that data are missing at random (MAR), conditional on the variables included in the imputation model. To make this assumption more plausible and preserve associations among variables, all candidate predictors, outcomes and the clinical site variable were included in the imputation models [54]. A random forest-based imputation method was used to account for potential non-linear relationships and complex interactions among variables across mixed data types [55,56], which was considered advantageous given the relatively large set of candidate variables in this study.

Outcomes used for trajectory modeling were not imputed to avoid introducing artificial information that could influence trajectory estimation and class assignment, whereas missing values in predictor variables were imputed to preserve sample size and statistical power for the subsequent feature-selection analyses of latent class membership.

#### 2.3.2. Derivation of Depression and GHS/QoL Trajectories

Latent class growth analysis (LCGA) and growth mixture modeling (GMM) were conducted using the lcmm package in R (version 2.1.0) [57] to identify discrete longitudinal trajectories among breast cancer survivors based on EORTC QLQ-C30 GHS/QoL and HADS depression scores over an 18-month follow-up period. These models utilized the full longitudinal dataset by incorporating scores from all seven time points (baseline, 3, 6, 9, 12, 15, and 18 months) for both outcomes. This approach ensures that the resulting trajectories capture the continuous evolution of patient outcomes across the entire study period. Clinical or empirical thresholds and minimally important difference (MID) criteria were applied post hoc to aid the clinical interpretation of the identified trajectories. LCGA is a restricted form of GMM in which within-class variances of the random intercept and slope are fixed to zero, allowing heterogeneity only between classes. As random effects are not permitted within classes, LCGA typically requires a larger number of latent classes to adequately capture variability in the data compared with GMM.

Separate analyses were performed for each outcome, treating them as continuous variables within the latent process framework implemented in the lcmm package. To account for non-normal outcome distributions and the bounded nature of the psychometric scales, a six-knot I-spline link function was applied. This link function provides a flexible, non-linear transformation of the observed scores into a continuous latent process, effectively addressing floor and ceiling effects as well as the skewed distributions typical of the HADS and EORTC QLQ-C30 scales. The choice of link function was based on a systematic comparison of null (single-class) models using different transformations, including the beta cumulative distribution and various I-spline configurations. The six-knot I-spline was selected as it yielded the lowest Bayesian Information Criterion (BIC), indicating superior model fit (see Appendix A, Table A1, Table A2, Table A3 and Table A4 for detailed comparison).

Quadratic models of the change across time were considered. For GMM, models with one to six latent classes were estimated, while for LCGA models with one to eight latent classes were considered. Each model was estimated multiple times using a grid of 250 sets of initial values to reduce the risk of convergence to local maxima. For each specified number of latent classes, the model with the highest log-likelihood was retained.

Model selection and determination of the optimal number of latent classes were based on a combination of statistical fit indices, class separation, trajectory shapes, minimum class size, interpretability and clinical relevance [58]. Fit was assessed using the Akaike Information Criterion (AIC), Bayesian Information Criterion (BIC) and Integrated Complete Likelihood (ICL), with lower values indicating better fit [58]. Both AIC and BIC balance model fit and complexity, but BIC imposes a stronger, sample-size-dependent penalty and, therefore, typically selects more parsimonious solutions with fewer latent classes. The ICL further penalizes solutions with poor class separation, jointly accounting for model fit and classification quality. Class separation was further evaluated using entropy and average posterior class membership probabilities, with values greater than 0.60 and 0.70, respectively, indicating adequate separation [59,60]. A minimum class size of at least 5% of the total sample was required. Finally, clinical parsimony was applied to select the model that captured distinct, non-redundant trajectory patterns that were clinically meaningful for breast cancer survivors [61,62,63].

The lcmm framework utilizes maximum likelihood estimation, which inherently accommodates missing outcome data under the missing at random assumption. Therefore, no imputation of outcome variables was performed to avoid introducing artificial noise into the latent class estimation.

#### 2.3.3. Early Predictors of Depression and GHS/QoL Trajectories

Following latent class identification, patients were assigned to their most likely class based on the highest posterior probability (modal assignment). Although this approach does not explicitly account for classification uncertainty, the selected models showed adequate classification quality (entropy = 0.68 and 0.76, posterior probabilities > 0.75), suggesting sufficient class separation and likely limited bias in subsequent regression analyses [64,65].

Once the trajectory groups were identified, binomial logistic regression was conducted to determine which factors were associated with trajectory group membership. Although all seven time points were included in the LCGA/GMM models to estimate longitudinal trajectories, only baseline or 3-month variables were entered into the regression models as early predictors of group membership. Logistic regression analyses were conducted separately for each time point. By restricting predictors to data collected within the first three months, we aimed to identify early characteristics that forecast the subsequent 18-month adjustment trajectory, thereby informing timely and targeted clinical intervention. The 3-month assessment captures the patient’s initial psychological response to treatment initiation, a critical period that may strongly shape the remaining course of adjustment.

Initially, associations between individual predictors and the outcome were examined using logistic regression models adjusted for clinical site across imputations. Clinical site was included as a fixed-effect adjustment variable to account for between-site heterogeneity. To address potential quasi-complete separation, bias-reduced logistic regression was applied to categorical variables using the brglm2 R package (version 0.9.2) [66], implemented via the glm() function with method = brglmFit. All results were pooled using Rubin’s rules to obtain pooled odds ratios, 95% confidence intervals and *p*-values that reflect uncertainty due to missing data. A *p*-value < 0.05 was considered statistically significant. Given the large number of predictors evaluated, these analyses were considered exploratory and intended to provide context for individual predictor associations; therefore, no formal correction for multiple testing was applied.

Variable selection was subsequently conducted using penalized logistic regression with an elastic net penalty, implemented in R using the glmnet package (version 4.1.8) [67] (glmnet() and cv.glmnet() functions). The elastic net approach was chosen to balance variable selection and coefficient shrinkage while accommodating correlated predictors, which are common in clinical, quality-of-life, and psychosocial data. This strategy supported the identification of robust predictors while maintaining model interpretability and reducing the risk of overfitting. The elastic net mixing parameter was set to α = 0.5, providing an equal balance between L1 (LASSO) and L2 (ridge) penalties to achieve stable variable selection in the presence of correlated predictors [68]. Candidate predictors were encoded using model matrices (model.matrix), such that categorical variables were represented by indicator variables. The regularization parameter (λ) was selected via 10-fold cross-validation with stratified folds to preserve outcome class proportions within each fold and mitigate the impact of class imbalance. To promote stable and parsimonious models and reduce the risk of overfitting, the one-standard-error rule (λ_1_se) was used for λ selection [69]. Cross-validation was performed using deviance as the optimization criterion, corresponding to the negative log-likelihood of the logistic model, thereby favoring predictors that improve probabilistic calibration rather than threshold-dependent classification accuracy. The elastic net selection procedure was repeated three times for each of the 30 multiple imputed datasets generated using the mice package (see also Section 2.3.1), resulting in 90 model fits in total. A unique random seed was used for each run so that cross-validation fold assignments varied across repetitions. Predictor importance was quantified by selection frequency across imputations and repeated runs. To evaluate the robustness of our predictors, we conducted the stability analysis under two model specifications that differed in the treatment of clinical site. In the first (penalized) specification, clinical site was treated as a standard predictor subject to the same L1/L2 regularization penalty as the other variables. In the second (unpenalized) specification, clinical site was forced into the model by setting its penalty factor to zero, ensuring its inclusion in every iteration. Statistically, this treats clinical site as a fixed covariate, effectively adjusting for baseline differences in national healthcare systems, local clinical practices, socioeconomic contexts, and cultural factors. This two-specification approach allowed us to distinguish site-independent predictors from variables whose apparent importance may reflect site-specific contextual differences. Predictors with selection frequency ≥60% under both penalized and unpenalized site conditions were considered robust. Consistent with stability-selection recommendations [70], this threshold was chosen to balance the identification of stable predictors while avoiding overly conservative exclusion of relevant variables [70,71]. Penalized odds ratios were reported to indicate the direction and relative magnitude of associations at the selected regularization parameter under the initial penalized site condition. Because these estimates arise from a penalized model and are subject to shrinkage, they should be interpreted descriptively rather than as inferential effect estimates.

Model performance was evaluated using cross-validated predictions from elastic net logistic regression models, with metrics computed on held-out data and averaged across folds, thus reflecting out-of-sample performance. Performance was assessed using ROC-AUC, log-loss, and the Brier score, capturing complementary aspects of predictive quality. ROC-AUC quantifies discrimination, whereas log-loss and the Brier score assess probabilistic accuracy and calibration, with lower values indicating better performance. Although no strict cut-offs exist, ROC-AUC values of 0.60–0.70 indicate poor-to-fair discrimination, 0.70–0.80 acceptable, 0.80–0.90 very good, and >0.90 excellent discrimination [72]; Brier score and log loss were interpreted against chance-level (null-model) values, corresponding to predictions equal to the observed outcome prevalence.

Predictors were analyzed in their original measurement scales and were not standardized prior to model fitting to maintain clinical relevance. However, for penalized regression, glmnet applies internal standardization by default.

Pearson correlations among the selected predictors were examined to aid interpretation by highlighting patterns of psychological distress, symptom burden, and psychosocial resources that may jointly shape QoL or depression trajectories. Correlations were assessed across the full dataset and, therefore, reflect both associations among features and systematic differences between trajectory classes.

## 3. Results

### 3.1. Baseline Demographic and Clinical Characteristics

The study cohort comprised 538 women with a mean age of 55.4 years (40–70) recruited across four countries, most commonly Finland (38.1%), followed by Portugal (24.9%), Israel (19.3%) and Italy (17.7%) (Table 1). The majority had a university education (60.7%), were married or living with a partner (74.9%) and were employed (72.9%), with most reporting middle income levels (61.6%). Regarding lifestyle characteristics, two thirds reported engaging in exercise (66.3%), almost half followed a type of diet (45.4%), most consumed alcohol in moderation (68.2%) and the majority were never smokers (67.4%).

Clinically, most participants were postmenopausal (61.5%), had stage I–II disease (91.0%), grade II tumors (52.2%) and ductal histology (77.9%) (Table 2). Tumors were predominantly hormone receptor-positive (ER-positive 89.6% and PR-positive 79.8%) and HER2-negative (81.8%), with luminal A-like (34.9%) and luminal B-like HER2-negative (38.3%) subtypes being most frequent. Breast-conserving surgery was the most common surgical approach (74.6%), radiotherapy was administered to 80.6% of patients and systemic treatment most often consisted of endocrine therapy alone (47.3%) or combined chemotherapy and endocrine therapy (37.7%). Among patients receiving chemotherapy, the majority were treated in the adjuvant setting, while neoadjuvant chemotherapy was administered to 16% of the total sample (31% of all chemotherapy cases).

Because both neoadjuvant and adjuvant treatment pathways were included and treatment practices varied across clinical sites, the timing of the baseline assessment relative to surgery differed across participants. At the baseline assessment, 70% of patients had already undergone surgery, while some had initiated systemic therapy, including chemotherapy (7.6%), endocrine therapy (12.8%), anti-HER2 therapy (1.9%) and radiotherapy (7.0%).

### 3.2. Trajectory Groups

Trajectory clustering analyses were conducted separately for GHS/QoL and HADS depression (see Appendix B, Table A5, Table A6 and Table A7, and Appendix C, Table A8, Table A9 and Table A10 for details on model selection and classification diagnostics).

#### 3.2.1. GHS/QoL Trajectories

**Figure 1:**
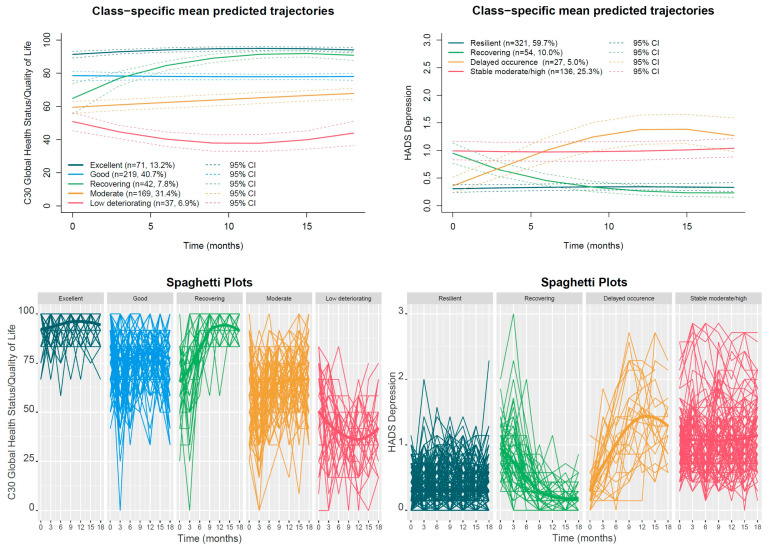
Mean growth trajectories and observed individual patient trajectories, grouped by trajectory latent classes for (**a**) EORTC QLQ-C30 Global Health Status/Quality of Life scores (0–100 scale) and (**b**) HADS depression scores (reported as mean item scores, 0–3 range). Colors indicate the different trajectory latent classes.

Both LCGA and GMM approaches were evaluated to identify latent trajectory classes for GHS/QoL. Although GMM models suggested some potential solutions, the best-fitting GMM model either included classes with very small sample sizes (<5%) or failed to recover clinically meaningful trajectories observed in LCGA. In contrast, the LCGA five-class solution balanced statistical fit, class separation, parsimony, and clinical interpretability, capturing all clinically relevant trajectory patterns with clear differentiation between classes. Therefore, all subsequent analyses for GHS/QoL trajectories are based on the five-class LCGA solution (See Appendix B for detailed explanations). Figure 1a shows the resulting trajectories for the five-group model and the actual measurements of the patients that compose each class. An excellent trajectory (*n* = 71, 13.2%) demonstrated very high baseline scores (≥90) that remained stable throughout the study period. Mean increase in slope is statistically significant (Table A11 in Appendix D) but overall changes are small (<5–10 points), indicating no clinically meaningful change over time, while individual trajectories cluster tightly at high values with limited variability. The largest class was the good trajectory (*n* = 219, 40.7%), characterized by consistently high QoL scores primarily within the good range (≥70). The mean trajectory was essentially flat (Table A11 in Appendix D). This class exhibited greater individual variability than the excellent class; however, most observations remained ≥70 throughout follow-up. The moderate class (*n* = 169, 31.4%) exhibited baseline and follow-up scores predominantly within the moderate range (50–69). Substantial within-class variability was observed, with some individuals showing improvement over time while others remained stable or fluctuated across follow-up. Overall, the mean trajectory remained largely stable (Table A11 in Appendix D). A small recovering class (*n* = 42, 7.8%) started with moderately reduced QoL (approximately 60–65) but exhibited a marked and sustained improvement exceeding clinically important change thresholds (≥10 points), reaching levels comparable to the higher-functioning classes by the end of follow-up. Most individuals in this class showed upward trajectories crossing the threshold for clinical importance. Finally, the low deteriorating class (*n* = 37, 6.9%) showed low baseline QoL near or below the poor range (<50), with further statistically important (Table A11 in Appendix D) and clinically meaningful deterioration during the early months followed by only partial recovery. The early decline exceeded 10 points, representing clinically meaningful deterioration, and subsequent improvement did not fully offset this loss. Although within-class variability was high, most individuals remained below 50–55% for much of the follow-up period.

Scores for the psychological scales subsequently selected in the elastic net stability analysis are presented in Table A13 (Appendix E) at the baseline (M0) and month 3 (M3), stratified by GHS/QoL trajectory group.

#### 3.2.2. HADS Depression Trajectories

Both LCGA and GMM models were evaluated to identify latent trajectory classes for HADS depression. While LCGA produced multiple solutions, most trajectories were nearly parallel and differed primarily in terms of baseline depression levels below the clinically meaningful threshold, providing limited additional clinical insight. In contrast, the four-class GMM solution balanced statistical fit, class separation, and clinical interpretability, capturing all clinically relevant trajectory patterns. All subsequent analyses for HADS depression trajectories are based on this four-class GMM solution (See Appendix C for detailed explanations). Figure 1b shows the resulting trajectories for the four-group model and the actual measurements for the patients that compose each class. The largest group was the resilient trajectory (*n* = 321, 59.7%), characterized by persistently very low depressive symptom levels throughout follow-up. Within this class, variability was minimal, with overall or individual mean changes being negligible or very small (Table A12 in Appendix D). The stable moderate/high trajectory (*n* = 136, 25.3%) exhibited consistently elevated depressive symptoms (mild to moderate range) that remained largely stable over time (Table A12 in Appendix D), indicating a chronic symptom burden. For most patients in this class, depressive symptom scores fluctuated considerably from one assessment to another (>0.2), yet the overall individual trajectories remained stable over time. A smaller recovering trajectory (*n* = 54, 10.0%) showed higher baseline depressive symptoms followed by a sustained and clinically meaningful decrease over time (Table A12 in Appendix D), with most individuals improving to very low symptom levels (<0.4). Finally, the delayed occurrence trajectory (*n* = 27, 5.0%) demonstrated low baseline depressive symptoms with a gradual, statistically significant (Table A12 in Appendix D) and clinically meaningful increase during follow-up, reaching levels >1, indicative of delayed onset of depressive symptomatology.

Scores for the psychological scales subsequently selected in the elastic net stability analysis are presented in Table A14 (Appendix E) at the baseline (M0) and month 3 (M3), stratified by HADS depression trajectory group.

### 3.3. Predictors of GHS/QoL Trajectories

#### 3.3.1. Low Deteriorating QoL vs. Rest

The low deteriorating QoL group (*n* = 37) was compared with the rest of the cohort (*n* = 501).

**Clinical-Site-Adjusted Univariable Analysis.** In clinical-site-adjusted univariable logistic regression analyses (Supplementary Material S1, Figure S1), higher odds of membership in the low deteriorating QoL class were observed among unemployed/housewives (OR 2.54), individuals with ≥2 negative life events (OR 4.32), those with chronic disease (OR 1.81) or mental illness (OR 4.88), higher BMI (OR 1.08 per unit), adverse tumor characteristics including stage III disease (OR 3.02), Ki-67 ≥20% (OR 2.13), triple-negative subtype (OR 3.60), and those in receipt of neoadjuvant chemotherapy (OR 4.33). In contrast, middle income (OR 0.35), physical activity (low/moderate: OR 0.23 or heavy: OR 0.12), moderate alcohol consumption (OR 0.42), estrogen receptor positivity (OR 0.28), endocrine therapy alone (OR 0.24), and combined chemo-endocrine therapy (OR 0.33) were associated with lower odds.

Both at the baseline and month 3, the vast majority of considered psychological, psychosocial, and health-related quality-of-life scales differed significantly between patients in the low deteriorating QoL class and all other classes (Supplementary Material S1, Figures S2 and S3).

Baseline psychological distress was strongly associated with higher odds, including depression (HADS; OR 6.19), anxiety (HADS; OR 3.87), fear of cancer recurrence (FCRI-SF; OR 2.60), distress (NCCN; OR 1.24), negative affect (PANAS; OR 2.41), other-blame (CERQ; OR 2,53), catastrophizing (CERQ; OR 1.45), and overall negative cognitive emotion regulation score (CERQ; OR2.76), whereas optimism (LOT-R; OR 0.39), sense of coherence (ORs 0.82–0.89), acceptance (CERQ; OR 0.70), ability to cope with trauma (trauma focus, forward focus, flexibility, and total PACT; ORs 0.60–0.78), mindfulness (MAAS; OR 0.48), resilience (CDRISC; OR 0.43), coping with cancer (CBI-B; OR 0.53), self-efficacy (OR 0.73), perceived social support (OR 0.69), positive affect (PANAS; OR 0.45), and putting into perspective (CERQ; OR 0.62) were protective. Similarly, better baseline GHS/QoL (QLQ-C30; OR 0.94), and functioning across almost all domains (physical, role, social, emotional and cognitive QLQ-C30; ORs 0.95–0.98) were associated with lower odds, while higher financial impact (QLQ-C30; OR 1.02) and symptom burden (fatigue, pain, arm symptoms, dyspnea, insomnia, appetite loss, constipation, diarrhea and systemic therapy side effects QLQ-C30 or BR23; ORs 1.01–1.05) were associated with higher odds. Moreover, better body image (QLQ-BR23; OR 0.98), future perspective (QLQ-BR23; OR 0.99), and sexual enjoyment (QLQ-BR23; OR 0.99) were associated with lower odds.

The scales not significantly associated with membership in the low deteriorating QoL class were polarity (PACT), most CERQ scales (self-blame, rumination, positive refocusing, positive reappraisal and planning; although there is a tendency to significance with the exception of planning and self-blame), as well as nausea (QLQ-C30), breast symptoms (QLQ-BR23), sexual functioning (QLQ-BR23) and upset by hair loss (QLQ-BR23).

At month 3, patients in the low deteriorating QoL class showed substantially higher psychological distress compared with all other patients, including depression (HADS; OR 6.97), anxiety (HADS; OR 5.93), distress (NCCN; OR 1.29), negative affect (PANAS; OR 2.44), anxious preoccupation (mini-MAC; OR 3.54) and helplessness/hopelessness (mini-MAC; OR 3.92). Conversely, positive affect (PANAS; OR 0.37), perceived social support (OR 0.78), general self-efficacy (OR 0.71), adaptive family communication and cohesion (FARE; ORs 0.56–0.58), personal control beliefs (OR 0.85), and positive treatment beliefs (OR 0.67) were associated with lower odds. Among coping behaviors, exercising (OR 0.62) and looking at positive aspects (OR 0.76) were protective.

Health-related QoL at month 3 similarly differentiated trajectories. Better GHS/QoL (OR 0.95) and higher functioning (physical, role, emotional, cognitive, and social functioning QLQ-C30; ORs 0.94–0.97) were associated with lower odds of low deteriorating QoL, whereas greater QLQ-C30 symptom burden—including fatigue (OR 1.04), pain (OR 1.03), dyspnea (OR 1.03), insomnia (OR 1.02), appetite loss (OR 1.02), diarrhea (OR 1.02), financial impact (OR 1.02), and treatment-related side effects (OR 1.05)—was associated with higher odds. Breast cancer-specific domains further characterized this group, including poorer body image (OR 0.98), more breast and arm symptoms (ORs 1.03), reduced future perspective (OR 0.98), and lower sexual functioning and enjoyment (ORs 0.97–0.98).

Variables related to post-traumatic growth (PTGI; relating to others, new possibilities, personal strength, spiritual change, appreciation of life, and total score), mental adjustment styles (mini-MAC; fighting spirit, avoidance, and fatalism,) and certain copying behaviors (tried to relax, distracted yourself, prayed, etc.) showed no statistically significant difference between the groups.

**Stability-Based Variable Selection.** To identify robust features associated with the low deteriorating QoL profile, we implemented a variable selection pipeline based on elastic net logistic regression across 30 multiply imputed datasets under penalized site and unpenalized site conditions. Predictor importance was quantified using selection frequency. Predictive model performance was evaluated using cross-validated predictions from elastic net logistic regression models.

A limited set of features emerged as robust in distinguishing the low deteriorating class from the remaining patients (Table 3). At baseline assessment, depressive symptoms, emotional functioning, GHS/QoL, fatigue, pain, diarrhea, sense of manageability, coping with cancer, perceived social support, and other-blame coping were the most stable predictors, each selected with 100% or near-100% (≥97%) frequency under both penalized and unpenalized clinical site conditions. Receipt of neoadjuvant chemotherapy and triple-negative molecular profile, also demonstrating high selection stability. Negative life events showed borderline selection frequency, suggesting a weaker and less consistent contribution to the model.

At month 3, cognitive functioning, depressive symptoms, physical functioning, and treatment control beliefs emerged as the most robust predictors, each being selected in 100% of the 90 stability iterations under both penalized and unpenalized clinical site conditions. These were followed by anxiety symptoms and family communication and cohesion. In contrast, neoadjuvant chemotherapy showed unstable selection when the clinical site was forced into the model.

Both at the baseline and month 3, the models demonstrated strong discriminative ability (ROC-AUC ~ 0.86) and high probabilistic accuracy (for chance performance log-loss = 0.253 and Brier score = 0.064) (Table 3).

#### 3.3.2. Excellent QoL vs. Rest

The excellent QoL group (*n* = 71) was compared with the rest of the cohort (*n* = 467).

**Clinical-Site-Adjusted Univariable Analysis.** Compared with all other classes (Supplementary Material S1, Figure S4), excellent QoL was associated with older age (OR 1.03), heavy physical activity (OR 2.55), lower BMI (OR 0.93), no negative life events (ORs 0.37–0.18), and absence of mental illness (OR 0.13). Favorable disease characteristics, including lower stage (ORs 0.59–0.25), lower grade (ORs 0.50–0.35), low Ki-67 (OR 0.53) and luminal A-like subtype (OR 2.11), were also associated with excellent QoL (note that odds ratios below 1 reflect the coding of the variables and should be interpreted accordingly). In contrast, neoadjuvant chemotherapy was inversely associated (OR 0.18), whereas endocrine therapy alone was positively associated (OR 2.51) with excellent QoL.

At the baseline and month 3, almost all considered psychological, psychosocial, and health-related quality-of-life scales differed significantly between patients in the excellent QoL class and all other classes, with consistent advantages observed across distress, coping, affect, functioning, and symptom domains (Supplementary Material S1, Figures S5 and S6).

At the baseline, the excellent QoL group was less likely to report negative psychological traits, including depressive symptoms (HADS; OR = 0.06), anxiety (HADS; OR = 0.14), fear of recurrence (FCRI-SF; OR = 0.42), self-blame (CERQ; OR = 0.53), other-blame (CERQ; OR = 0.59), catastrophizing (CERQ; OR = 0.38), and overall negative cognitive emotion regulation strategies (CERQ; OR = 0.29).

Conversely, the excellent QoL group was more likely to report positive psychological traits, including optimism (LOT-R; OR = 2.36); comprehensibility (SOC; OR = 1.16), manageability (SOC; OR = 1.15), and meaningfulness (SOC; OR = 1.18); positive coping strategies (PACT: forward focus OR = 1.56, trauma focus OR = 1.72, and total coping OR = 1.36); adaptive emotion regulation strategies (CERQ: perspective-taking OR = 1.43, positive refocusing OR = 1.57, positive reappraisal OR = 1.37, and acceptance OR = 1.41); mindfulness (MAAS; OR = 2.65); resilience (CDRISC; OR = 3.89); and positive affect (PANAS; OR = 2.97).

Moreover, better baseline GHS/QoL (OR = 1.14) and better functioning across all QLQ-C30 domains, including physical (OR = 1.09), role (OR = 1.05), emotional (OR = 1.07), cognitive (OR = 1.06), and social functioning (OR = 1.05), were associated with higher odds of belonging to the excellent QoL class. In contrast, greater symptom burden, including fatigue (OR = 0.95), nausea (OR = 0.92), pain (OR = 0.95), dyspnea (OR = 0.97), insomnia (OR = 0.98), appetite loss (OR = 0.97), constipation (OR = 0.97), and diarrhea (OR = 0.96) (QLQ-C30), as well as systemic therapy side effects (QLQ-BR23; OR = 0.92), breast symptoms (OR = 0.98), and arm symptoms (OR = 0.96), was associated with lower odds of excellent QoL. Moreover, better body image (QLQ-BR23; OR = 1.04), future perspective (QLQ-BR23; OR = 1.03), sexual functioning (QLQ-BR23; OR = 1.01), and sexual enjoyment (QLQ-BR23; OR = 1.01) were associated with higher odds of excellent QoL.

Rumination (CERQ), planning (CERQ), polarity (PACT) and upset by hair loss (QLQ-BR23) did not show a statistically significant difference between the two groups.

At month 3, individuals in the excellent QoL group showed significant differences across a wide range of psychosocial and coping variables. They were significantly more likely to report strong family communication and cohesion (FARE; OR = 2.43), effective family coping (FARE; OR = 2.17), higher general self-efficacy (single item; OR = 1.76), better adherence to medical advice (OR = 1.56), greater perceived social support (single item; OR = 1.52), instrumental support (mMOS-SS; OR = 1.53), emotional support (mMOS-SS; OR = 2.12), and overall social support (mMOS-SS; OR = 2.01). They also reported higher positive affect (PANAS; OR = 5.46), stronger personal control beliefs (OR = 1.28), treatment control beliefs (OR = 1.36), and greater use of exercise as a coping behavior (OR = 1.34).

Conversely, the excellent QoL group was significantly less likely to report depressive symptoms (HADS; OR = 0.03); anxiety (HADS; OR = 0.07); overall mental health distress (HADS total; OR = 0.02); general distress (NCCN Distress Thermometer; OR = 0.62); helplessness/hopelessness (mini-MAC; OR = 0.15); anxious preoccupation (mini-MAC; OR = 0.21); avoidance coping (mini-MAC; OR = 0.56); negative affect (PANAS; OR = 0.15); and the coping behaviors of crying (OR = 0.47), talking to the physician (OR = 0.62) or ask for help (OR 0.79).

All health-related QoL domains at month 3 differentiated between trajectory groups. Better GHS/QoL and higher overall functioning were associated with a greater likelihood of belonging to the excellent QoL class (ORs 1.05–1.16), whereas greater symptom burden and financial impact were associated with lower odds (ORs 0.93–0.98). Breast cancer-specific domains further contributed to this differentiation, with better body image, future perspective, and sexual functioning (ORs 1.01–1.04) and fewer treatment-related, breast, and arm symptoms (ORs 0.94–0.98) characterizing the excellent QoL group.

Variables related to post-traumatic growth (PTGI; relating to others, new possibilities, personal strength, spiritual change, appreciation of life, and total PTGI score), specific mental adjustment styles (mini-MAC; fighting spirit and fatalism), and certain coping behaviors (e.g., trying to relax, distraction, praying, and looking at positive sides and perceiving the situation as a challenge) showed no statistically significant differences between the groups.

**Stability-Based Variable Selection.** Stability selection at the baseline and month 3 identified a relatively broad set of factors distinguishing the excellent QoL class (Table 4).

At baseline assessment, robust predictors were associated with psychological resources and lower distress, including lower anxiety and higher mindfulness, resilience, coping, self-efficacy, and perceived social support. Better GHS/QoL and functioning across physical, role, emotional, and cognitive domains, together with lower catastrophizing and symptom burden, further differentiated the excellent QoL class. In addition, treatment-related factors (receipt of endocrine therapy and absence of neoadjuvant chemotherapy), favorable molecular phenotypes (more luminal A-like and less luminal B-like [HER2+]), and selected sociodemographic characteristics (not being unemployed/housewife or following a vegetarian diet) contributed to class differentiation. Notably, the stability of pre-existing mental illness and positive affect dropped well below 60% when clinical site was forced into the model.

At month 3, robust predictors reflected lower psychological distress and treatment-related symptom impact, including lower anxiety, anxious preoccupation, fatigue, systemic therapy side effects, and distress, alongside greater positive affect, future perspective, and role and social functioning, as well as less use of the coping strategy of talking to the physician. These variables were selected in 100% or near-100% of stability iterations. Additional contributors included better physical and emotional functioning, stronger personal control beliefs, greater perceived social and emotional support, lower pain and arm symptoms, fewer depressive symptoms, better family communication and cohesion, and reduced exposure to multiple negative life events. In contrast, the stability of negative affect declined markedly when clinical site effects were accounted for.

Both at baseline and month 3, the models demonstrated strong discriminative ability and high probabilistic accuracy (for chance performance log-loss = 0.390 and Brier score = 0.115) (Table 4).

#### 3.3.3. Recovering vs. Moderate QoL

The recovering QoL group (*n* = 42) was compared with the moderate QoL group (*n* = 169).

**Clinical-Site-Adjusted Univariable Analysis.** Among sociodemographic, lifestyle, clinical, and cancer-related factors, clinical-site-adjusted univariable logistic regression analyses (Supplementary Material S1, Figure S7) identified exposure to negative life events as the only factor significantly associated with QoL recovery, with lower odds observed across increasing exposure levels (OR range = 0.17–0.30).

Several baseline (M0) factors were significantly associated with QoL recovery. Protective factors included higher optimism (LOT-R; OR = 2.72), greater resilience (CD-RISC; OR = 2.42), greater coping flexibility (PACT; OR = 1.23), and greater use of adaptive cognitive emotion regulation strategies, such as perspective-taking (CERQ; OR = 1.57) and planning (CERQ; OR = 1.72), all of which were associated with increased odds of recovery. In contrast, risk factors included higher depressive symptoms (HADS; OR = 0.40), anxiety symptoms (HADS; OR = 0.46), and greater use of catastrophizing (CERQ; OR = 0.63), which were associated with lower odds of QoL recovery (Supplementary Material S1, Figure S8). Regarding health-related QoL domains at baseline, better GHS/QoL (OR = 1.03) and higher functioning, including physical (OR = 1.03), role (OR = 1.02), emotional (OR = 1.02), and social functioning (all QLQ-C30; OR = 1.03), were associated with a greater likelihood of belonging to the recovering QoL class. In contrast, higher symptom burden, particularly fatigue (QLQ-C30; OR = 0.97) and pain (QLQ-C30; OR = 0.97), was associated with lower odds of recovery. Among breast cancer-specific domains, better sexual functioning (QLQ-BR23; OR = 1.02) was positively associated with QoL recovery, whereas most symptom and body image domains did not significantly differentiate recovering trajectories.

The psychological scales not significantly associated with membership in the recovering QoL class included sense of comprehensibility, manageability, and meaningfulness (SOC); fear of recurrence (FCRI-SF); trauma and total coping (PACT), with polarity coping showing only a borderline association; most cognitive emotion regulation strategies (CERQ), including self-blame, other-blame, rumination, positive reappraisal, acceptance, and negative overall CERQ (with borderline effects for positive refocusing and acceptance); distress (NCCN Distress Thermometer); and negative affect (PANAS).

At month 3, site-adjusted models indicated that lower symptom burden and more adaptive coping remained associated with QoL recovery. Specifically, lower helplessness/hopelessness (mini-MAC; OR = 0.13), lower anxious preoccupation (mini-MAC; OR = 0.34), lower anxiety and depressive symptoms (HADS; ORs = 0.12–0.21), lower distress (NCCN; OR = 0.86), lower negative affect (PANAS; OR = 0.52), higher fighting spirit (mini-MAC; OR = 3.93), higher positive affect (PANAS; OR = 3.46), greater general self-efficacy (OR = 1.74), stronger personal and treatment control beliefs (ORs = 1.39–1.78), higher family coping (FARE; OR = 1.82) and greater emotional support (mMOS-SS; OR = 2.01) were significant predictors of recovery. In addition, better GHS/QoL and higher functioning (physical, role, emotional, cognitive, and social; QLQ-C30; ORs = 1.02–1.05); lower pain (QLQ-C30; OR = 0.97), fatigue and nausea (QLQ-C30; ORs = 0.97–0.98); and better sexual functioning, enjoyment, and future perspective (QLQ-BR23; ORs = 1.02–1.03) were also associated with increased odds of QoL improvement (Supplementary Material S1, Figure S9) (note that odds ratios below 1 reflect the coding of the variables and should be interpreted in the context of symptom direction; lower scores indicate better outcomes).

At month 3, the scales that did not differ between the groups were post-traumatic growth (PTGI; relating to others, new possibilities, personal strength, spiritual change, appreciation of life, and total PTGI score), mental adjustment styles reflecting avoidance and fatalism (mini-MAC), family communication and cohesion (FARE), adherence to medical advice, several coping behaviors (including trying to relax, distraction, praying/going to church, exercising, bursting into tears, talking to or asking for help from someone important, and talking to the physician), and instrumental social support (mMOS-SS). In terms of health-related QoL, dyspnea, insomnia, constipation, diarrhea, and financial impact (EORTC QLQ-C30), as well as breast symptoms, arm symptoms, and upset by hair loss (QLQ-BR23), did not significantly differ between groups.

**Stability-Based Variable Selection.** A concise set of features emerged as robust in terms of distinguishing the recovering class from the moderate QoL one (Table 5). At baseline assessment, coping with cancer, mindfulness, optimism, perspective-taking emotion regulation, pain, positive affect, sexual functioning, and social functioning, together with middle- and high income (vs. low income), emerged as the most robust predictors, having a selection frequency of 100% or above 90%. These were followed by planning as a cognitive emotion regulation strategy and exposure to two or more negative life events. The stability of resilience, postmenopausal, and adherence to a special diet dropped markedly when clinical site was forced into the model. No psychological distress symptoms emerged as robust predictors.

At month 3, a similarly concise set of features robustly distinguished the recovering QoL class from the rest of the patients (Table 5). Helplessness/hopelessness, pain, positive affect, sexual functioning, personal control beliefs over illness, middle- and high income (vs. low income), coping by perceiving the situation as a challenge and general self-efficacy were the most stable predictors, each selected in 100% or nearly 100% of the stability iterations, even when clinical site was forced in the model. These were followed by social functioning, fighting spirit, depression and anxiety symptoms. The selection frequency of postmenopausal status, negative life events and triple-negative disease dropped below the threshold when clinical site was forced into the model.

At baseline assessment, the model showed fair discrimination and moderate probabilistic accuracy (Table 5; for chance performance log-loss = 0.500 and Brier score = 0.159). At month 3, performance improved, with good discrimination and better calibration and accuracy, indicating enhanced predictive ability over time. Together, these results suggest that incorporating baseline and month 3 information would substantially enhances the model’s ability to predict QoL trajectories compared with baseline alone.

### 3.4. Predictors of HADS Depression Trajectories

#### 3.4.1. Stable Moderate/High vs. Resilient

The stable moderate/high depression group (*n* = 136) was compared with the resilient group (*n* = 321).

**Clinical-Site-Adjusted Univariable Analysis.** Compared with the resilient class, several protective factors were associated with lower odds of belonging to the stable moderate/high depression class, including clinical site in Finland (vs. Portugal) (OR = 0.44), university education (OR = 0.52), being married or in a common-law relationship (vs. single) (OR = 0.47), higher income (OR = 0.39), postmenopausal status (OR = 0.59), engagement in physical activity (heavy: OR = 0.33), and moderate alcohol consumption (OR = 0.49). In contrast, risk factors associated with higher odds of stable moderate/high depression included clinical site in Italy (vs. Portugal) (OR = 3.51), unemployment or being a housewife (OR = 2.54), exposure to two or more negative life events (OR = 2.31), history of mental illness (OR = 3.93), HER2-positive (non-luminal) tumor subtype (OR = 3.57), and receipt of neoadjuvant chemotherapy (OR = 2.17) (Supplementary Material S1, Figure S10).

At baseline, the scales that did not show statistically significant differences between the resilient and stable moderate/high depression groups were self-blame (CERQ) and acceptance (CERQ), with other-blame (CERQ) showing only a borderline association. All other psychological, coping, and affective scales, as well as all health-related quality-of-life domains were significantly associated with group membership (Supplementary Material S1, Figure S11).

At month 3, the scales not significantly associated with group membership between the resilient and stable moderate/high depression classes were post-traumatic growth dimensions (PTGI; relating to others, new possibilities, personal strength, appreciation of life, and total PTGI score), with the exception of spiritual change, fatalism (mini-MAC), and a few coping behaviors (trying to relax, praying/going to church, talking to or asking for help from somebody important). In terms of health-related QoL, only diarrhea (QLQ-C30) did not significantly differ between the groups. All other psychological, coping, social support, and QoL scales demonstrated statistically significant associations (Supplementary Material S1, Figure S12).

**Stability-Based Variable Selection.** The variable selection procedure identified a concise set of variables that robustly distinguished the stable moderate/high depression class from the resilient class (Table 6). At baseline, risk factors consistently selected across all stability iterations included higher anxiety and depressive symptoms, greater arm symptoms, higher financial impact, poorer future perspective, greater catastrophizing, lower sense of manageability and meaningfulness, lower optimism, poorer role functioning, and clinical site in Italy or Finland (vs. Portugal). Additional variables distinguishing the stable moderate/high class were higher distress and lower resilience. Stability of coping with cancer, heavy exercise (vs. none), unemployment or being a housewife (vs. employment) dropped to 0% when clinical site was forced into the model.

At month 3, variables consistently selected across all stability iterations and penalized clinical site conditions included anxiety, distress, anxious preoccupation, negative affect, helplessness/hopelessness, emotional functioning, future perspective, positive affect, greater emotional support and clinical site in Italy or Finland (vs. Portugal). Additional selected variables included fatigue, pain, arm symptoms, spiritual change, sexual functioning and enjoyment and cognitive functioning. In contrast, the selection frequency of avoidance coping, radiotherapy, university education, use of exercise as a coping strategy and heavy physical activity dropped considerably, even to 0, when clinical site was forced to the model.

At baseline, the model demonstrated excellent discrimination and calibration performance, while at month 3, performance remained high, though slightly reduced, indicating robust predictive ability at both time points (Table 6; for chance performance log-loss = 0.610 and Brier score = 0.209). The high discriminative performance of the baseline model (AUC = 0.94) likely reflects the strong prognostic value of initial symptom severity. Although inclusion of baseline measures of the outcome may contribute to higher performance metrics due to the inherent relationship between baseline scores and subsequent trajectories, baseline levels of the outcome are clinically meaningful and widely used in prognostic modeling. Consistent across our analyses, baseline measures were retained to maximize the model’s clinical relevance and real-world applicability.

#### 3.4.2. Delayed Occurrence vs. Resilient

The delayed occurrence depression group (*n* = 27) was compared with the resilient group (*n* = 321).

**Clinical-Site-Adjusted Univariable Analysis.** Regarding sociodemographic, lifestyle and clinical data, compared with the resilient class, several risk factors were associated with a higher likelihood of belonging to the delayed occurrence depression class (Supplementary Material S1, Figure S13). These included history of mental illness (OR 29.50), HER2-positive tumors (OR 3.00), triple-negative breast cancer subtype (OR 3.69), and receipt of anti-HER2 therapy (OR 2.95). In contrast, several protective factors were associated with lower odds of delayed depression, including clinical site in Finland (vs. Portugal) (OR 0.17), university education (OR 0.29), middle income (vs. low income) (OR 0.23), engagement in physical activity (low–moderate: OR 0.34; heavy: OR 0.23), never having been a smoker or being a former smoking (ORs 0.24–0.31), and estrogen receptor-positive disease (OR = 0.29).

At baseline assessment, poorer psychological resources and QoL were significantly associated with higher odds of belonging to the delayed occurrence depression class. Specifically, lower optimism (LOT-R; OR = 0.34); lower sense of coherence, including manageability (SOC; OR = 0.86) and meaningfulness (SOC; OR = 0.85); lower resilience (CD-RISC; OR = 0.36); and reduced use of positive cognitive emotion regulation strategies, including positive refocusing (CERQ; OR = 0.64) and lower overall positive CERQ (OR = 0.56), were associated with increased risk. In terms of health-related QoL, worse GHS/QoL (EORTC QLQ-C30; OR = 0.97), poorer physical and role functioning (ORs = 0.96–0.97), and greater symptom burden, namely fatigue (OR = 1.03), pain (OR = 1.05), insomnia (OR = 1.02), appetite loss (OR = 1.03), diarrhea (OR = 1.04), and financial impact (OR = 1.03), were also significantly associated with delayed depression, along with greater arm symptoms (QLQ-BR23; OR = 1.04) (Supplementary Material S1, Figure S14).

At the baseline, core affective symptoms were not significantly associated with delayed depression, including depressive symptoms, anxiety symptoms, and overall mental health distress (HADS). Several coping styles and emotion regulation strategies also showed no significant differences, including forward focus, trauma focus, total coping, polarity coping, and flexibility coping (PACT), self-blame, other-blame, rumination, catastrophizing, perspective-taking, acceptance, planning, and overall negative CERQ, as well as mindfulness (MAAS), coping with cancer (CBI-B), general self-efficacy, and perceived social support. In addition, fear of cancer recurrence (FCRI-SF), distress thermometer scores (NCCN), negative affect (PANAS), and most breast cancer-specific QoL domains, including body image, treatment side effects, breast symptoms, sexual function, sexual enjoyment, and upset by hair loss (QLQ-BR23), were not significantly associated. Among EORTC QLQ-C30 domains, cognitive and social functioning, dyspnea, nausea, and constipation did not differ significantly between groups.

At month 3, psychological distress and affective symptoms were strongly associated with higher odds of belonging to the delayed occurrence of depression class, including depressive symptoms (HADS; OR = 9.50), anxiety symptoms (HADS; OR = 5.40) and overall mental health distress (HADS total; OR = 17.51). In contrast, lower positive affect (PANAS; OR = 0.34) and lower emotional and total social support (mMOS-SS; ORs = 0.56) were associated with reduced odds of delayed depression. Maladaptive coping and behavioral responses were also relevant, with coping by bursting into tears (OR = 1.57) and talking to the physician (OR = 1.69) associated with higher odds (Supplementary Material S1, Figure S15).

In terms of health-related QoL, poorer GHS/QoL (QLQ-C30; OR = 0.97), lower physical, role, emotional, and cognitive functioning (ORs = 0.96–0.98), and greater symptom burden, including nausea (OR = 1.03), pain (OR = 1.02), appetite loss (OR = 1.02), diarrhea (OR = 1.03), and financial impact (OR = 1.02), were significantly associated with delayed depression. Breast cancer-specific domains further characterized this group, including greater arm symptoms (QLQ-BR23; OR = 1.03) and poorer future perspective, sexual functioning, and sexual enjoyment (QLQ-BR23; ORs = 0.97–0.98).

At month 3, several domains did not significantly differentiate the delayed depression and resilient groups, including post-traumatic growth (PTGI), most mental adjustment styles (mini-MAC), family functioning (FARE; although it tends to significance), and general coping resources and behaviors (adherence to medical advice, general self-efficacy, perceived support, and most specific coping strategies). In terms of health-related QoL, social functioning, fatigue, dyspnea, insomnia, and constipation (EORTC QLQ-C30), as well as breast cancer-specific domains such as breast symptoms, and upset by hair loss (QLQ-BR23), were not significantly associated.

**Stability-Based Variable Selection.** At baseline assessment, a limited set of features emerged from the stability-selection pipeline as robust discriminators between the delayed depression occurrence and resilient groups (Table 7). Among these, only diarrhea, pain, and role functioning demonstrated robust selection, each with 100% or near 100% selection frequency under both penalized and unpenalized clinical site conditions. In contrast, the selection stability of sense of manageability and optimism declined substantially when clinical site was forced into the model.

At month 3, a broader range of factors differentiated the delayed depression trajectory. Variables showing high selection frequency under both penalized and unpenalized site conditions included diarrhea, emotional functioning, anxiety symptoms, pre-existing mental illness, university education, and coping through communication with the physician, followed by sexual functioning and triple-negative disease. Middle income (vs. low income) showed borderline selection stability, with selection frequency decreasing to approximately 40% after forcing clinical site into the model, suggesting that this variable may partly reflect site-related differences. In contrast, the stability of pain, unemployment/housewife status and heavy exercise declined markedly once clinical site was unpenalized, with selection frequencies dropping to approximately 10% and 0%, respectively, indicating that their apparent predictive signal was largely explained by site-related differences.

At baseline assessment, the model showed good discrimination and calibration, while at month 3, performance remained stable, with similar probabilistic accuracy and a slightly lower discrimination (Table 7; for chance performance log-loss = 0.273 and Brier score = 0.072).

#### 3.4.3. Recovering vs. Stable Moderate/High

The recovering depression group (*n* = 54) was compared with the stable moderate/high group (*n* = 136).

**Clinical-Site-Adjusted Univariable Analysis.** Compared with the stable moderate/high depression class, predictors of belonging to the recovering depression class included HER2-positive disease (OR 2.50), and luminal B-like (HER2-positive) tumor subtype (OR = 3.77) (Supplementary Material S1, Figure S16). In contrast, lower odds of belonging to the recovering class were associated with clinical site in Italy (vs. Portugal) (OR = 0.32), adherence to a special diet (vs. none; OR = 0.23), higher BMI (OR = 0.91 per unit increase), one negative life event (OR = 0.32), metabolic disease (OR = 0.27), luminal A-like subtype (OR = 0.43), and receipt of endocrine therapy only (vs. chemotherapy only ± anti-HER2; OR = 0.38).

At the baseline, predictors of belonging to the recovering depression class included higher optimism (LOT-R; OR = 2.44), greater sense of coherence–manageability (SOC; OR = 1.15), greater sense of coherence–meaningfulness (SOC; OR = 1.13), higher mindfulness (OR = 1.87), higher positive affect (OR = 1.90), and lower pain (OR = 0.98) (Supplementary Material S1, Figure S17).

At month 3, predictors of belonging to the recovering depression class included lower depressive symptoms (OR = 0.24), lower anxiety symptoms (OR = 0.17), better overall mental health (OR = 0.13), lower spiritual change (PTGI; OR = 0.70), lower helplessness/hopelessness (mini-MAC; OR = 0.36), lower anxious preoccupation (mini-MAC; OR = 0.47), lower distress (NCCN Distress Thermometer; OR = 0.86), higher perceived social support (OR = 1.31), greater treatment control beliefs (OR = 1.28), higher positive affect (OR = 2.19), lower negative affect (OR = 0.49), greater instrumental support (OR = 1.54), greater emotional support (OR = 2.04), greater total social support (OR = 1.90), better emotional functioning (OR = 1.04), better cognitive functioning (OR = 1.02), lower pain (OR = 0.98), lower insomnia (OR = 0.98), fewer arm symptoms (OR = 0.97), and a more positive future perspective (EORTC QLQ-BR23; OR = 1.01) (Supplementary Material S1, Figure S18).

**Stability-Based Variable Selection.** Based on the variable selection method, retained variables at the baseline when clinical site was penalized were: sense of manageability, optimism, clinical site (Italy vs. Portugal), treatment type (endocrine therapy only vs. chemotherapy ± anti-HER2), and high income (vs. low income), which were all selected in 100% of the stability iterations, followed by clinical site in Finland (vs. Portugal), which was selected in 60% of iterations.

At month 3, variables selected in 100% of the stability iterations under penalized site conditions included anxiety symptoms, clinical site (Italy vs. Portugal), treatment type (endocrine therapy only vs. chemotherapy ± anti-HER2), and high income (vs. low income). Variables with high but slightly lower stability included spiritual change, following a special diet, and coping by talking to someone important (each selected in 90% of iterations), as well as emotional functioning. Additional contributors included upset by hair loss, metabolic diseases, clinical site in Finland (vs. Portugal), negative affect, and emotional support (Table 8).

While optimism, income (baseline), endocrine only treatment (baseline and month 3), coping by talking to someone important (month 3), upset by hair loss (month 3) and negative affect (month 3) initially demonstrated high selection stability (in the clinical-site-penalized model), their selection frequencies dropped dramatically when site was unpenalized (i.e., forced into the model). This pattern suggests that the predictive contribution of these variables may reflect site-related differences rather than site-independent effects.

The selection frequency for spiritual change (month 3) decreased from 90% (penalized site) to 40% (unpenalized site) and for high income (month 3) from 100% (penalized site) to 57% (unpenalized site). This indicates that while site variation explains a significant portion of the effect of these variables, a moderate, site-independent effect remains, suggesting this variable possesses a more robust, universal association with the outcome compared to other site-dependent variables.

Although stability analysis identified a consistent set of predictors, overall model discrimination at the baseline and month 3 was modest (Table 8; for chance performance log-loss = 0.596 and Brier score = 0.204), indicating limited predictive capacity. Accordingly, stability-selected variables should be interpreted as robust correlates of the recovering depression profile.

### 3.5. Correlation Structure of Predictors Selected by Stability Analysis

Correlation matrices were computed for predictors selected in the stability analysis at the baseline and month 3 (Supplementary Material S2). Considering strong correlations (*r* ≥ 0.50; Cohen’s criteria), several clusters emerged. At the baseline, strong intercorrelations were observed among psychological distress variables (depression, anxiety, distress thermometer, and negative affect; *r* ~ 0.57–0.75), physical symptoms (fatigue, pain, and systemic therapy side effects; *r* ~ 0.50–0.61), positive psychological resources (resilience, coping with cancer, self-efficacy, and optimism; *r* ~ 0.50–0.63) and functioning variables (physical, role, and social; *r* ~ 0.50 and above). Functioning variables showed strong negative correlations (*r* around −0.50) with symptom clusters and strong positive correlations with GHS/QoL (*r* ~ 0.50). Distress variables also showed strong negative correlations with emotional functioning (*r* ~ −0.57–0.69), future perspective (*r* ~ −0.5) and coping with cancer (*r* ~ −0.50–0.57). A similar pattern was observed at month 3, with strong associations among distress variables (anxiety, depression, distress thermometer, negative affect, helplessness/hopelessness, anxious preoccupation, poor emotional functioning, and low future perspective; *r* ~ 0.50–0.74), among physical symptoms (fatigue, pain, and systemic therapy side effects; *r* ~ 0.50–0.62), among functioning and quality-of-life measures (physical, role, social, and GHS/QoL; ~0.56–0.72), and between social, emotional support and family communication/cohesion (~0.5), while functioning cluster remained negatively related to physical symptoms (*r* ~ 0.5–0.71) and the depression component (*r* ~ −0.50).

## 4. Discussion

Using latent class growth analysis (LCGA) and growth mixture modeling (GMM), we identified distinct trajectories of GHS/QoL and HADS depression among women with early breast cancer over an 18-month period following the baseline assessment, highlighting clinically meaningful heterogeneity in longitudinal patient-reported outcomes.

Five distinct trajectories of GHS/QoL were identified. Two trajectories reflected stable high functioning, comprising the Good (40.7%) and Excellent (13.2%) groups, whereas nearly one-third of participants belonged to a Moderate trajectory (31.4%) characterized by persistently intermediate QoL. A small recovering group (7.8%) demonstrated clear and clinically meaningful improvement over time. Finally, a small but clinically vulnerable subgroup (low deteriorating, 6.9%) experienced sustained poor QoL with early deterioration and limited recovery.

Park et al. (2023) [73] identified three largely stable QoL trajectories over the first year following the end of primary treatment among breast cancer survivors (*N* = 124). The majority of women maintained relatively good QoL over time, while a smaller subgroup reported persistently low QoL following treatment. Di Meglio et al. (2022) [74] identified distinct long-term QoL trajectories among women with stage I–III breast cancer treated with adjuvant chemotherapy (*N* = 4131), including stable excellent and very good patterns, as well as notably smaller persistently poor and deteriorating trajectories extending up to four years after diagnosis. Goyal et al. (2018) [75] examined QoL over an 18-month period following the baseline assessment (conducted within eight months after diagnosis) among women with newly diagnosed stage I–III breast cancer (*N* = 653) and identified six trajectories. These included persistently low or very low QoL trajectories, trajectories characterized by moderate or high QoL, as well as two improving trajectories leading to moderate or high QoL, similar to our study.

In the case of depressive symptoms, we identified four distinct trajectories, underscoring clinically meaningful heterogeneity in the longitudinal course of depression. The majority of participants followed a resilient trajectory (59.7%), characterized by persistently low depressive symptom levels. A substantial proportion belonged to a stable moderate/high trajectory (25.3%), marked by consistently elevated symptoms indicative of a chronic depressive burden. A smaller recovering group (10.0%) demonstrated clear and clinically meaningful improvement over time, with symptoms declining to low levels. Finally, a small but clinically vulnerable delayed occurrence subgroup (5.0%) experienced a gradual and clinically meaningful increase in depressive symptoms during follow-up, reflecting delayed onset of depression.

In a cohort of 4803 women with stage I–III breast cancer, Charles et al. (2022) [76] examined the trajectories of depressive symptoms measured using the Hospital Anxiety and Depression Scale (HADS) over the three years following diagnosis. Six distinct trajectory groups were identified, ranging from persistent non-cases to stable depression. Remission and delayed-onset patterns were identified that comprised only a small proportion of patients, consistent with the findings of the present study. Kant et al. (2018) [77] identified four distress trajectories among 181 newly diagnosed breast cancer patients over the first six months following surgery. A resilient trajectory comprised the majority of patients, whereas high-remitting, delayed, and chronic distress trajectories accounted for smaller proportions, similar to the pattern observed in our study. In 300 Chinese women with breast cancer, Li et al. (2022) [78] reported stable none/mild, stable low and high-decreasing depressive symptom trajectories over 18 months after discharge.

Across these studies, similar to our findings, stable good-QoL or depression trajectories typically comprised the largest proportion of patients, whereas deteriorating or persistently poor QoL trajectories consistently represented smaller but clinically vulnerable subgroups. Differences across studies in the cohort size, baseline timing (before or after the end of primary treatment), study duration, assessment intervals and modeling approach and model selection criteria likely explain the observed variation in the number and type of trajectories reported in the literature, as well as compared to our findings.

To identify parsimonious sets of predictors that jointly differentiate longitudinal quality-of-life and depression trajectory groups, we combined clinically interpretable logistic regression models with multivariable variable selection using elastic net regularization. Following trajectory clustering, individual trajectory classes were retained either to capture nuanced patterns of change in QoL and depressive symptoms over time or to conduct clinically motivated binary contrasts that support risk stratification and interpretability. For QoL, comparisons included low deteriorating versus remaining trajectories, excellent versus remaining trajectories, and recovering versus moderate trajectories, the latter with comparable baseline GHS/QoL levels. For depression, analyses contrasted the resilient class with the stable moderate/high depression class, the delayed depression occurrence class with the resilient class, and the recovering class with the stable moderate/high depression class. Importantly, all analyses were conducted under two conditions, with the clinical site variable alternatively penalized and left unpenalized, to assess the robustness of predictor selection to potential site effects. Elastic net-penalized logistic regression enabled simultaneous variable selection and coefficient shrinkage, allowing identification of robust predictors while accounting for multicollinearity among candidate psychosocial, clinical, and sociodemographic factors.

Based on clinical-site-adjusted univariable logistic regression analyses, the low deteriorating and excellent QoL trajectories appeared largely as mirror images, with significant differences across most examined variables. However, elastic net feature selection retained only a smaller subset of predictors, suggesting that only a more limited number of factors independently and robustly distinguish QoL trajectories when multiple variables are considered simultaneously.

Low deteriorating QoL: At baseline assessment, before most patients initiated systemic therapy or radiotherapy, the low deteriorating QoL trajectory reflected a combination of psychological vulnerability (depressive symptoms and poorer emotional functioning), reduced psychosocial resources (lower coping self-efficacy, perceived manageability, and social support), maladaptive coping strategies (particularly other-blame), higher symptom burden (fatigue, pain, and diarrhea), and adverse clinical characteristics (neoadjuvant chemotherapy and triple-negative disease).

The selection of both coping self-efficacy and perceived manageability suggests that this trajectory reflects reduced complementary resilience resources, characterized by lower confidence in managing cancer-related challenges and diminished perceptions of available personal and social resources to cope with life demands. The strong negative correlations of depression with emotional functioning and coping self-efficacy is consistent with a psychological vulnerability pattern in which heightened distress co-occurs with reduced perceived coping capacity. In parallel, the strong association between fatigue and pain indicates the presence of a physical symptom cluster at treatment initiation. These two clusters of psychological distress and physical symptoms showed only low to moderate correlations between them, suggesting partially independent dimensions influencing QoL trajectories. Additionally, other-blame emerged as a relatively independent maladaptive coping strategy. The selection of neoadjuvant chemotherapy and triple-negative disease likely reflects greater perceived disease severity and anticipatory treatment-related distress rather than early treatment toxicity and functional decline.

At month 3, during active treatment, predictors of the low deteriorating trajectory included reduced cognitive and physical functioning, elevated anxiety and depressive symptoms, lower beliefs in treatment control, and family relational factors. The strong correlation between anxiety and depression indicates a high-distress phenotype, with group average HADS score remaining above clinical thresholds over time. In addition, strong negative associations of depressive symptoms with physical and cognitive functioning (r ~ 0.5) suggest a distress–functional impairment cluster. Family cohesion and illness perceptions showed weaker associations with emotional symptoms, indicating partially distinct psychosocial dimensions that may also represent important independent intervention targets.

Excellent QoL: At the baseline, the excellent QoL trajectory reflected a multidimensional resilience profile characterized by strong psychological resources (coping self-efficacy, general self-efficacy, meaningfulness, mindfulness, and overall resilience) together with very low maladaptive cognitive and emotional responses (catastrophizing, self-blame, anxiety, and distress). This profile also included higher future perspective, preserved functioning across multiple domains, very low symptom burden, and supportive social contexts.

Compared with the low deteriorating trajectory, the excellent trajectory appeared to be shaped by distinct resilience mechanisms related to meaning, awareness, and behavioral engagement. While both QoL trajectories were associated with coping self-efficacy, the excellent group was further characterized by higher meaningfulness, a motivational component reflecting engagement with life challenges, and mindfulness representing greater present-moment awareness. In contrast, the low trajectory was more strongly characterized by reduced perceived manageability, reflecting diminished perceived resources and ability to cope with stressors.

The excellent profile also showed very low maladaptive cognitive responses, particularly catastrophizing and self-blame, and was distinguished primarily by low anxiety and distress, rather than by low depressive symptoms. Favorable clinical and treatment factors (e.g., luminal-type disease, endocrine therapy without chemotherapy, and absence of neoadjuvant chemotherapy) and sociodemographic characteristics (not being unemployed/housewife and not following a vegetarian diet) also contributed to this trajectory.

At month 3, excellent QoL continued to reflect low symptom burden (fatigue, pain, arm symptoms, and systemic therapy side effects) and preserved functioning across physical, role, and social domains. The profile was further characterized by higher positive affect (that reflects energetic, engaged, and enthusiastic emotions), low emotional distress (depression, anxiety, and distress thermometer), lower anxious preoccupation, higher future perspective, stronger personal control beliefs over illness, and greater social resources, including single-item perceived support, family cohesion and communication and emotional support. Interestingly, coping through physician communication was lower, suggesting that this strategy may reflect reactive help-seeking triggered by concerns rather than a resource promoting excellent QoL.

Recovering QoL: The recovering trajectory reflected a profile of complementary psychological resources, adaptive coping strategies, and preserved functioning. At baseline assessment, similarly to the excellent trajectory, the recovering trajectory was distinguished from the moderate trajectory by higher coping self-efficacy and mindfulness. In addition, optimism and the adaptive cognitive strategies of planning (thinking about the steps needed to manage a negative event) and putting events into perspective (relativizing the seriousness of an event by comparing it to other situations) emerged as robust predictors. At both time points, recovery was associated with higher positive affect, better social and sexual functioning, and lower pain. By month 3, recovery was further differentiated by lower psychological distress (anxiety and depression), together with higher self-efficacy (single-item indicator), lower helplessness/hopelessness, and greater fighting spirit, reflecting an optimistic and active coping orientation in which illness is viewed as a challenge to overcome. Greater endorsement of the coping strategy “seeing the situation as a challenge” further characterized this trajectory.

Together, these findings suggest that QoL trajectories reflect distinct configurations of vulnerability and resilience processes involving psychological, functional, social, and clinical factors. Although these mechanisms remain relevant from the baseline to month 3, the specific predictors evolve over time. QoL deterioration appears primarily driven by sustained distress, maladaptive cognitive responses, and functional impairment, whereas excellent QoL reflects active resilience processes, preserved functioning, and strong psychosocial resources. Recovery, in turn, reflects the mobilization of adaptive coping strategies and psychological resources that restore well-being over time.

Stable moderate/high depression vs. resilient: Based on clinical-site-adjusted univariable logistic regression analyses, significant differences were observed across most examined variables. However, elastic net feature selection retained a limited subset of predictors that independently distinguished the stable moderate/high HADS depression trajectory from the resilient one when the joint influence of multiple variables was considered.

At the baseline, this differentiation is driven by two opposing thematic domains: the distress axis (anxiety, depression, and distress) and complementary resilience-related resources (optimism, manageability, meaningfulness, and resilience). Stable depression also appears linked to specific cognitive styles, particularly catastrophizing and lower future perspective. In addition, arm symptoms and role functioning contribute to the differentiation between groups, with moderate-to-strong correlations suggesting that physical limitations may affect the ability to maintain daily roles. Given that approximately 70% of participants had undergone breast cancer surgery, arm symptoms may partly reflect treatment-related physical sequelae influencing daily functioning. Finally, financial impact appears to act as a relatively independent contributor to the differentiation between resilient individuals and those with moderate-to-high depression.

By month 3, during active treatment, the stable moderate/high depression trajectory remained characterized by persistent emotional distress (anxiety, distress thermometer scores, negative affect, anxious preoccupation, and lower emotional functioning), alongside lower positive affect, greater helplessness/hopelessness, and lower future perspective. This group was further distinguished by a greater symptom burden (fatigue, pain, and arm symptoms), as well as lower cognitive functioning, sexual functioning and enjoyment. Individuals in this trajectory also reported lower perceived emotional support. Finally, spiritual change contributed to group differentiation, with those experiencing moderate/higher depression more likely to report changes in spiritual beliefs or existential outlook, probably as part of their coping process.

Delayed depression occurrence vs. resilient: The comparison between the resilient and delayed depression trajectories suggests that the delayed onset of depressive symptoms is less strongly rooted in baseline psychological vulnerability. At the baseline, differences were minimal and primarily reflected physical symptoms (diarrhea and pain) and role functioning. Manageability and optimism were initially selected as predictors; however, their selection frequency dropped substantially when clinical site was forced into the model, suggesting that part of their predictive value may be related to site-level differences or contextual factors. By month 3, delayed depression was distinguished by the same physical symptom burden, together with lower emotional functioning, increased anxiety, lower sexual functioning, and greater engagement with illness-related concerns. In addition, non-university education, pre-existing mental illness, and triple-negative disease emerged as factors differentiating individuals with delayed depression. Overall, these findings suggest that delayed depression may reflect a dynamic response to accumulating treatment- and disease-related stressors, rather than pre-existing psychological risk alone.

Recovering vs. stable moderate/high depression: The contrast between the recovering and stable moderate/high depression trajectories revealed only a limited set of robust predictors once clinical site effects were taken into account. At baseline, sense of manageability emerged as the sole stable psychological factor associated with recovery. By month 3, recovery was primarily differentiated by lower anxiety, better emotional functioning, emotional support and absence of metabolic disease.

Two distinct patterns emerged regarding the role of clinical site. First, clinical site was consistently selected as a predictor of depression trajectories, but it did not significantly differentiate QoL trajectory contrasts, suggesting that depressive symptoms may be more sensitive to cross-country contextual differences than overall perceived well-being. Second, forcing clinical site into the elastic net models reduced the selection frequency of several individual-level predictors below the stability threshold. This indicates that clinical site may capture broader contextual influences associated with country-level differences, such as healthcare systems, cultural norms, social support structures, and treatment pathways, which may partly account for the variability otherwise attributed to individual factors.

The current study extends the findings of a previous analysis by Karademas et.al. (2023) [79] stemming from BOUNCE dataset, which employed a shape-based clustering approach (advanced k-means using kmlShape in R [80]) combined with a random forest classifier to identify predictors of dichotomized outcomes. By adopting a more statistically rigorous probabilistic framework, namely latent class growth analysis (LCGA) and growth mixture modeling (GMM), we moved from distance-based partitioning to model-based clustering, which explicitly accounts for the underlying data distribution and provides a formal basis for class selection through established fit indices. Furthermore, rather than collapsing trajectories into binary endpoints, a practice that can obscure clinically relevant temporal heterogeneity, we retained individual trajectory classes to capture nuanced patterns of change in QoL and depressive symptoms over time, while conducting clinically motivated binary contrasts (e.g., excellent vs. non-excellent or low deteriorating vs. remaining trajectories) to support risk stratification analyses. This strategy, combined with elastic net regularization, enabled a more stable and interpretable selection of predictors by effectively addressing multicollinearity and mitigating the risk of overfitting and unstable variable selection that can arise in non-parametric tree-based methods, particularly in settings with highly correlated predictors [68,81]. Finally, clinical site heterogeneity was explicitly addressed through secondary stability analyses in which site variables were forced into the model, allowing for the identification of robust, site-independent predictors.

The latent class trajectories identified in this study show strong conceptual alignment with the patterns reported in [79]. Both analyses consistently identified stable high-QoL/low-distress and chronic low-QoL/high-distress groups, as well as trajectories reflecting recovery or delayed response. Differences primarily relate to class composition and size. Recovering and delayed-response classes in the present study were substantially smaller (approximately one-half to one-third) than those reported by Karademas et al. (2023) [79]. In addition, we did not identify a delayed-deterioration QoL trajectory (characterized by initially moderate-to-high levels followed by decline) or an “unstable good” V-shaped trajectory attributed to transient treatment effects, although V-shaped patterns were also evident within other trajectories. In the present analysis, mean trajectory shapes cannot exhibit sharp V-shaped patterns, as they are approximated by smooth (linear or quadratic) functions.

Differences primarily reflect the tendency of shape-based clustering to distinguish and group in one class timing-related subpatterns, while the model-based approach assumes groups are defined by shared growth parameters (like specific intercepts and slopes) and accounts for within-class variability.

The kmlShape algorithm is designed to identify complex trajectory shapes [80] and preserve them in the mean trajectory. Such an example is a sharp “V-shaped” (drop-then-recovery) pattern that may better reflect short-term treatment effects. However, this approach clusters individuals based on shape similarity alone, regardless of the timing of changes (e.g., whether a decline occurs at month 3 or month 9), and is insensitive to baseline (intercept) differences as it focuses on relative movements along the path. (e.g., it can recognize that a patient dropping from 80 to 50 and back to 80 has the same V-shape as one dropping from 60 to 30 and back to 60).

In contrast, the LCGA/GMM approach is a statistical tool that accounts for uncertainty and individual-level differences within the groups. It is better suited when the timing of changes is clinically meaningful (e.g., does dropping early predict worse outcomes than dropping late?) and due to its parametric formulation, is sensitive to intercept differences (e.g., a “high baseline” group and a “low baseline” group are statistically different populations, even if they share the same slope or recovery shape). Moreover, because GMM typically fits smooth (e.g., quadratic) trajectories, very sharp V-shaped patterns may not be identified as distinct classes and can instead be treated as outliers around a stable underlying trend. Notably, in our results, the excellent QoL trajectory showed no treatment-related decline based on visual inspection of individual-level trajectories (i.e., the methodology distinguished a class that is not affected by treatment), whereas declines at treatment time points were observed across all other classes.

There is evidence that suggests that GMM may outperform alternative approaches, including k-means, in certain settings [82,83,84,85]. However, further methodological work is needed to clarify the conditions under which different longitudinal clustering methods converge or diverge. Hybrid or comparative frameworks ([82]; latrend R package) may offer promising avenues for improving the identification of clinically meaningful longitudinal phenotypes.

Regarding selected predictors, differences between our findings and those reported by Karademas et al. (2023) [79] likely reflect important methodological distinctions rather than true inconsistencies. In the previous analysis, trajectories were collapsed into dichotomous outcomes, whereas our approach retained multiple, distinct trajectory classes, allowing for example clinical factors—such as neoadjuvant chemotherapy and triple-negative disease—to emerge as relevant for specific patterns rather than for broad outcome categories. As a result, predictors associated with particular clinical courses may have been obscured in the earlier binary framework. In addition, the use of random forests in the prior study may have influenced variable importance estimates in the presence of highly correlated predictors, where importance can be distributed across correlated features or masked entirely. In contrast, the elastic net framework explicitly addresses multicollinearity, enabling more stable identification of clinical predictors whose effects are conditional on specific trajectory memberships.

While the majority of patients maintain favorable trajectories, a clinically vulnerable minority experiences persistent or deteriorating functional outcomes that require intensive, targeted intervention. Future research must prioritize the early identification of QoL and distress profiles immediately following a breast cancer diagnosis. This period serves as a critical window for proactive risk stratification and the development of personalized supportive care plans.

To improve the precision of these models, larger, well-characterized cohorts are necessary to capture the heterogeneity and temporal dynamics inherent in these high-risk subgroups. Advanced machine learning (ML) approaches, such as XGBoost or Random Survival Forests, offer a unique opportunity to integrate multidimensional factors, including clinical biomarkers, and psychosocial variables. However, researchers must employ robust techniques like Synthetic Minority Over-Sampling (SMOTE) [86] or engineered up-sampling (ENUS) [87] to handle significant data imbalance, ensuring that models do not overlook the high-risk minority.

Furthermore, the transition to clinical practice requires interpretable and calibrated prediction frameworks [88]. When paired with interpretable frameworks like SHAP for clinical transparency [89] or Conformal Prediction [90,91,92] for calibrated risk estimates accompanied by rigorous confidence intervals, ML models can support shared decision-making without the typical opacity of black-box systems, thus, fostering trust in Clinical Decision Support (CDS) tools. By identifying the key variables associated with trajectory membership, this analysis lays the foundation for clinically meaningful risk models that support shared decision-making and the efficient allocation of survivorship resources.

### Strengths and Limitations

The present study has several strengths. First, it leverages the multinational BOUNCE cohort, a prospective multicenter study conducted across four countries, which enhances the generalizability of the findings. Second, the analytical strategy combines clinically interpretable models with elastic net regularization, enabling the identification of parsimonious sets of predictors while accounting for correlations among variables and limiting overfitting. The robustness of the results was supported by stability analyses across multiple imputations and repeated model fitting, ensuring that findings were not dependent on a single imputed dataset or data partition. The elastic net specification (α = 0.5) allowed effective handling of correlated clinical predictors, while forcing clinical site into the model served as a sensitivity check to identify predictors that remained consistent across countries and clinical settings. Finally, the use of the λ = 1SE rule produced a simpler and more interpretable model with improved potential for generalization to other patient populations. In addition, the analysis focused on resilience-oriented contrasts between trajectory groups, addressing clinically meaningful questions regarding resilience, recovery, deterioration, and persistent impairment following breast cancer diagnosis.

Several limitations should be considered when interpreting the findings. By restricting the sample based on age and psychiatric history, the current study may have limited generalizability to younger or older patients and those with pre-existing mental health conditions. Furthermore, slightly higher baseline quality-of-life scores among included participants suggest a potential selection bias toward individuals with better initial well-being, which may limit generalizability to patients with poorer baseline health status. Pre-diagnosis levels of psychological variables were not available, limiting our ability to distinguish cancer-related changes from pre-existing conditions. Not all variables were assessed at the baseline and month 3 assessments, which may have constrained the temporal resolution of the analyses. Differences across clinical sites, including variations in care pathways and cultural contexts, may have contributed to heterogeneity in patient-reported outcomes despite adjustment for site effects. In addition, the small sample sizes in specific trajectory classes may have constrained the stability of the model, increasing the risk of overfitting and limiting the generalizability of predictors identified within these smaller cohorts. Moreover, because trajectory group membership was based on modal class assignment, classification uncertainty was not fully accounted for in the regression analyses; this may have introduced some misclassification and potentially attenuated associations. Finally, some analyses were cross-sectional in nature, which precludes causal inference.

## 5. Conclusions

This study indicates that, during the first 18 months following a breast cancer diagnosis, patients exhibit five distinct trajectories in terms of QoL and four distinct trajectories in terms of depression. These heterogeneous subgroups of patients followed distinct adjustment pathways over time, with differences associated with variations in symptom burden, functional scales, coping styles and perceived resources. These results provide insights into the multidimensional determinants of resilience and highlight potential intervention targets to support individualized care and address long-term QoL and depression in women with breast cancer.

In the case of QoL, findings suggest that preventing deterioration is not simply the inverse of promoting optimal functioning. Accordingly, interventions may require distinct emphases and targets, depending on whether the clinical goal is risk mitigation or the promotion of well-being. Preventing deterioration may require early identification and management of distress, perceived control, and maladaptive coping, while promoting optimal QoL may benefit from interventions that strengthen resilience resources, such as motivation, adaptive coping, and social support.

Overall, QoL and depression trajectories shared several determinants, including symptom burden, functional status, and psychosocial resources. However, clinical site emerged as a consistent predictor of depression trajectories but not QoL contrasts, suggesting that depressive symptoms may be more sensitive to cross-country contextual differences.

## Figures and Tables

**Table 1:** Baseline sociodemographic and lifestyle characteristics of the study participants. Total number of patients *N* = 538.

Variable	Mean (Range)	Variable	*n* (%)
Age, years	55.4 (40–70)	Monthly Income ^1^	
Missing	1	Low	103 (20.2%)
BMI	26 (17.3–54.1)	Middle	315 (61.6%)
Missing	8	High	93 (18.2%)
**Variable**	***n* (%)**	Missing	27
Country/Clinical site		Exercise level ^2^	
Portugal	134 (24.9%)	None	166 (33.7%)
Italy	95 (17.7%)	Low/moderate	179 (36.4%)
Finland	205 (38.1%)	Heavy	147 (29.9%)
Israel	104 (19.3%)	Missing	46
Missing	0	Diet	
Education		No diet	293 (54.6%)
Non-University	211 (39.3%)	Mediterranean/Vegetarian type	166 (30.9%)
University	326 (60.7%)	Special	78 (14.5%)
Missing	1	Missing	1
Marital status		Alcohol behavior ^3^	
Single/Engaged	53 (9.9%)	No Consumption	107 (22.1%)
Married/Common in Law	400 (74.9%)	Consumption in Moderation	331 (68.2%)
Divorced/Widowed	81 (15.2%)	Heavy Consumption	47 (9.7%)
Missing	4	Missing	53
Employment status		Smoking behavior	
Full/part-time/Self-employed	390 (72.9%)	Current smoker	72 (13.5%)
Unemployed/Housewife	47 (8.8%)	Never a smoker	359 (67.4%)
Retired	98 (18.3%)	Former smoker	102 (19.1%)
Missing	3	Missing	5

Percentages are based on non-missing data. ^1^ Low monthly income was defined as ≤1000 EUR for the moderate-income countries (Portugal and Italy) and ≤1500 EUR for the higher-income countries (Finland and Israel). High monthly income was defined as >3000 EUR (Portugal and Italy) or >3500 EUR (Finland and Israel). ^2^ Heavy exercise was defined as ≥200 min/week moderate or ≥100 min/week vigorous aerobic activity and combinations. Moderate aerobic activity includes walking, cycling and similar activities, while vigorous aerobic activity includes running, HIIT training and comparable high-intensity exercises. ^3^ Heavy alcohol consumption was defined as consuming more than 3 drinks on any day or more than 7 drinks per week.

**Table 2:** Baseline clinical and cancer characteristics of the study participants. Total number of patients *N* = 538.

Variable	*n* (%)	Variable	*n* (%)
Negative Life Events		Estrogen receptor Positivity	467 (89.6%)
None	58 (12.0%)	Missing	17
One event	239 (49.6%)	Progesterone receptor Positivity	410 (79.8%)
Two or more events	185 (38.4%)	Missing	24
Missing	56	HER2 Positivity	89 (18.2%)
Chronic diseases	191 (35.7%)	Missing	50
Missing	3	Ki67 levels ≥ 20%	293 (56.7%)
Metabolic diseases	123 (23.0%)	Missing	21
Missing	3	Subtypes ^1^	
Mental illness	62 (11.8%)	Luminal A-like	175 (34.9%)
Missing	13	Missing	37
Family history of breast cancer	183 (35.7%)	Luminal B-like (HER2−)	185 (38.3%)
Missing	25	Missing	55
Menopausal status pre		Luminal B-like (HER2+)	68 (13.8%)
Pre-/Perimenopausal	202 (38.5%)	Missing	44
Postmenopausal	322 (61.5%)	Her2-positive (non-luminal)	20 (3.8%)
Missing	14	Missing	17
HRT before diagnosis	105 (21.6%)	Triple-negative	26 (5.0%)
Missing	51	Missing	23
Cancer stage		Surgery	
I	251 (48.2%)	Lumpectomy	391 (74.6%)
II	223 (42.8%)	Mastectomy	133 (25.4%)
III	47 (9%)	Missing	14
Missing	17	Radiotherapy	424 (80.6%)
Cancer grade		Missing	12
I	91 (17.5%)	Systemic Therapy	
II	271 (52.2%)	Chemotherapy only (±anti-HER2)	78 (14.9%)
III	157 (30.3%)	Endocrine therapy only	247 (47.3%)
Missing	19	Chemo + Endocrine therapy (±anti-HER2)	197 (37.7%)
Cancer histological type		Missing	16
Ductal	408 (77.9%)	Anti-HER2 therapy	82 (15.4%)
Lobular	80 (15.3%)	Missing	6
Other	36 (6.9%)	Neoadjuvant Chemotherapy	84 (16%)
Missing	14	Missing	12

Percentages are based on non-missing data. ^1^ Luminal A-like: ER+, PR+, HER2−, low Ki67 (<20%); luminal B-like (HER2 negative): ER+, PR+/−, HER2−, high Ki67 (≥20%) or ER+, PR−, HER2−, Ki67 any; luminal B-like (HER2 positive): ER+, PR+/−, HER2+, any Ki67; Her2-positive (non-luminal): ER-, PR−, HER2+, any Ki67; and triple-negative (ER−, PR−, HER2−, any Ki67). Abbreviations: HRT: hormone replacement therapy and HER2: human epidermal growth factor receptor 2.

**Table 3:** Stability-selected predictors distinguishing the low deteriorating QoL trajectory from all other trajectory classes (reference) with descriptive penalized odds ratios.

Variable	Selection Freq (%) Penalized Site	Mean OR ^1^ Penalized Site	Selection Freq (%) Unpenalized Site
Baseline			
Performance ^2^: log-loss = 0.191, Brier score = 0.052, ROC-AUC = 0.855			
Depression HADS	100%	1.4081	100%
Diarrhea QLQ-C30	100%	1.004	100%
Emotional Functioning QLQ-C30	100%	0.9976	100%
Fatigue QLQ-C30	100%	1.0011	100%
GHS/QoL QLQ-C30	100%	0.9857	100%
Coping with Cancer CBI-B	100%	0.9623	97%
Manageability SOC	100%	0.9548	100%
Other-blame CERQ	100%	1.2173	100%
Pain QLQ-C30	100%	1.0154	100%
Neoadjuvant Chemotherapy	100%	1.4209	83%
Perceived support 1 item	100%	0.8896	100%
Triple-negative	83%	1.429	60%
Negative Life Events: Two or more (ref. No)	67%	1.0899	57%
Month 3 ^3^			
Performance ^2^: log-loss = 0.196, Brier score = 0.0537, ROC-AUC = 0.855			
Cognitive Functioning QLQ-C30	100%	0.9924	100%
Depression HADS	100%	1.8171	100%
Physical Functioning QLQ-C30	100%	0.9875	100%
Treatment Control Beliefs	100%	0.9005	100%
Anxiety HADS	90%	1.1226	87%
Neoadjuvant Chemotherapy	73%	1.1347	27%
Communication and cohesion FARE	70%	0.9455	73%

^1^ Penalized odds ratios are descriptive estimates from elastic net models at the selected λ and are not directly comparable due to differences in variable scaling. ^2^ The performance of the model when clinical site is penalized, i.e., not forced into the model. ^3^ GHS/QoL was excluded as a candidate predictor in the month-3 stability-selection analysis to avoid circularity with the trajectory outcome.

**Table 4:** Stability-selected predictors distinguishing the excellent QoL trajectory from all other trajectory classes (reference), with descriptive penalized odds ratios.

Variable	Selection Freq (%) Penalized Site	Mean OR ^1^ Penalized Site	Selection Freq (%) Unpenalized Site
Baseline			
Performance ^2^: log-loss = 0.276, Brier score = 0.0852, ROC-AUC = 0.879			
Anxiety HADS	100%	0.8796	93%
Cognitive Functioning QLQ-C30	100%	1.0059	100%
Constipation QLQ-C30	100%	0.998	100%
Emotional Functioning QLQ-C30	100%	1.0068	100%
Mental illness (ref. No)	100%	0.8947	10%
Fatigue QLQ-C30	100%	0.9955	100%
GHS/QoL QLQ-C30	100%	1.0501	100%
Mindfulness MAAS	100%	1.224	100%
Resilience CDRISC	100%	1.2044	100%
Self-blame CERQ	100%	0.8493	100%
Physical Functioning QLQ-C30	100%	1.0106	100%
Role Functioning QLQ-C30	100%	1.0029	100%
Luminal A-like	100%	1.3491	100%
Israel (ref. Portugal)	100%	1.1341	-
Endocrine only (ref. Chemo only +/−Anti−HER2)	100%	1.107	63%
Mediterranean/Vegetarian diet (ref. None)	100%	0.9513	70%
Unemployed/Housewife (ref. Full/part-time/Self-employed)	100%	0.9384	100%
Neoadjuvant Chemotherapy	100%	0.7816	100%
Perceived Support 1 item	100%	1.0287	97%
Future Perspective QLQ-BR23	97%	1.0014	97%
Meaningfulness SOC	93%	1.0026	80%
Positive Affect PANAS	93%	1.014	0%
General Self-efficacy 1 item	93%	1.0907	90%
Arm Symptoms QLQ-BR23	90%	0.9991	100%
Distress Thermometer NCCN	90%	0.9624	90%
Coping with cancer CBI-B	87%	1.0278	100%
Luminal B-like (HER2+)	83%	0.9583	63%
Catastrophizing CERQ	80%	0.9764	97%
Negative Life Events: Two or more (ref. No)	77%	0.9199	77%
Month 3 ^3^			
Performance ^2^: log-loss = 0.306, Brier score = 0.0928, ROC-AUC = 0.845			
Anxiety HADS	100%	0.744	100%
Fatigue QLQ-C30	100%	0.988	100%
Anxious Preoccupation mini-MAC	100%	0.8719	100%
Positive Affect PANAS	100%	1.3538	100%
Role Functioning QLQ-C30	100%	1.0055	100%
Systemic Therapy Side Effects QLQ-BR23	100%	0.9963	100%
Social Functioning QLQ-C30	100%	1.004	100%
Personal Control Beliefs over Illness	100%	1.0147	80%
Distress Thermometer NCCN	100%	0.9693	97%
What done to cope: Talked to the physician	100%	0.9495	97%
Future Perspective QLQ-BR23	97%	1.0027	97%
Depression HADS	93%	0.8901	80%
Negative Affect PANAS	93%	0.9418	13%
Physical Functioning QLQ-C30	93%	1.003	97%
Perceived Support 1 item	93%	1.0361	90%
Arm Symptoms QLQ-BR23	90%	0.9974	90%
Emotional Functioning QLQ-C30	83%	1.003	87%
Communication and Cohesion FARE	77%	1.0215	83%
Pain QLQ-C30	77%	0.9962	83%
Emotional Support mMOS-SS	73%	1.0395	67%
Negative Life Events: Two or more (ref. No)	70%	0.916	83%

^1^ Penalized odds ratios are descriptive estimates from elastic net models at the selected λ and are not directly comparable due to differences in variable scaling. ^2^ The performance of the model when clinical site is penalized, i.e., not forced into the model. ^3^ GHS/QoL was excluded as a candidate predictor in the month-3 stability-selection analysis to avoid circularity with the trajectory outcome.

**Table 5:** Stability-selected predictors distinguishing the recovering QoL trajectory from the moderate QoL trajectory (reference), with descriptive penalized odds ratios.

Variable	Selection Freq (%) Penalized Site	Mean OR ^1^ Penalized Site	Selection Freq (%) Unpenalized Site
Baseline			
Performance ^2^: log-loss = 0.464, Brier score = 0.147, ROC-AUC = 0.681			
Coping with Cancer CBI-B	100%	1.0568	97%
Mindfulness MAAS	100%	1.0276	93%
Optimism LOT-R	100%	1.1892	100%
Perspective CERQ	100%	1.0756	90%
Resilience CDRISC	100%	1.0604	0%
Pain QLQ-C30	100%	0.9978	100%
Positive Affect PANAS	100%	1.0919	100%
Sexual Functioning QLQ-BR23	100%	1.0033	90%
Social Functioning QLQ-C30	100%	1.0047	100%
Income Middle (ref. Low)	100%	0.7621	97%
Income High (ref. Low)	100%	1.5861	100%
Postmenopausal	93%	1.0937	17%
Planning CERQ	90%	1.0193	70%
Negative Life Events: Two or more (ref. No)	80%	0.9259	77%
Special diet (ref. None)	63%	0.9428	37%
Month 3 ^3^			
Performance ^2^: log-loss = 0.432, Brier score = 0.136, ROC-AUC = 0.763			
Helplessness/Hopelessness mini-MAC	100%	0.7512	100%
Pain QLQ-C30	100%	0.9945	100%
Positive Affect PANAS	100%	1.1766	100%
Sexual Functioning QLQ-BR23	100%	1.0065	100%
Non-Luminal (HER2+)	100%	1.3504	60%
Personal Control Beliefs over Illness	100%	1.0572	100%
Income Middle (ref. Low)	100%	0.7705	100%
Income High (ref. Low)	100%	1.6132	100%
Postmenopausal	100%	1.1308	7%
What done to cope: See it as a challenge	100%	1.1008	100%
General Self-efficacy 1 item	97%	1.0398	97%
Triple-negative	93%	0.8909	33%
Social Functioning QLQ-C30	87%	1.0019	97%
Fighting mini-MAC	80%	1.1059	73%
Depression HADS	77%	0.9431	83%
Anxiety HADS	70%	0.9368	83%
Negative Life Events: Two or more (ref. No)	70%	0.9331	53%

^1^ Penalized odds ratios are descriptive estimates from elastic net models at the selected λ and are not directly comparable due to differences in variable scaling. ^2^ The performance of the model when clinical site is penalized, i.e., not forced into the model. ^3^ GHS/QoL was excluded as a candidate predictor in the month-3 stability-selection analysis to avoid circularity with the trajectory outcome.

**Table 6:** Stability-selected predictors distinguishing the stable moderate/high depression trajectory class from the resilient class (reference), with descriptive penalized odds ratios.

Variable	Selection Freq (%) Penalized Site	Mean OR ^1^ Penalized Site	Selection Freq (%) Unpenalized Site
Baseline			
Performance ^2^: log-loss = 0.300, Brier score = 0.0892, ROC-AUC = 0.941			
Anxiety HADS	100%	1.2873	100%
Arm Symptoms QLQ-BR23	100%	1.0028	100%
Depression HADS	100%	15.3494	100%
Financial Impact QLQ-C30	100%	1.0006	100%
Future Perspective QLQ-BR23	100%	0.9978	100%
Catastrophizing CERQ	100%	1.1142	100%
Manageability SOC	100%	0.9752	100%
Meaningfulness SOC	100%	0.9872	100%
Optimism LOT-R	100%	0.9384	100%
Resilience CDRISC	100%	0.7783	63%
Role Functioning QLQ-C30	100%	0.9948	100%
Italy (ref. Portugal)	100%	1.3646	-
Finland (ref. Portugal)	100%	0.8601	-
Unemployed/Housewife (ref. Full/part-time/Self-employed)	100%	1.2159	0%
Coping with Cancer CBI-B	93%	0.9806	0%
Distress Thermometer NCCN	90%	1.0306	77%
Exercise level: Heavy (ref. No)	80%	0.937	0%
Month 3 ^3^			
Performance ^2^: log-loss = 0.370, Brier score = 0.115, ROC-AUC = 0.905			
Anxiety HADS	100%	1.8654	100%
Emotional functioning QLQ-C30	100%	0.9941	100%
Future Perspective QLQ-BR23	100%	0.9949	100%
Anxious Preoccupation mini-MAC	100%	1.3574	100%
Helplessness/Hopelessness mini-MAC	100%	1.3366	100%
Spiritual Change PTGI	100%	1.0459	73%
Emotional Support mMOS-SS	100%	0.7919	100%
Negative Affect PANAS	100%	1.5575	100%
Positive Affect PANAS	100%	0.8326	100%
Italy (ref. Portugal)	100%	1.7245	-
Finland (ref. Portugal)	100%	0.709	-
Exercise level: Heavy (ref. No)	100%	0.828	20%
Distress Thermometer NCCN	100%	1.0686	100%
Radiotherapy	100%	0.9329	0%
Fatigue QLQ-C30	97%	1.0028	97%
Pain QLQ-C30	97%	1.0027	90%
Sexual Enjoyment QLQ-BR23	93%	0.997	77%
Arm Symptoms QLQ-BR23	90%	1.0023	80%
Sexual Functioning QLQ-BR23	83%	0.9974	80%
University education	80%	0.9706	0%
What done to cope: Exercised	80%	0.9772	17%
Cognitive Functioning QLQ-C30	70%	0.9986	93%
Avoidance mini-MAC	63%	1.0353	0%

^1^ Penalized odds ratios are descriptive estimates from elastic net models at the selected λ and are not directly comparable due to differences in variable scaling. ^2^ The performance of the model when clinical site is penalized, i.e., not forced into the model. ^3^ Depression HADS was excluded as a candidate predictor in the month-3 stability-selection analysis to avoid circularity with the trajectory outcome.

**Table 7:** Stability-selected predictors distinguishing the delayed depression occurrence trajectory class from the resilient group (reference), with descriptive penalized odds ratios.

Variable	Selection Freq (%) Penalized Site	Mean OR ^1^	Selection Freq (%) Unpenalized Site
Baseline			
Performance ^2^: log-loss = 0.248, Brier score = 0.067, ROC-AUC = 0.781			
Diarrhea QLQ-C30	100%	1.0046	100%
Manageability SOC	100%	0.9796	3%
Optimism LOT-R	100%	0.9043	10%
Pain QLQ-C30	100%	1.0163	100%
Role Functioning QLQ-C30	100%	0.9987	97%
Finland (ref. Portugal)	100%	0.8797	-
Month 3 ^3^			
Performance ^2^: log-loss = 0.244, Brier score = 0.066, ROC-AUC = 0.754			
Diarrhea QLQ-C30	100%	1.0077	97%
Emotional Functioning QLQ-C30	100%	0.9885	87%
Mental illness (ref. No)	100%	1.8311	100%
Triple-negative	100%	1.8134	60%
Finland (ref. Portugal)	100%	0.5863	-
University education	100%	0.8106	93%
Unemployed/Housewife (ref. Full/part-time/Self-employed)	100%	1.3757	10%
What done to cope: Talked to the physician	100%	1.1374	87%
Income Middle (ref. Low)	97%	0.8602	40%
Anxiety HADS	93%	1.3522	90%
Sexual Functioning QLQ-BR23	87%	0.9958	73%
Exercise level: Heavy (ref. No)	83%	0.8848	0%
Pain QLQ-C30	67%	1.0017	13%

^1^ Penalized odds ratios are descriptive estimates from elastic net models at the selected λ and are not directly comparable due to differences in variable scaling. ^2^ The performance of the model when clinical site is penalized, i.e., not forced into the model. ^3^ Depression HADS was excluded as a candidate predictor in the month-3 stability-selection analysis to avoid circularity with the trajectory outcome.

**Table 8:** Stability-selected predictors distinguishing the recovering trajectory group from the stable moderate/high depression group (reference), with descriptive penalized odds ratios.

Variable	Selection Freq (%) Penalized Site	Mean OR ^1^ Penalized Site	Selection Freq (%) Unpenalized Site
Baseline			
Performance ^2^: log-loss = 0.575, Brier score = 0.1944, ROC–AUC = 0.664			
Manageability SOC	100%	1.0202	100%
Optimism LOT-R	100%	1.1608	10%
Italy (ref. Portugal)	100%	0.8259	-
Endocrine only (ref. Chemo only +/−Anti-HER2)	100%	0.8059	7%
Income High (ref. Low)	100%	1.1661	3%
Finland (ref. Portugal)	60%	1.0232	-
Month 3 ^3^			
Performance ^2^: log-loss = 0.558, Brier score = 0.1869, ROC–AUC = 0.696			
Anxiety HADS	100%	0.6592	97%
Italy (ref. Portugal)	100%	0.7177	-
Endocrine only (ref. Chemo only +/−Anti−HER2)	100%	0.7821	23%
Income High (ref. Low)	100%	1.3056	57%
Spiritual Change PTGI	90%	0.9701	40%
Special diet (ref. None)	90%	0.8446	77%
What done to cope: Talked to sb important	90%	1.0455	27%
Emotional Functioning QLQ-C30	87%	1.0028	87%
Upset by Hair Loss QLQ-BR23	80%	1.0012	17%
Metabolic diseases	77%	0.9565	73%
Finland (ref. Portugal)	77%	1.0455	-
Negative Affect PANAS	73%	0.9551	0%
Emotional Support mMOS-SS	70%	1.0249	87%

^1^ Penalized odds ratios are descriptive estimates from elastic net models at the selected λ and are not directly comparable due to differences in variable scaling. ^2^ The performance of the model when clinical site is penalized, i.e., not forced into the model. ^3^ Depression HADS was excluded as a candidate predictor in the month-3 stability-selection analysis to avoid circularity with the trajectory outcome.

**Figure A1:**
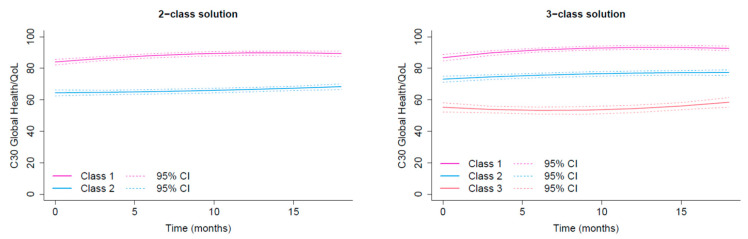

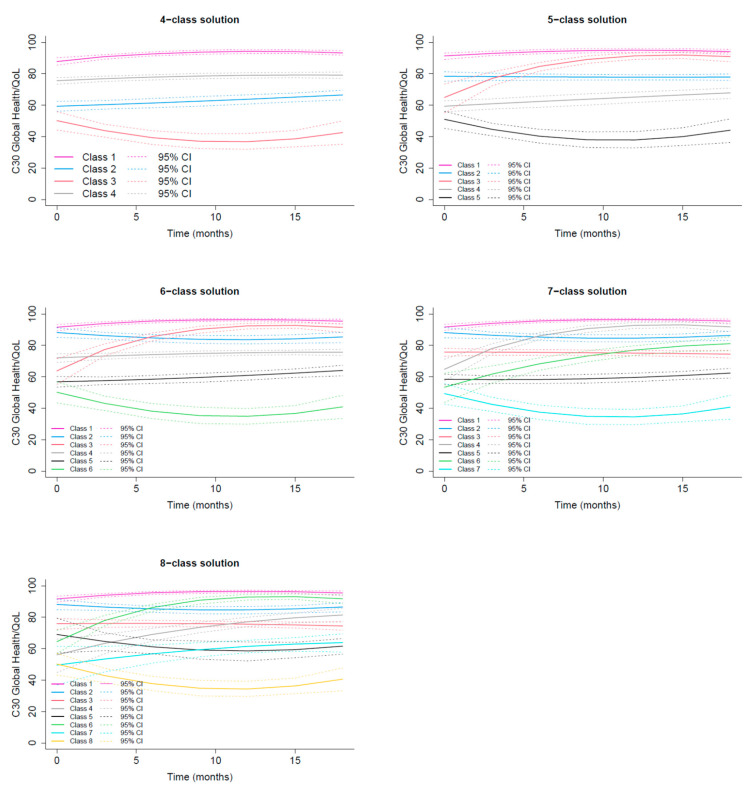
Estimated average GHS/QoL trajectories for two- to eight-class solutions, shown for the LCGA approach.

**Figure A2:**
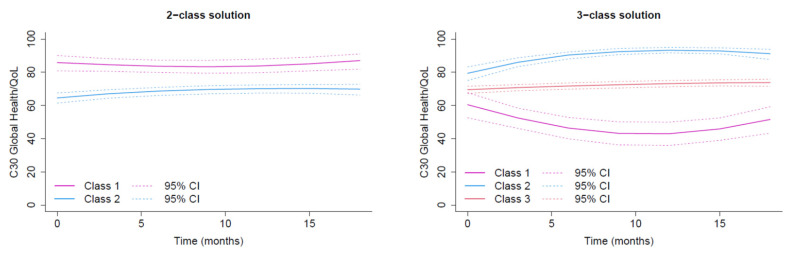

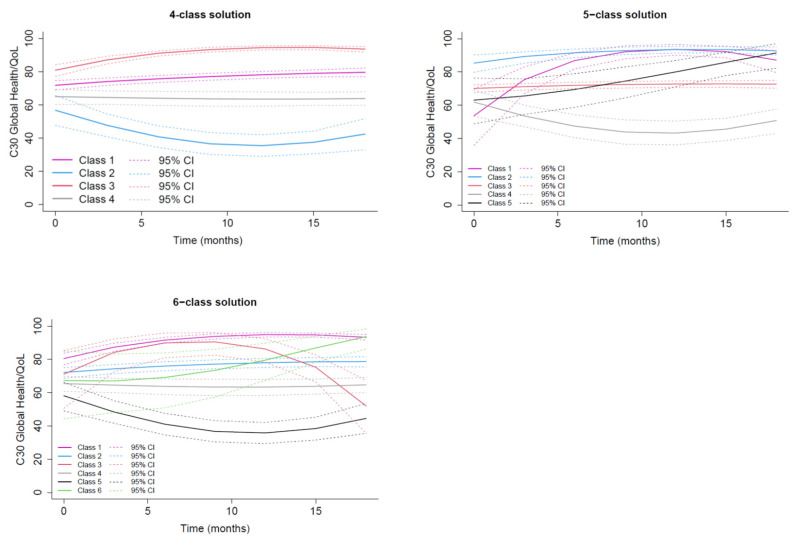
Estimated average GHS/QoL trajectories for two- to six-class solutions, shown for the GMM approach.

**Figure A3:**
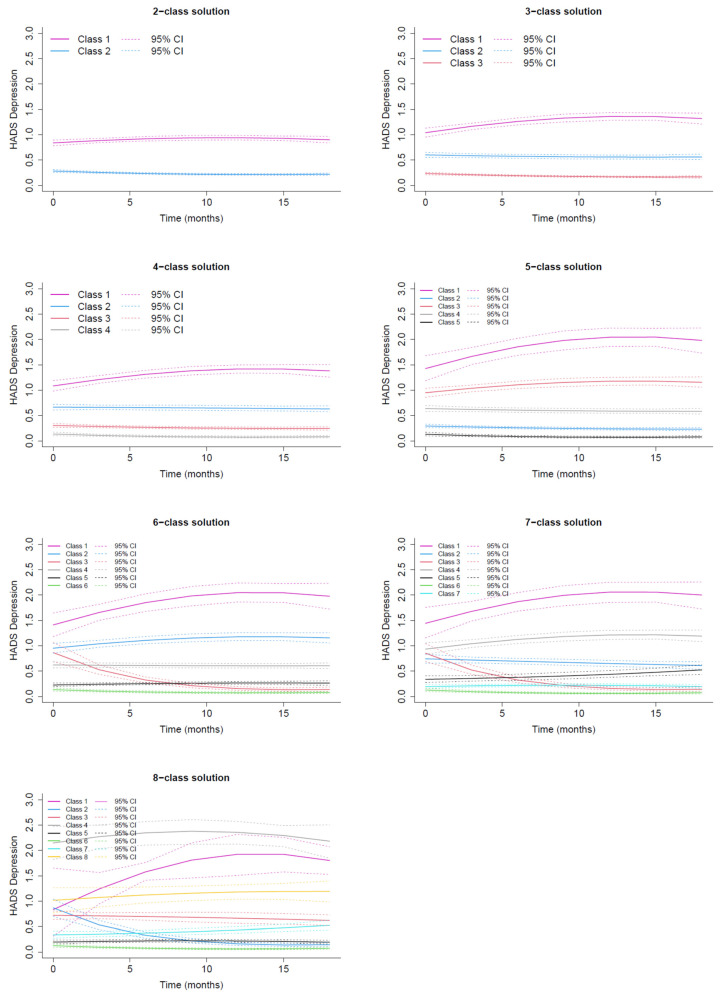
Estimated average HADS depression trajectories for two- to eight-class solutions, shown for the LCGA approach.

**Figure A4:**
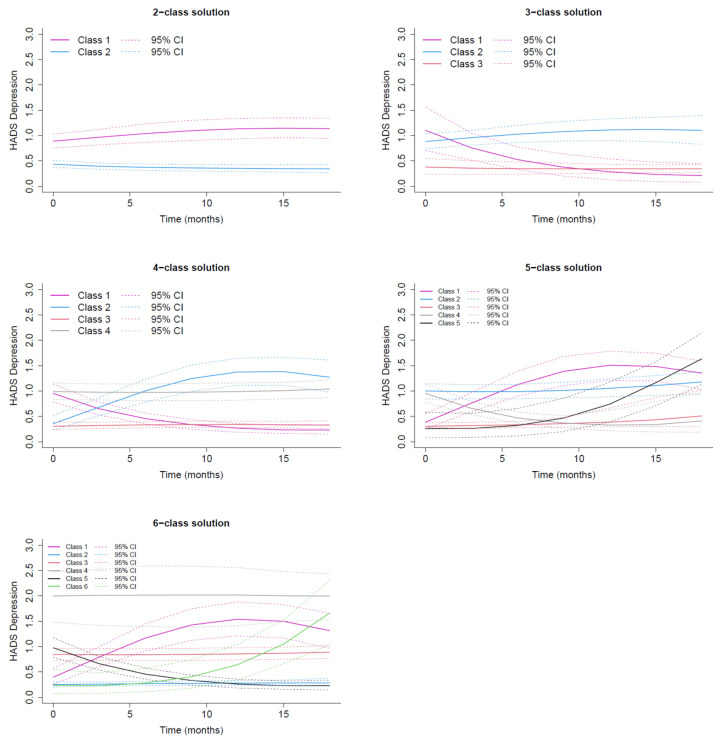
Estimated average HADS depression trajectories for two- to six-class solutions, shown for the GMM approach.

## Data Availability

The anonymized data supporting the findings of this study are available from the corresponding author upon reasonable request. Data are not publicly accessible due to ethical and privacy constraints.

## Supplementary Materials

The following supporting information can be downloaded at: https://www.mdpi.com/article/10.3390/jpm16040209/s1, Supplementary Material S1: Clinical Site-Adjusted Univariable Logistic Regression Results, Supplementary Material S2: Correlation matrices among predictors selected at baseline and month 3.

## Abbreviations

The following abbreviations are used in this manuscript:

BR23	Breast cancer-specific module of EORTC QLQ
CBI-B	Cancer Behavior Inventory
CD-RISC	Connor–Davidson Resilience Scale
EORTC QLQ-C30	European Organisation for Research and Treatment of Cancer Quality of Life Questionnaire
C30	Core 30 of EORTC QLQ
FCRI-SF	Fear of Cancer Recurrence Inventory–Short Form
GHS/QoL	Global Health Status/QoL scale of EORTC-QLQ-C30
HADS	Hospital Anxiety and Depression Scale
LOT-R	Life Orientation Test–Revised
MAAS	Mindful Attention Awareness Scale
MAC	Mental Adjustment to Cancer Scale
mMOS-SS	Modified Medical Outcomes Study Social Support Survey
PACT	Perceived Ability to Cope with Trauma Scale
PANAS	Positive and Negative Affect Schedule
PTGI-SF	Post-Traumatic Growth Inventory–Short Form
QoL	Quality of Life
SOC	Sense of Coherence Scale

## Appendix A. Selection of Link Function

Non-normality of the longitudinal outcomes (GHS/QoL, HADS Depression) is addressed by transforming the data using a non-linear link function (or latent process) from the lcmm package in R. The method simultaneously estimates the parameters of the link function and the latent process mixed model using maximum likelihood. To identify the most suitable link function, null models (i.e., with one latent class) are fit to the data, considering various non-linear link functions. Specifically, the beta cumulative distribution and I-splines differing in the number and placement of knots are explored. The I-spline associated with the lowest BIC value is selected for the final analysis. Based on Table A1, Table A2, Table A3 and Table A4, the model with a link function approximated by I-splines with six knots placed at the quantiles of the outcome distribution provides the best fit.

**Table A1:** Comparison of GHS/QoL LCGA Models with One Latent Class and Different Link Functions.

Transformation—Link Function	AIC	BIC
None	30,481.32	30,498.47
Beta cumulative distribution	29,468.93	29,494.65
I-splines with 5 equidistant knots	30,001.87	30,040.46
I-splines with 5 knots at quantiles	29,889.07	29,927.66
I-splines with 6 equidistant knots	29,997.34	30,040.21
I-splines with 6 knots at quantiles	29,412.77	29,455.64
I-splines with 7 equidistant knots	29,955.92	30,003.08
I-splines with 7 knots at quantiles	29,411	29,458.16

Note: AIC: Akaike Information Criterion; BIC: Bayesian Information Criterion.

**Table A2:** Comparison of GHS/QoL GMM Models with One Latent Class and Different Link Functions.

Transformation—Link Function	AIC	BIC
None	28,905.96	28,948.84
Beta cumulative distribution	27,780.27	27,831.73
I-splines with 5 equidistant knots	28,306.42	28,370.74
I-splines with 5 knots at quantiles	28,193.4	28,257.72
I-splines with 6 equidistant knots	28,300.39	28,369
I-splines with 6 knots at quantiles	27,722.3	27,790.9
I-splines with 7 equidistant knots	28,258.61	28,331.5
I-splines with 7 knots at quantiles	27,719.66	27,792.55

Note: AIC: Akaike Information Criterion and BIC: Bayesian Information Criterion.

**Table A3:** Comparison of HADS Depression LCGA Models with One Latent Class and Different Link Functions.

Transformation—Link Function	AIC	BIC
None	5384.34	5401.491
Beta cumulative distribution	3830.596	3856.323
I-splines with 5 equidistant knots	3459.454	3498.045
I-splines with 5 knots at quantiles	3344.796	3383.387
I-splines with 6 equidistant knots	3423.261	3466.14
I-splines with 6 knots at quantiles	3340.9	3383.778
I-splines with 7 equidistant knots	3398.965	3446.132
I-splines with 7 knots at quantiles	3339.337	3386.504

Note: AIC: Akaike Information Criterion and BIC: Bayesian Information Criterion.

**Table A4:** Comparison of HADS Depression GMM Models with One Latent Class and Different Link Functions.

Transformation—Link Function	AIC	BIC
None	2688.547	2731.426
Beta cumulative distribution	1272.653	1324.107
I-splines with 5 equidistant knots	976.3214	1040.639
I-splines with 5 knots at quantiles	882.3571	946.675
I-splines with 6 equidistant knots	955.6132	1024.219
I-splines with 6 knots at quantiles	869.0842	940.7354
I-splines with 7 equidistant knots	941.1433	1014.037
I-splines with 7 knots at quantiles	869.0842	941.9778

Note: AIC: Akaike Information Criterion and BIC: Bayesian Information Criterion.

## Appendix B. Trajectory Clustering of GHS/QoL

As shown in Table A5, fit indices suggest different optimal LCGA solutions: AIC favors the eight-class model, BIC supports the seven-class model and ICL indicates the six-class solution. Estimated trajectories for the two- to eight-class models are shown in Figure A1. All models meet the minimum class-size criterion (>5%). However, posterior probabilities in the seven- and eight-class solutions are near or below the 0.7 threshold for some classes, indicating poor class separation and reduced assignment certainty; these solutions are, therefore, not retained for further interpretation.

Visual inspection of the two- to six-class solutions reveals consistent and clinically meaningful trajectory patterns (Figure A1). Across all models, a resilient high GHS/QoL trajectory is observed, while a persistently poor and declining trajectory emerges from the four-class solution onward. An additional recovering trajectory is identified in the five- and six-class models. The remaining classes in the five- and six-class solutions exhibit largely stable trajectories that differed primarily in terms of baseline GHS/QoL levels. The six-class solution adds a class with a trajectory pattern already captured in the five-class model, providing limited additional clinical value.

Using the GMM approach, the best-fitting model based on BIC and ICL is the null model, i.e., no latent classes, while the AIC favors the five-class solution (Table A6). However, because two classes in the five-class model comprise fewer than 5% of the sample, the four-class solution is preferred. The classification quality of the four-class model is acceptable, with posterior probabilities exceeding the 0.70 threshold for all classes.

Figure A2 presents the estimated trajectories of the two- to six-class solutions with the highest loglikelihood. From the three-class solution onward, two characteristic trajectories consistently emerge: a resilient trajectory characterized by persistently high GHS/QoL scores and a persistently poor trajectory with declining scores over time. These trajectories are also identified in the LCGA results. In the four-class solution the remaining patients are split into two groups with slightly improving or steady trajectories that primarily differ in terms of their baseline GHS/QoL levels. In the three-class solution, these two groups are combined into a single trajectory. Notably, the recovering trajectory observed in the LCGA analyses does not emerge in the GMM models. Recovering classes appear in the five- and six-class GMM solutions but are too small (<5%) to be retained. Patients who comprise the recovering trajectory in the LCGA are primarily classified within the resilient trajectory in the GMM models.

Taking into account statistical fit, class separation, parsimony and clinical interpretability, the LCGA five-class solution was selected as the optimal model, while GMM results were explored but not used due to small class sizes and lack of clinically meaningful trajectories. The LCGA five-class solution captured all clinically interesting trajectory patterns while maintaining clear class differentiation and interpretability. The classification quality of the model is good, with posterior probabilities exceeding 0.8 for all classes (Table A7).

**Table A5:** Goodness-of-fit statistics for GHS/QoL LCGA model solutions and proportion of each class.

						Relative Class Size (%)							
No of Classes	Loglik	AIC	BIC	Entropy	ICL	Class1	Class2	Class3	Class4	Class5	Class6	Class7	Class8
1	−14,696	29,413	29,456	1	29,456	100	-	-	-	-	-	-	-
2	−14,170	28,368	28,428	0.857	28,481	32.16	67.84	-	-	-	-	-	-
3	−13,959	27,955	28,032	0.836	28,129	19.89	52.79	27.32	-	-	-	-	-
4	−13,908	27,861	27,955	0.809	28,098	16.36	29.55	6.32	47.77	-	-	-	-
5	−13,869	27,789	27,901	0.800	28,074	13.20	40.71	7.81	31.41	6.88	-	-	-
6	−13,836	27,732	27,860	0.795	28,058	10.22	12.27	7.06	41.64	23.42	5.39	-	-
7	−13,820	27,708	27,854	0.768	28,097	10.04	11.52	35.50	6.51	23.05	8.36	5.02	-
8	−13,815	27,706	27,868	0.726	28,175	9.85	11.71	35.13	8.55	10.41	6.51	12.45	5.39

Note: AIC: Akaike Information Criterion; BIC: Bayesian Information Criterion; and ICL: Integrated Complete Likelihood.

**Table A6:** Goodness-of-fit statistics for GHS/QoL GMM model solutions and proportion of each class.

						Relative Class Size (%)					
No of Classes	Loglik	AIC	BIC	Entropy	ICL	Class1	Class2	Class3	Class4	Class5	Class6
1	−13,845	27,722	27,791	1	27,791	100	-	-	-	-	
2	−13,841	27,722	27,808	0.504	27,993	22.68	77.32	-	-	-	-
3	−13,831	27,710	27,813	0.766	27,951	7.99	19.14	72.86	-	-	-
4	−13,825	27,706	27,826	0.713	28,040	46.28	5.58	17.47	30.67	-	-
5	−13,819	27,702	27,839	0.759	28,048	3.35	15.61	69.70	8.18	3.16	-
6	−13,816	27,704	27,858	0.731	28,118	17.47	44.61	1.12	28.81	5.95	2.04

Note: AIC: Akaike Information Criterion; BIC: Bayesian Information Criterion; and ICL: Integrated Complete Likelihood.

**Table A7:** Average of posterior probabilities in each class for GHS/QoL LCGA five-class solution.

Assigned Class	Class 1 Excellent GHS/QoL	Class 2 Good GHS/QoL	Class 3 Recovering GHS/QoL	Class 4 Moderate GHS/QoL	Class 5 Low Deteriorating GHS/QoL
1	0.9222	0.0378	0.0400	0.0000	0.0000
2	0.0093	0.8547	0.0338	0.1022	0.0000
3	0.0657	0.1222	0.8002	0.0118	0.0000
4	0.0000	0.0944	0.0047	0.8636	0.0373
5	0.0000	0.0000	0.0000	0.0808	0.9192

## Appendix C. Trajectory Clustering of HADS Depression

The best-fitting LCGA model according to AIC and BIC is the eight-class solution, whereas ICL favors the six-class model (Table A8). However, in the five- to eight-class solutions, the smallest class comprises less than 5% of the sample, making these models unsuitable for further analysis.

In the two-, three- and four-class solutions, the classes exhibit very similar trajectory shapes (Figure A3). Their average trajectories remain largely steady over the 18-month period, with the exception of the class with the highest baseline depression scores, which shows a modest increase. Consequently, most trajectories are nearly parallel, differing primarily in terms of baseline depression levels, all of which are below the clinically meaningful threshold of 1. From a clinical perspective, reassigning patients with depression scores below this threshold provides little added value.

Using the GMM approach, the best-fitting model according to BIC and ICL is the null model, i.e., no latent classes, while the AIC indicates the six-class solution (Table A9). However, in the six- and five-class solutions, the size of the smallest class falls below 5% of the sample, making the four-class solution the preferred choice. The classification quality of the four-class model is acceptable, with posterior probabilities exceeding the 0.70 threshold for all classes.

Visual inspection of the four-class solution (Figure A4) reveals clinically meaningful trajectory patterns that were also present in solutions with a higher number of classes: two stable trajectories, one at low depression scores and one around a score of 1, as well as a low-worsening trajectory and a high-improving trajectory. Remarkably, the improving trajectory is also identified in the LCGA solutions with six or more classes.

Considering both statistical fit and clinical relevance, the four-class GMM solution was selected as optimal, while LCGA results were explored but not used due to less interpretable class separation and similar trajectory shapes across classes. This model encompassed all clinically important trajectory patterns while keeping class membership easily interpretable. The classification quality of the model is acceptable, with posterior probabilities exceeding 0.75 for all classes (Table A10).

**Table A8:** Goodness-of-fit statistics for HADS Depression LCGA model solutions and proportion of each class.

No of Classes	Loglik	AIC	BIC	Entropy	ICL	%Class1	%Class2	%Class3	%Class4	%Class5	%Class6	%Class7	%Class8
1	−1660	3341	3384	1	3384	100	-	-	-	-	-	-	-
2	−861	1750	1810	0.899	1848	47.21	52.79	-	-	-	-	-	-
3	−631	1298	1375	0.873	1450	19.70	38.48	41.82	-	-	-	-	-
4	−553	1149	1243	0.845	1359	16.54	33.64	37.92	11.90	-	-	-	-
5	−497	1047	1158	0.851	1287	3.53	37.17	17.47	30.67	11.15	-	-	-
6	−455	971	1099	0.843	1251	3.53	17.47	6.88	28.81	30.67	12.64	-	-
7	−434	936	1082	0.798	1293	3.53	22.12	7.62	15.99	16.36	10.78	23.61	-
8	−412	900	1063	0.809	1277	2.60	7.62	23.05	1.12	23.79	10.59	15.61	15.61

Note: AIC: Akaike Information Criterion; BIC: Bayesian Information Criterion; and ICL: Integrated Complete Likelihood.

**Table A9:** Goodness-of-fit statistics for HADS Depression GMM model solutions and proportion of each class.

						Relative Class Size (%)					
No of Classes	Loglik	AIC	BIC	Entropy	ICL	%Class1	%Class2	%Class3	%Class4	%Class5	%Class6
1	−420	872	941	1	941	100	-	-	-	-	-
2	−410	860	946	0.586	1101	21.93	78.07	-	-	-	-
3	−403	854	957	0.657	1159	2.60	23.79	73.61	-	-	-
4	−391	838	958	0.681	1196	10.04	5.02	59.67	25.28	-	-
5	−383	829	966	0.732	1199	4.28	25.09	60.04	9.85	0.74	-
6	−373	819	973	0.772	1193	3.72	51.67	33.83	1.67	8.36	0.74

Note: AIC: Akaike Information Criterion; BIC: Bayesian Information Criterion; and ICL: Integrated Complete Likelihood.

**Table A10:** Average of posterior probabilities in each class for HADS Depression GMM four-class solution.

Assigned Class	Class 1 Recovering	Class 2 Delayed Occurrence	Class 3 Resilient	Class 4 Stable Moderate/High
1	0.7735	0.0000	0.1608	0.0657
2	0.0000	0.7899	0.1206	0.0895
3	0.0375	0.0090	0.8620	0.0914
4	0.0353	0.0348	0.1760	0.7540

## Appendix D. Maximum Likelihood Estimates of the Selected Models

**Table A11:** Maximum Likelihood Estimates for the selected GHS/QoL five-class model. The Table presents estimated parameters, standard errors (SE), the Wald test statistics and corresponding *p*-value. The Wald test assesses whether each parameter differs significantly from zero. Classes 1, 2, 3, 4 and 5 correspond to the Excellent, Good, Recovering, Moderate and Low deteriorating GHS/QoL trajectory latent classes, respectively.

Fixed Effects in the Class-Membership Model: (the Class of Reference Is the Last Class)				
	**Coefficient**	**SE**	**Wald**	***p*-Value**
intercept class 1	0.55548	0.28801	1.929	0.05378
intercept class 2	1.65476	0.30204	5.479	0.00000
intercept class 3	0.10195	0.35111	0.290	0.77153
intercept class 4	1.44951	0.23372	6.202	0.00000
**Fixed effects in the longitudinal model:**				
	coefficient	SE	Wald	*p*-value
intercept class1 (not estimated)	0			
intercept class 2	−1.34743	0.18936	−7.116	0.00000
intercept class 3	−2.39865	0.28857	−8.312	0.00000
intercept class 4	−2.74885	0.18200	−15.103	0.00000
intercept class 5	−3.26800	0.21464	−15.226	0.00000
linear slope class 1	0.09053	0.03134	2.889	0.00386
linear slope class 2	−0.00849	0.02040	−0.416	0.67729
linear slope class 3	0.34277	0.06492	5.280	0.00000
linear slope class 4	0.03283	0.02206	1.488	0.13668
linear slope class 5	−0.13893	0.04217	−3.295	0.00099
quadratic slope class 1	−0.00378	0.00164	−2.305	0.02117
quadratic slope class 2	0.00033	0.00101	0.329	0.74253
quadratic slope class 3	−0.01185	0.00300	−3.945	0.00008
quadratic slope class 4	−0.00008	0.00116	−0.070	0.94426
quadratic slope class 5	0.00651	0.00222	2.934	0.00335
**Parameters of the link function:**				
	coefficient	SE	Wald	*p*-value
I-splines 1	−5.95455	0.21304	−27.950	0.00000
I-splines 2	1.06674	0.09931	10.742	0.00000
I-splines 3	0.91341	0.09308	9.813	0.00000
I-splines 4	1.42070	0.03349	42.424	0.00000
I-splines 5	0.86157	0.02925	29.451	0.00000
I-splines 6	−1.17587	0.02114	−55.617	0.00000
I-splines 7	0.00011	0.03728	0.003	0.99767
I-splines 8	−0.95530	0.02128	−44.889	0.00000

**Table A12:** Maximum Likelihood Estimates for the selected HADS Depression four-class model. The Table presents estimated parameters, standard errors (SE), the Wald test statistics and corresponding *p* value. The Wald test assesses whether each parameter differs significantly from zero. Classes 1, 2, 3 and 4 correspond to the Recovering, Delayed Occurrence, Resilient and Stable/High Depression trajectory latent classes, respectively.

Fixed Effects in The Class-Membership Model: (the Class of Reference Is the Last Class)				
	**Coefficient**	**SE**	**Wald**	***p*-Value**
intercept class 1	−0.85515	0.34940	−2.448	0.01438
intercept class 2	−1.56106	0.40570	−3.848	0.00012
intercept class 3	0.81867	0.29328	2.791	0.00525
**Fixed effects in the longitudinal model:**				
	coefficient	SE	Wald	*p*-value
intercept class1 (not estimated)	0			
intercept class 2	−2.12840	0.43187	−4.928	0.00000
intercept class 3	−2.40407	0.29120	−8.256	0.00000
intercept class 4	0.11938	0.33984	0.351	0.72537
linear slope class 1	−0.35787	0.05419	−6.605	0.00000
linear slope class 2	0.47814	0.07396	6.465	0.00000
linear slope class 3	0.01588	0.02306	0.689	0.49109
linear slope class 4	−0.02495	0.03805	−0.656	0.51200
quadratic slope class 1	0.01082	0.00267	4.052	0.00005
quadratic slope class 2	−0.01737	0.00388	−4.478	0.00001
quadratic slope class 3	−0.00075	0.00125	−0.604	0.54560
quadratic slope class 4	0.00171	0.00216	0.792	0.42858
**Variance-covariance matrix of the random-effects:**				
	intercept	linear slope	quadratic slope	
intercept	0.89100			
linear slope	0.03042	0.00566		
quadratic slope	−0.00159	−0.00025	1 × 10^−5^	
**Parameters of the link function:**				
	coefficient	SE	Wald	*p*-value
I-splines 1	−4.61362	0.28054	−16.446	0.00000
I-splines 2	0.95180	0.02099	45.347	0.00000
I-splines 3	0.79402	0.03608	22.006	0.00000
I-splines 4	1.17562	0.02950	39.851	0.00000
I-splines 5	0.93434	0.03303	28.292	0.00000
I-splines 6	1.61728	0.04373	36.986	0.00000
I-splines 7	1.24502	0.11248	11.069	0.00000
I-splines 8	1.15337	0.13638	8.457	0.00000

## Appendix E. Scale Scores by Trajectory Group

**Table A13:** Selected psychological scale scores at baseline (M0) and month 3 (M3) by GHS/QoL trajectory group (*N* = 538).

Questionnaire (Range)	Scale	Excellent (n = 71)	Good (n = 219)	Recovering (n = 42)	Moderate (n = 169)	Low Deteriorating (n = 37)
		**Mean (SD)**				
HADS	Depression—M0	0.24 (0.32)	0.43 (0.37)	0.53 (0.47)	0.67 (0.46)	1.03 (0.70)
(0–3)	Depression—M3	0.20 (0.26)	0.46 (0.38)	0.48 (0.44)	0.81 (0.52)	1.42 (0.78)
	Anxiety—M0	0.56 (0.42)	0.85 (0.46)	0.93 (0.57)	1.15 (0.56)	1.40 (0.71)
	Anxiety—M3	0.37 (0.29)	0.66 (0.38)	0.63 (0.43)	0.92 (0.51)	1.34 (0.69)
EORTC-QLQ C30	GHS/QoL—M0	92.37 (8.99)	80.67 (11.86)	67.26 (16.71)	60.70 (16.09)	50.90 (21.85)
(0–100)	GHS/QoL—M3	92.30 (8.93)	74.14 (14.26)	67.07 (22.36)	54.35 (16.36)	42.93 (21.96)
	Emotional Functioning—M0	87.40 (13.12)	78.04 (15.59)	73.02 (22.60)	65.24 (20.35)	53.53 (26.11)
	Emotional Functioning—M3	89.68 (13.06)	79.44 (16.75)	79.47 (17.29)	69.51 (19.96)	52.70 (25.35)
	Physical Functioning—M0	94.84 (7.41)	88.55 (11.16)	87.62 (15.97)	83.14 (16.15)	74.41 (22.85)
	Physical Functioning—M3	91.52 (12.88)	83.02 (13.26)	78.98 (17.97)	72.37 (19.23)	53.33 (23.61)
	Role Functioning—M0	94.05 (12.38)	85.86 (17.75)	80.56 (24.11)	72.22 (25.97)	66.22 (31.79)
	Role Functioning—M3	95.52 (11.45)	81.13 (21.29)	75.61 (26.64)	65.29 (26.82)	49.02 (28.11)
	Cognitive Functioning—M0	94.84 (10.00)	87.29 (15.30)	85.32 (13.38)	80.16 (20.47)	69.82 (31.14)
	Cognitive Functioning—M3	91.54 (14.77)	82.31 (16.99)	81.30 (22.73)	73.48 (22.52)	50.98 (28.11)
	Social Functioning—M0	93.96 (11.78)	87.77 (16.95)	86.18 (21.38)	73.27 (24.55)	63.06 (26.39)
	Social Functioning—M3	95.02 (10.06)	82.31 (20.72)	80.89 (21.59)	65.93 (26.21)	50.49 (30.01)
	Fatigue—M0	13.77 (14.36)	23.08 (16.99)	26.19 (21.37)	33.99 (21.95)	47.75 (29.03)
	Fatigue—M3	17.74 (16.76)	34.80 (20.01)	40.92 (28.38)	48.66 (22.52)	66.01 (26.79)
	Pain—M0	6.10 (11.00)	12.44 (16.16)	13.89 (17.62)	22.19 (20.63)	40.54 (31.80)
	Pain—M3	6.97 (11.66)	17.07 (18.35)	18.29 (23.22)	33.23 (25.76)	47.55 (29.34)
	Diarrhea—M0	1.88 (7.74)	3.50 (10.24)	5.56 (12.57)	8.38 (18.91)	17.12 (27.91)
	Diarrhea—M3	4.04 (12.42)	8.49 (17.51)	9.76 (18.62)	12.87 (22.49)	20.59 (30.72)
	Constipation—M0	4.23 (11.17)	11.11 (21.73)	11.11 (22.89)	12.57 (24.98)	22.52 (33.38)
	Constipation—M3	9.95 (18.36)	16.19 (25.24)	20.33 (30.62)	22.36 (29.01)	26.47 (30.46)
EORTC-QLQ BR23	Arm Symptoms—M0	6.67 (12.46)	11.19 (14.92)	15.61 (19.33)	16.24 (17.74)	21.30 (19.04)
(0–100)	Arm Symptoms—M3	5.23 (9.30)	12.26 (15.37)	15.61 (22.63)	15.18 (17.90)	27.61 (25.17)
	Systemic Therapy Side Effects—M0	5.97 (8.00)	10.17 (9.39)	11.54 (12.59)	14.64 (15.94)	24.36 (19.89)
	Systemic Therapy Side Effects—M3	14.39 (13.68)	23.48 (15.63)	28.13 (19.87)	34.66 (18.82)	47.18 (19.83)
	Future Perspective—M0	66.67 (28.17)	54.99 (25.60)	46.34 (32.38)	41.77 (29.74)	36.19 (30.65)
	Future Perspective—M3	75.38 (19.79)	60.53 (23.16)	63.49 (27.36)	49.48 (26.90)	41.41 (31.21)
	Sexual Functioning—M0	31.57 (23.04)	28.02 (24.33)	31.25 (30.00)	20.14 (18.93)	18.75 (20.63)
	Sexual Functioning—M3	29.10 (24.13)	23.77 (21.94)	28.63 (25.06)	16.99 (17.30)	11.11 (16.49)
PANAS (1–5)	Positive Affect—M0	3.83 (0.60)	3.52 (0.65)	3.72 (0.73)	3.30 (0.79)	3.24 (0.77)
	Positive Affect—M3	3.91 (0.67)	3.33 (0.69)	3.60 (0.79)	3.08 (0.78)	2.84 (0.91)
	Negative Affect—M0	1.45 (0.59)	1.72 (0.67)	2.01 (0.78)	2.13 (0.80)	2.49 (0.89)
	Negative Affect—M3	1.28 (0.40)	1.59 (0.62)	1.65 (0.66)	1.89 (0.79)	2.35 (1.06)
NCCN (0–10)	Distress Thermometer—M0	1.97 (2.25)	3.57 (2.56)	3.73 (3.05)	4.85 (2.46)	5.68 (2.81)
	Distress Thermometer—M3	1.23 (2.12)	2.61 (2.20)	3.08 (2.70)	4.08 (2.63)	5.30 (3.23)
Single item (0–10)	Perceived Support—M0	9.49 (0.96)	9.05 (1.41)	9.32 (1.26)	8.82 (1.50)	7.81 (2.22)
	Perceived Support—M3	9.21 (1.23)	8.39 (1.83)	8.97 (1.48)	8.36 (1.62)	7.45 (2.94)
	General Self-Efficacy—M0	9.10 (0.85)	8.36 (1.37)	8.57 (1.22)	7.60 (1.60)	7.16 (2.14)
	General Self-Efficacy—M3	8.90 (1.33)	8.31 (1.24)	8.64 (1.23)	7.63 (1.67)	6.76 (2.60)
LOT-R (0–4)	Optimism—M0	3.04 (0.65)	2.84 (0.61)	2.93 (0.59)	2.55 (0.66)	2.30 (0.81)
CBI-B (1–9)	Coping with Cancer—M0	7.96 (1.05)	7.42 (0.91)	7.47 (0.94)	6.87 (1.16)	6.34 (1.30)
SOC (4–28)	Manageability—M0	21.92 (3.42)	20.62 (3.86)	20.24 (4.44)	19.19 (4.12)	16.00 (5.65)
	Meaningfulness—M0	24.54 (3.19)	23.13 (3.35)	23.05 (4.54)	22.35 (3.72)	21.24 (4.74)
MAAS (1–6)	Mindfulness—M0	4.73 (0.63)	4.40 (0.66)	4.51 (0.69)	4.20 (0.75)	4.02 (0.76)
CD-RISC (0–4)	Resilience—M0	3.22 (0.60)	2.86 (0.61)	3.02 (0.63)	2.68 (0.65)	2.53 (0.67)
CERQ (1–5)	Self-blame—M0	1.73 (0.72)	2.02 (0.81)	2.15 (0.88)	2.05 (0.76)	2.12 (0.92)
	Other-blame—M0	1.35 (0.58)	1.40 (0.54)	1.54 (0.58)	1.52 (0.64)	1.94 (0.92)
	Catastrophizing—M0	1.56 (0.66)	1.82 (0.74)	1.98 (0.91)	2.27 (0.89)	2.36 (0.95)
	Perspective—M0	3.61 (0.95)	3.41 (0.94)	3.55 (1.00)	3.12 (0.97)	2.96 (1.06)
	Planning—M0	3.47 (1.13)	3.40 (0.99)	3.75 (0.88)	3.29 (1.00)	3.41 (0.85)
FARE (1–7)	Communication and Cohesion—M3	6.56 (0.72)	6.08 (1.02)	6.27 (0.96)	6.03 (1.00)	5.26 (1.71)
mMOS-SS (1–5)	Emotional Support—M3	4.44 (0.68)	4.10 (0.82)	4.26 (0.80)	3.91 (0.82)	3.78 (0.97)
mini-MAC (1–4)	Helplessness/Hopelessness—M3	1.19 (0.32)	1.34 (0.35)	1.25 (0.31)	1.57 (0.49)	1.84 (0.70)
	Fighting Spirit—M3	3.20 (0.64)	3.15 (0.50)	3.36 (0.35)	3.10 (0.49)	3.19 (0.46)
	Anxious Preoccupation—M3	1.76 (0.50)	2.03 (0.51)	1.99 (0.44)	2.31 (0.56)	2.54 (0.60)
B-IPQ Single item	Personal Control—M3	7.41 (2.79)	6.09 (2.70)	7.22 (2.35)	5.30 (2.75)	4.62 (2.92)
(0–10)	Treatment Control—M3	9.26 (1.24)	9.06 (1.18)	9.34 (1.13)	8.62 (1.74)	7.24 (2.72)
Single item (1–5)	See it as a challenge—M3	3.20 (1.44)	3.37 (1.24)	3.90 (1.14)	3.19 (1.26)	3.26 (1.52)
	Talked to the physician—M3	1.87 (1.16)	2.35 (1.25)	2.64 (1.46)	2.68 (1.30)	2.76 (1.39)

**Table A14:** Selected psychological scale scores at baseline (M0) and month 3 (M3) by HADS depression trajectory group (*n* = 538).

Questionnaire (Range)	Scale	Resilient (n = 321)	Recovering (n = 54)	Delayed Occurrence (n = 27)	Stable Moderate/High (n = 136)
		**Mean (SD)**			
HADS (0–3)	Depression—M0	0.27 (0.24)	0.92 (0.37)	0.28 (0.25)	1.04 (0.45)
	Depression—M3	0.31 (0.27)	0.83 (0.60)	0.71 (0.49)	1.16 (0.53)
	Anxiety—M0	0.74 (0.45)	1.27 (0.54)	0.75 (0.39)	1.37 (0.55)
	Anxiety—M3	0.54 (0.36)	0.83 (0.47)	0.84 (0.39)	1.18 (0.53)
EORTC-QLQ C30	GHS/QoL—M0	79.15 (15.61)	59.72 (19.07)	68.52 (21.10)	64.03 (19.45)
(0–100)	GHS/QoL—M3	73.53 (18.65)	59.97 (22.36)	64.39 (13.16)	57.82 (21.79)
	Emotional Functioning—M0	79.75 (15.91)	63.99 (22.18)	70.88 (23.14)	61.76 (22.32)
	Emotional Functioning—M3	83.29 (14.27)	74.02 (20.86)	69.08 (17.21)	60.41 (22.78)
	Role Functioning—M0	86.52 (18.06)	74.07 (27.98)	69.75 (31.02)	72.47 (26.49)
	Role Functioning—M3	81.18 (22.25)	62.75 (30.66)	68.12 (26.07)	68.49 (28.70)
	Cognitive Functioning—M0	88.89 (13.86)	79.32 (19.94)	85.80 (18.89)	76.67 (24.87)
	Cognitive Functioning—M3	83.17 (17.80)	77.00 (24.26)	72.46 (25.43)	69.36 (25.99)
	Fatigue—M0	21.95 (17.41)	34.57 (26.05)	31.69 (27.16)	35.87 (23.19)
	Fatigue—M3	33.30 (21.54)	48.80 (29.07)	41.06 (26.68)	50.04 (25.12)
	Pain—M0	11.76 (16.46)	19.14 (22.76)	34.57 (28.09)	23.90 (22.10)
	Pain—M3	16.99 (19.79)	27.33 (30.45)	32.61 (24.35)	33.46 (26.39)
	Financial Impact—M0	7.92 (18.47)	14.20 (23.88)	22.22 (32.03)	19.01 (27.78)
	Financial Impact—M3	10.42 (19.62)	19.33 (27.84)	23.19 (30.87)	20.26 (30.92)
	Diarrhea—M0	3.75 (10.87)	6.17 (17.22)	14.81 (26.69)	9.14 (19.71)
	Diarrhea—M3	7.98 (16.37)	15.33 (27.11)	21.74 (27.72)	11.37 (22.63)
EORTC-QLQ BR23	Arm Symptoms—M0	10.00 (14.02)	16.05 (18.53)	20.09 (20.37)	18.35 (18.92)
(0–100)	Arm Symptoms—M3	10.50 (14.30)	12.18 (19.83)	18.84 (20.22)	20.12 (21.23)
	Future Perspective—M0	59.25 (24.88)	33.33 (29.96)	49.33 (34.85)	36.59 (30.39)
	Future Perspective—M3	66.34 (20.96)	49.36 (30.60)	55.07 (21.58)	42.24 (28.89)
	Sexual Functioning—M0	29.11 (23.07)	22.12 (26.14)	25.00 (26.10)	18.65 (20.18)
	Sexual Functioning—M3	25.61 (22.58)	16.32 (19.60)	13.49 (18.72)	16.40 (17.38)
	Sexual Enjoyment—M0	42.65 (34.90)	31.29 (36.90)	36.67 (38.84)	25.64 (31.24)
	Sexual Enjoyment—M3	37.85 (35.61)	20.93 (28.19)	22.81 (35.23)	19.94 (27.80)
PANAS (1–5)	Positive Affect—M0	3.60 (0.64)	3.33 (0.87)	3.60 (0.82)	3.26 (0.78)
	Positive Affect—M3	3.47 (0.68)	3.13 (0.98)	3.20 (0.70)	3.03 (0.87)
	Negative Affect—M0	1.62 (0.63)	2.19 (0.81)	1.87 (0.68)	2.41 (0.81)
	Negative Affect—M3	1.40 (0.47)	1.82 (0.78)	1.78 (0.72)	2.33 (0.83)
NCCN (0–10)	Distress Thermometer—M0	2.98 (2.45)	5.62 (2.24)	3.85 (2.76)	5.62 (2.41)
	Distress Thermometer—M3	2.10 (2.07)	4.06 (2.99)	3.09 (2.52)	5.08 (2.59)
LOT-R (0–4)	Optimism—M0	2.96 (0.59)	2.71 (0.59)	2.46 (0.60)	2.32 (0.68)
SOC (4–28)	Manageability—M0	21.19 (3.72)	19.98 (3.98)	17.96 (4.84)	17.58 (4.46)
	Meaningfulness—M0	23.63 (3.21)	22.63 (4.29)	21.81 (5.39)	21.62 (3.88)
CD-RISC (0–4)	Resilience—M0	3.06 (0.52)	2.67 (0.69)	2.74 (0.55)	2.42 (0.72)
CERQ (1–5)	Catastrophizing—M0	1.72 (0.69)	2.23 (0.93)	2.06 (0.90)	2.47 (0.91)
mMOS-SS (1–5)	Emotional Support—M3	4.24 (0.77)	4.04 (0.86)	3.91 (0.94)	3.73 (0.83)
mini-MAC (1–4)	Helplessness/Hopelessness—M3	1.27 (0.33)	1.49 (0.49)	1.47 (0.57)	1.71 (0.52)
	Anxious Preoccupation—M3	1.91 (0.47)	2.29 (0.49)	2.12 (0.50)	2.53 (0.57)
Single item (1–5)	Talked to the physician—M3	2.24 (1.27)	2.83 (1.28)	3.26 (1.05)	2.62 (1.35)
PTGI (0–5)	Spiritual Change—M3	1.47 (1.47)	1.41 (1.60)	1.83 (1.61)	2.11 (1.70)
