# Supplementary material for: In Silico Psycho-Oncology: Understanding Resilience Pathways in Breast Cancer—Determinants of Longitudinal Depression and Quality-of-Life Trajectories
Source: J Pers Med. 2026 Apr 7;16(4):209. doi: 10.3390/jpm16040209 (PMC13117978; doi:10.3390/jpm16040209)
Supplement: Supplementary file 1 [file jpm-16-00209-s001.zip › Supplementary Material S1 Clinical Site–Adjusted Univariable Logistic Regression Results.pdf]

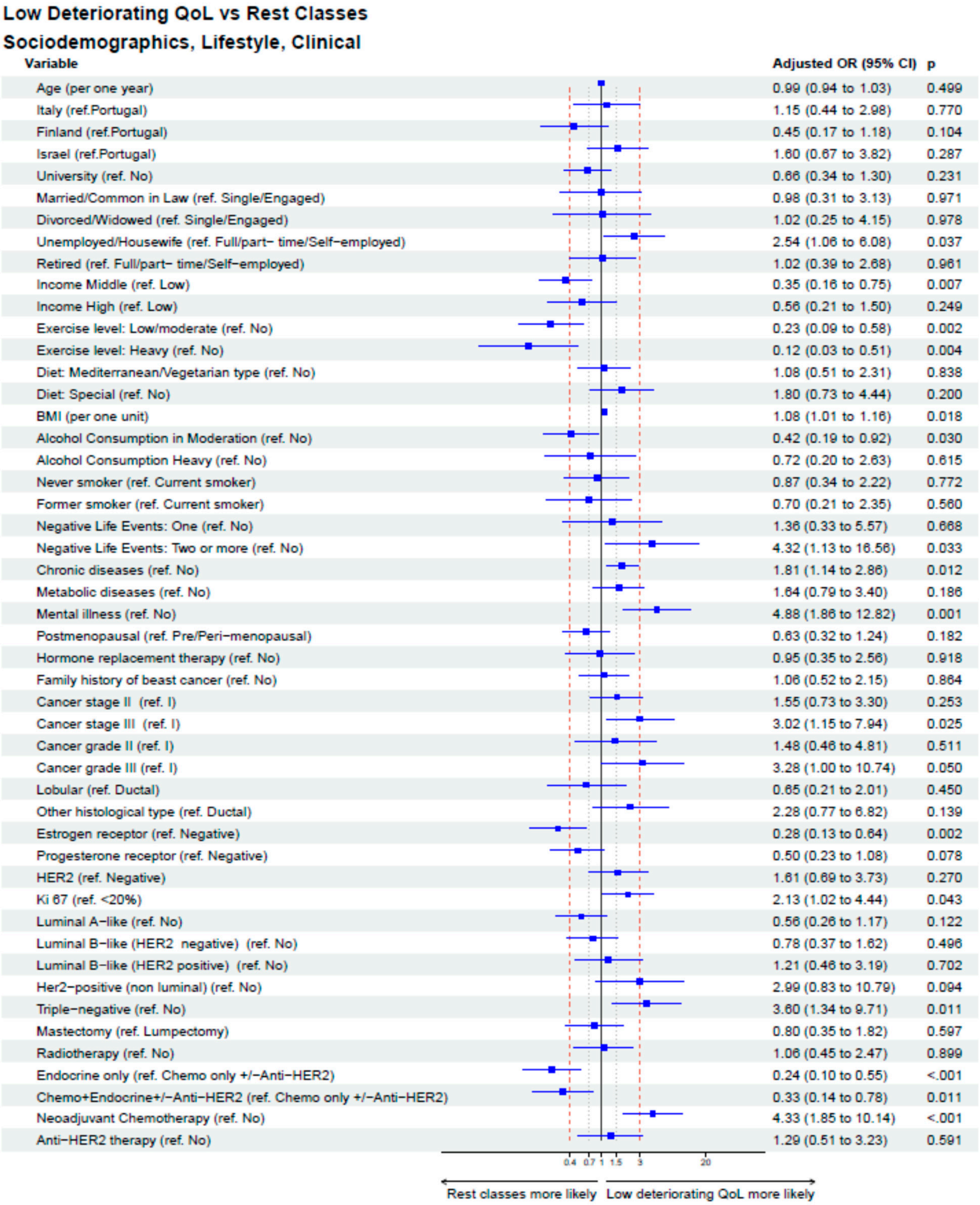

Figure S1. Odds ratios (95% CI), adjusted for clinical site, for sociodemographic, lifestyle, clinical and cancer-related factors associated with the Low deteriorating QoL trajectory class versus other classes at baseline. Values >1 indicate higher likelihood of Low deteriorating QoL; values <1 indicate higher likelihood of other classes.

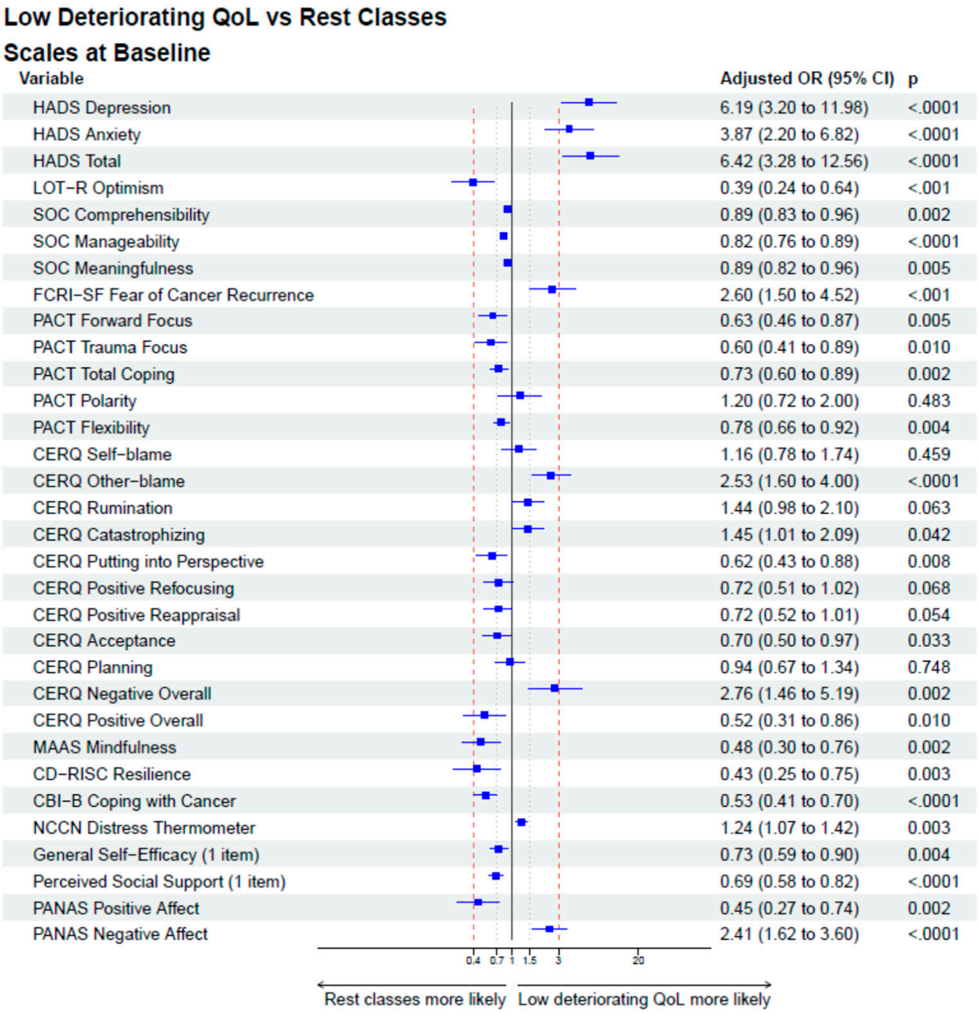

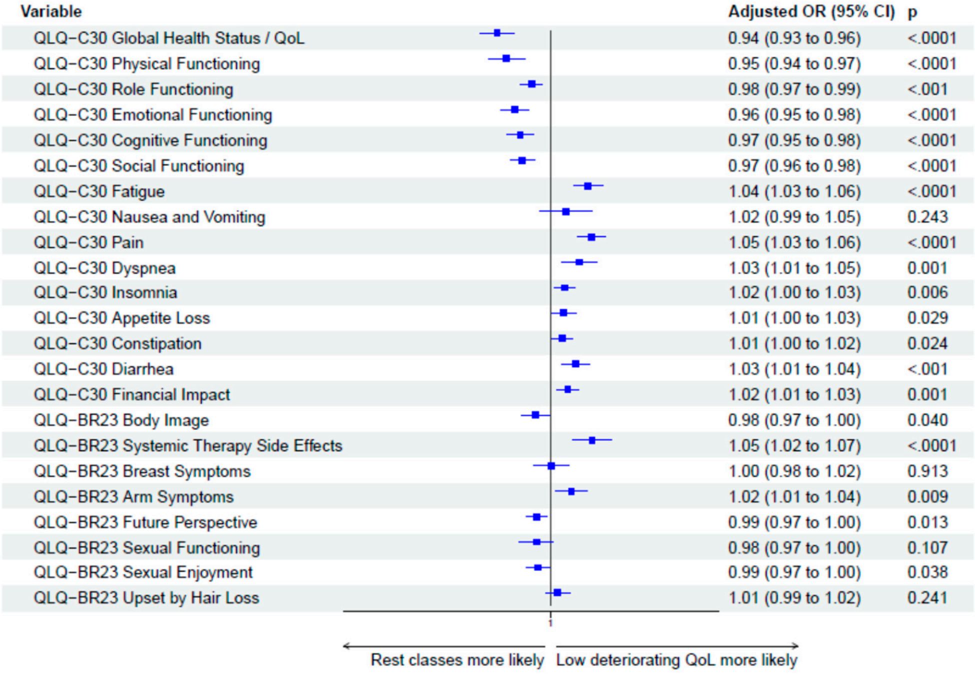

Figure S2. Odds ratios (95% CI), adjusted for clinical site, for psychological scales associated with Low deteriorating QoL trajectory class versus other classes at baseline. Values >1 indicate higher likelihood of Low deteriorating QoL; values <1 indicate higher likelihood of other classes.

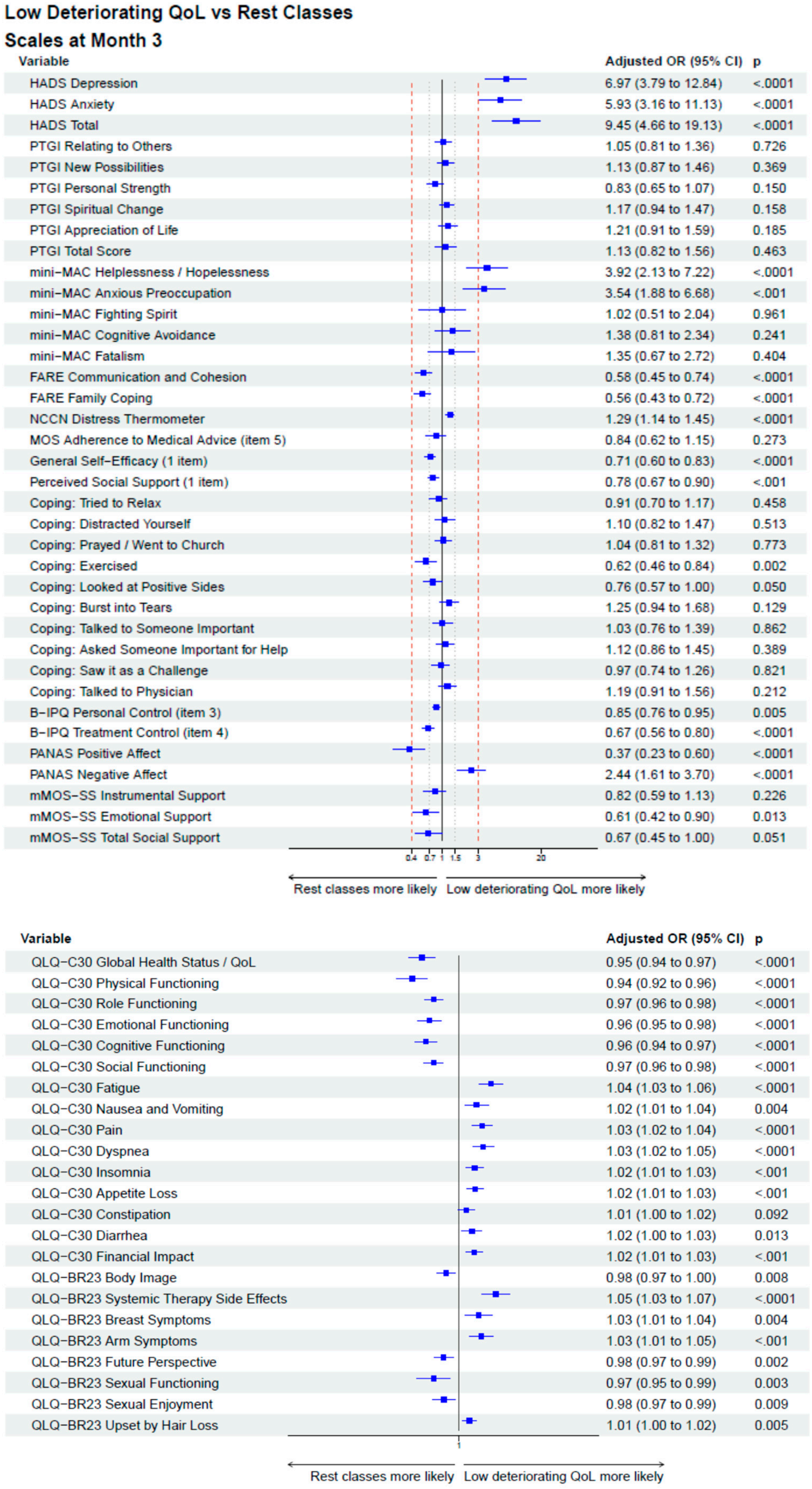

Figure S3. Odds ratios (95% CI), adjusted for clinical site, for psychological scales associated with Low deteriorating QoL trajectory class versus other classes at month 3. Values >1 indicate higher likelihood of Low deteriorating QoL; values <1 indicate higher likelihood of other classes.

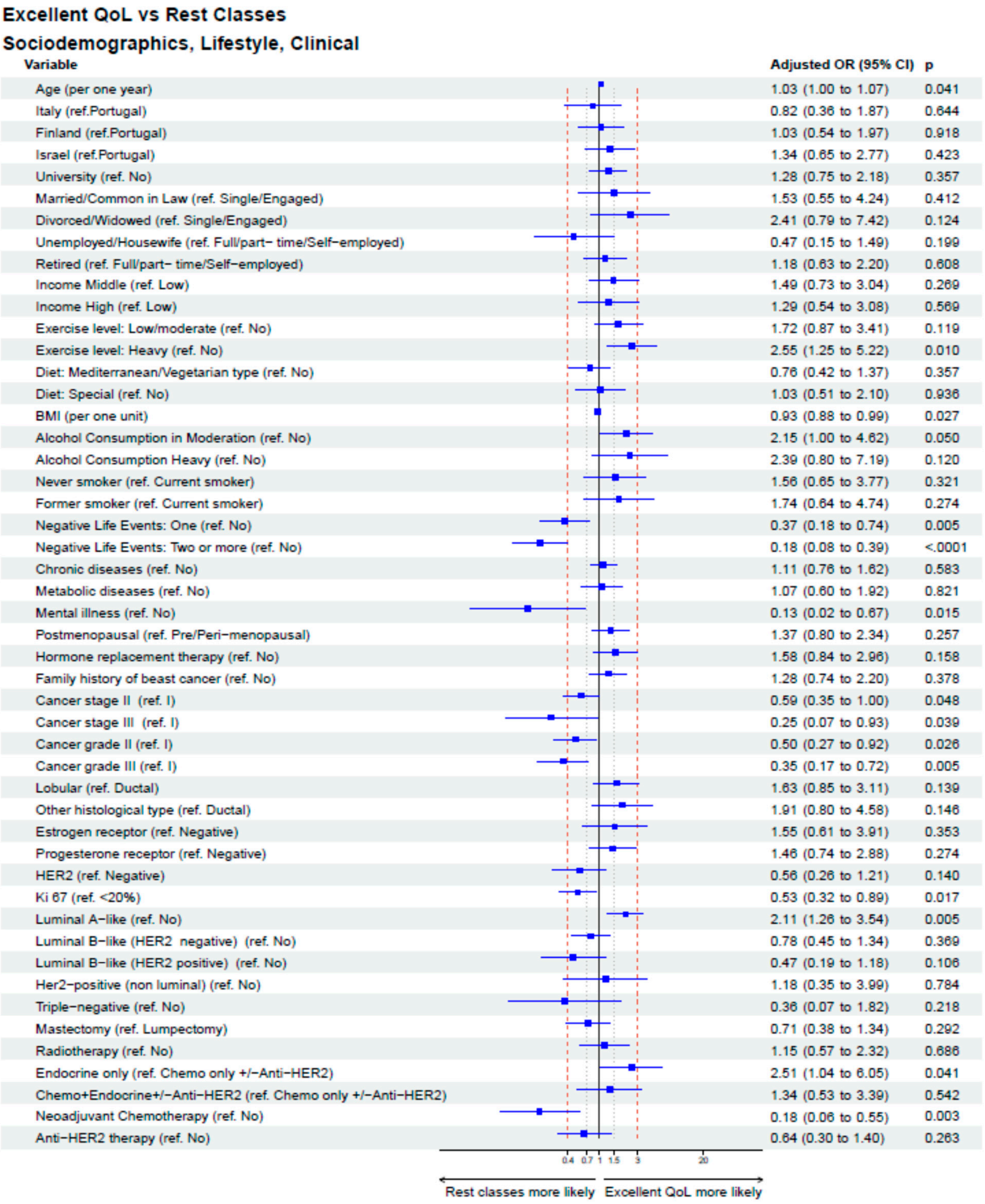

Figure S4. Odds ratios (95% CI), adjusted for clinical site, for sociodemographic, lifestyle, clinical and cancer-related factors associated with the Excellent QoL trajectory class versus other classes at baseline. Values >1 indicate higher likelihood of Excellent QoL; values <1 indicate higher likelihood of other classes.

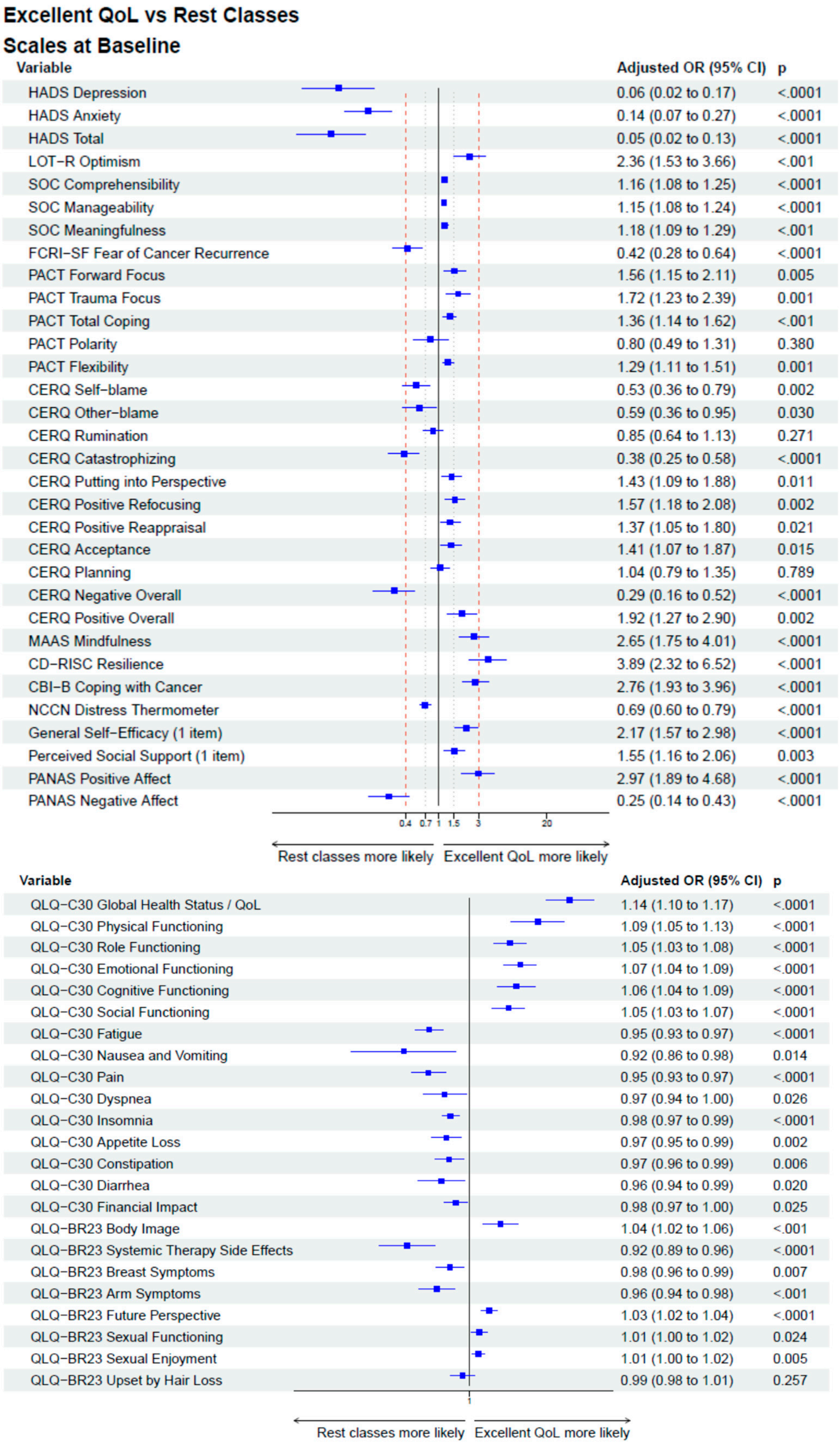

Figure S5. Odds ratios (95% CI), adjusted for clinical site, for psychological scales associated with Excellent QoL trajectory class versus other classes at baseline. Values >1 indicate higher likelihood of Excellent QoL; values <1 indicate higher likelihood of other classes.

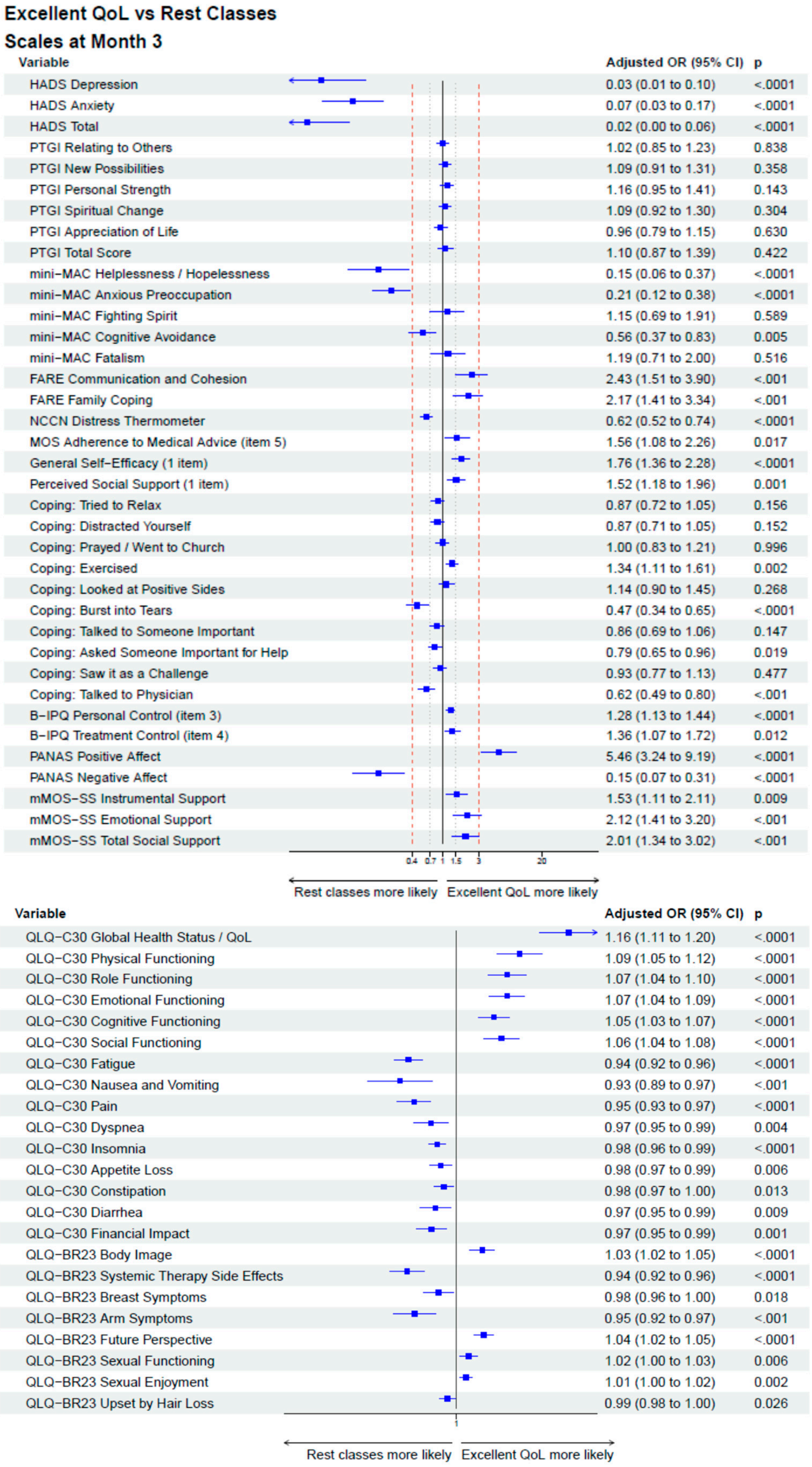

Figure S6. Odds ratios (95% CI), adjusted for clinical site, for psychological scales associated with Excellent QoL trajectory class versus other classes at month 3. Values >1 indicate higher likelihood of Excellent QoL; values <1 indicate higher likelihood of other classes.

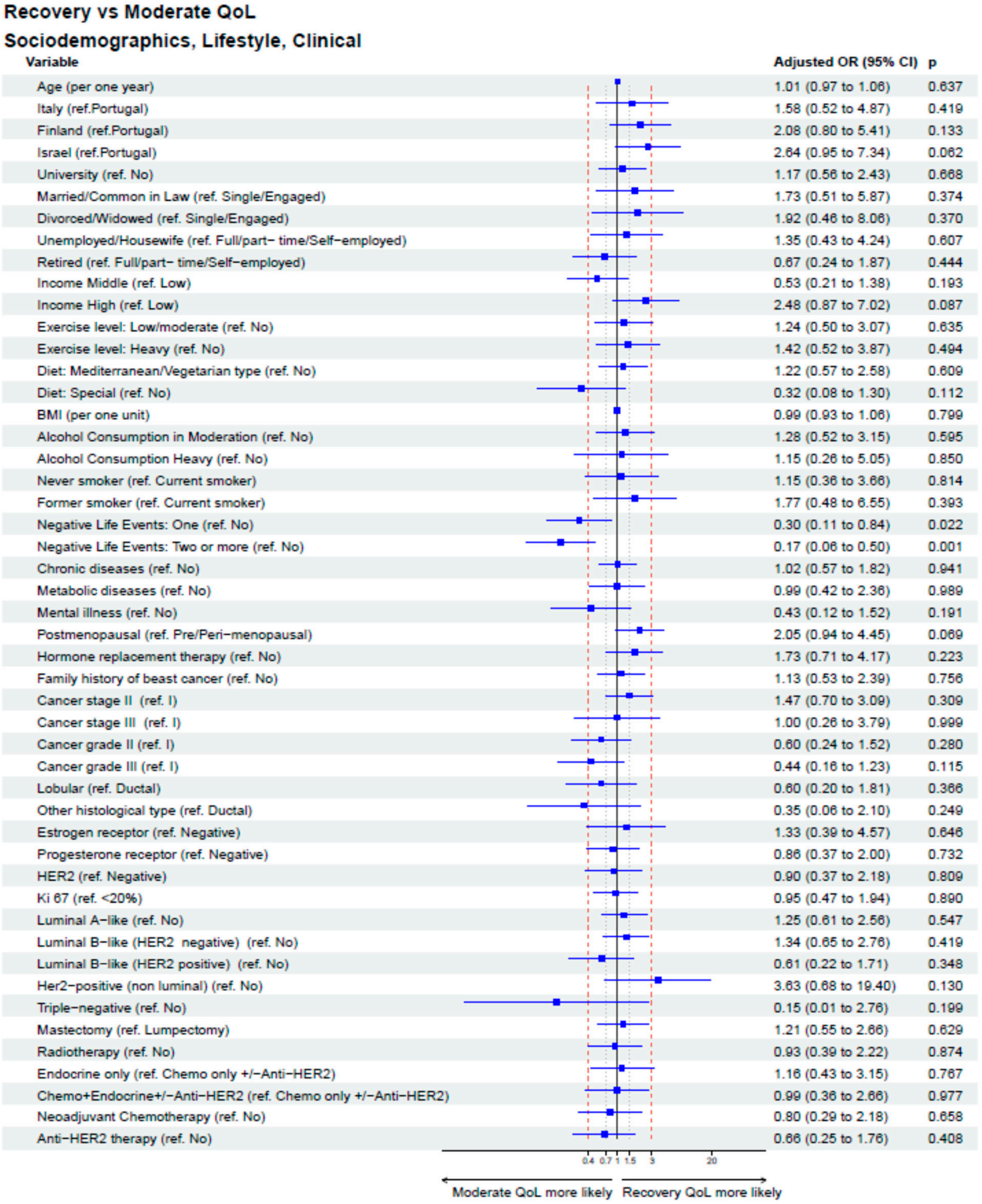

Figure S7. Odds ratios (95% CI), adjusted for clinical site, for sociodemographic, lifestyle, clinical and cancer-related factors associated with the Recovering QoL trajectory class versus Moderate QoL class at baseline. Values >1 indicate higher likelihood of Recovering QoL; values <1 indicate higher likelihood of Moderate QoL.

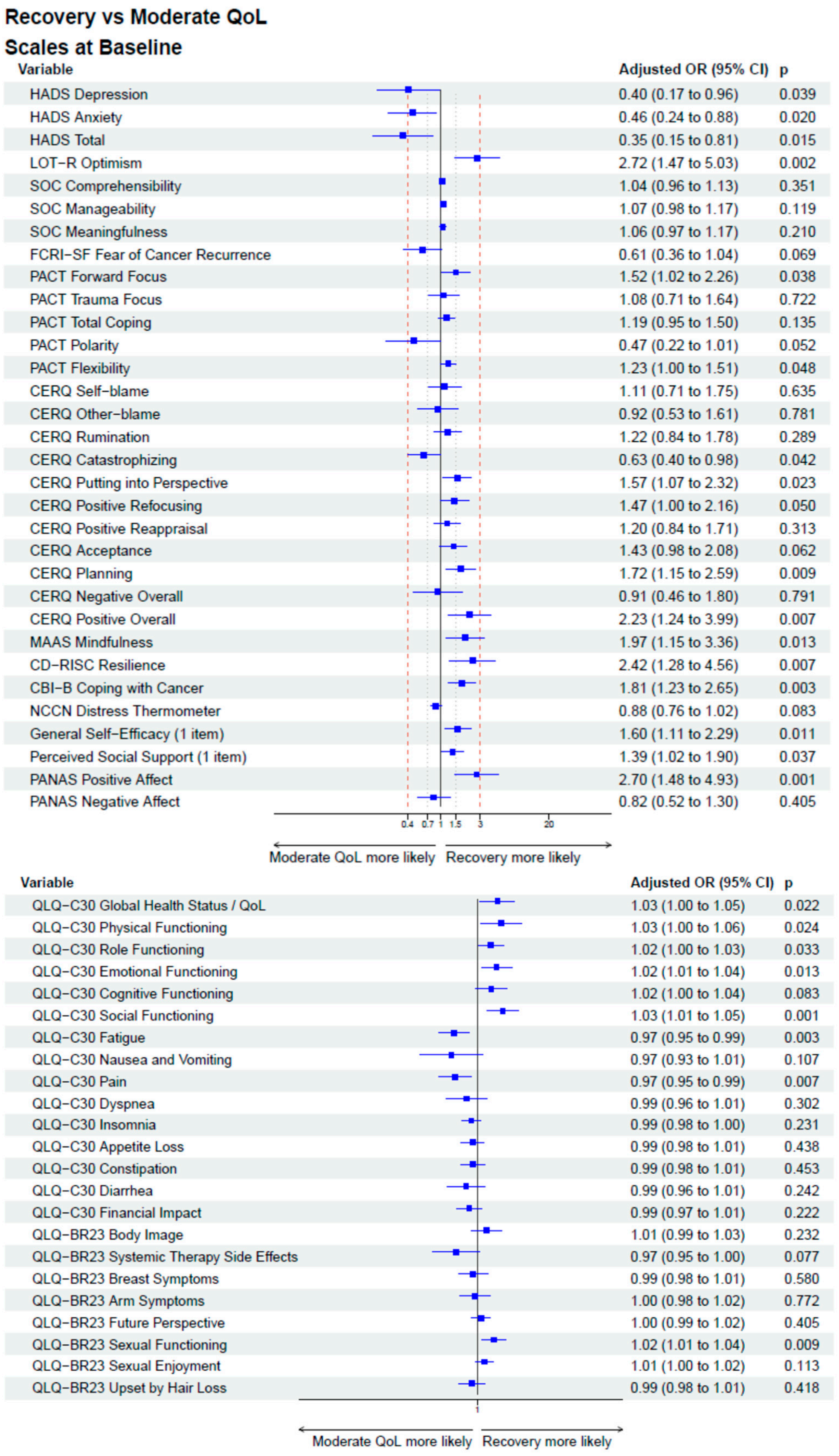

Figure S8. Odds ratios (95% CI), adjusted for clinical site, for psychological scales associated with the Recovering QoL trajectory class versus Moderate QoL class at baseline. Values >1 indicate higher likelihood of Recovery; values <1 indicate higher likelihood of Moderate QoL.

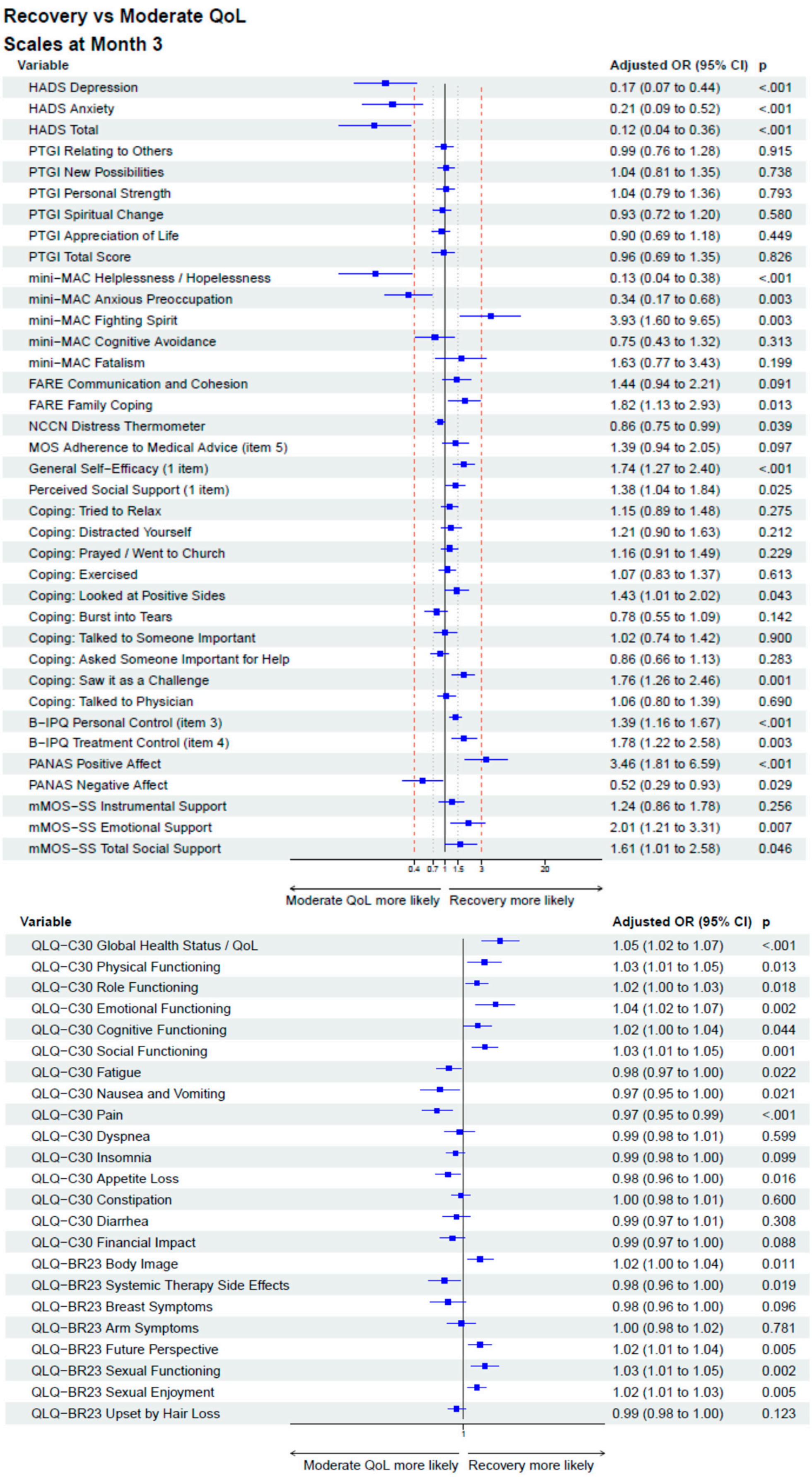

Figure S9. Odds ratios (95% CI), adjusted for clinical site, for psychological scales associated with the Recovering QoL trajectory class versus Moderate QoL class at month 3. at baseline. Values >1 indicate higher likelihood of Recovery; values <1 indicate higher likelihood of Moderate QoL.

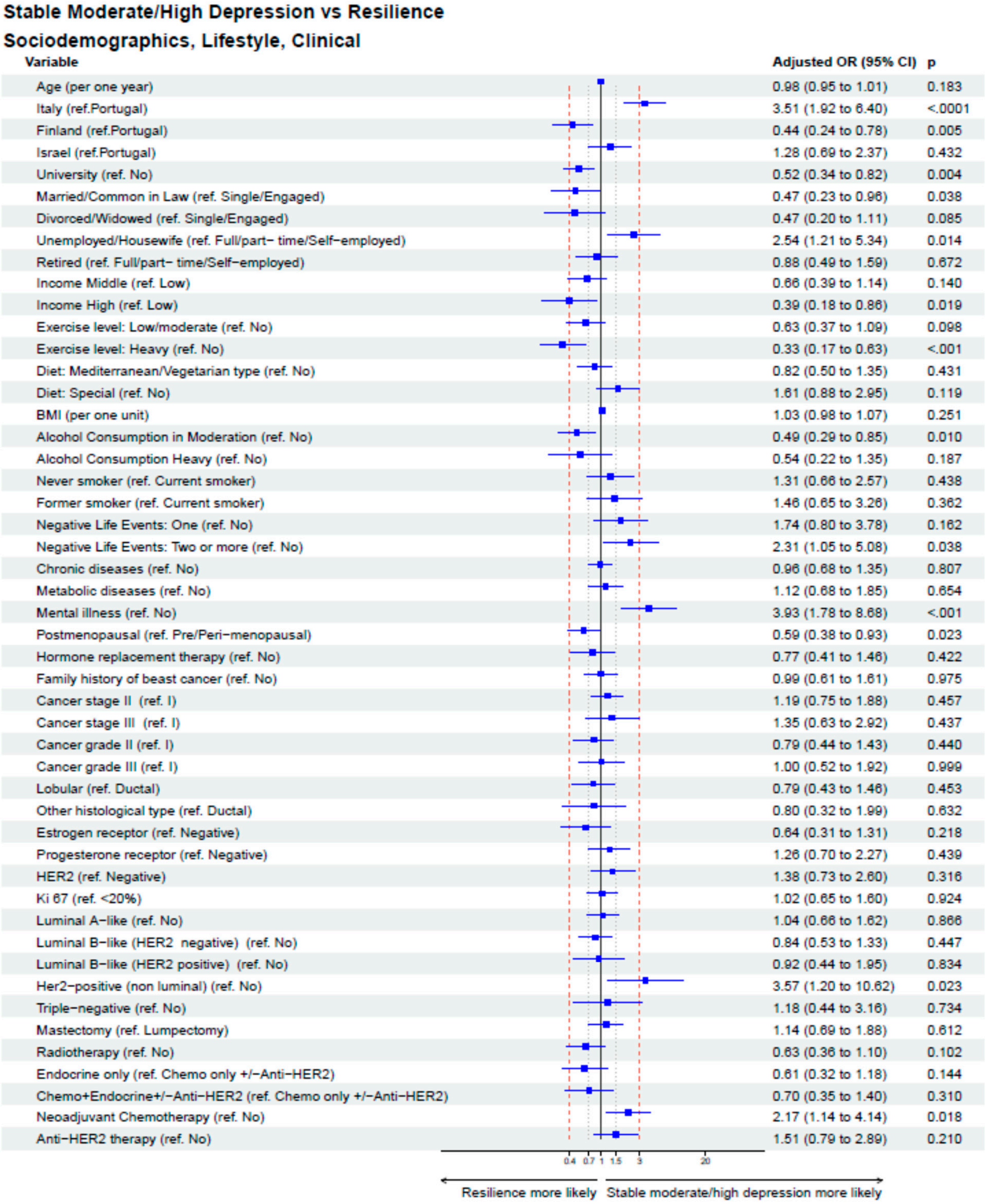

Figure S10. Odds ratios (95% CI), adjusted for clinical site, for sociodemographic, lifestyle, clinical and cancer-related factors associated with the Stable Moderate/High Depression class versus the Resilient Depression trajectory class at baseline. Values >1 indicate higher likelihood of Stable Moderate/High Depression; values <1 indicate higher likelihood of Resilient class.

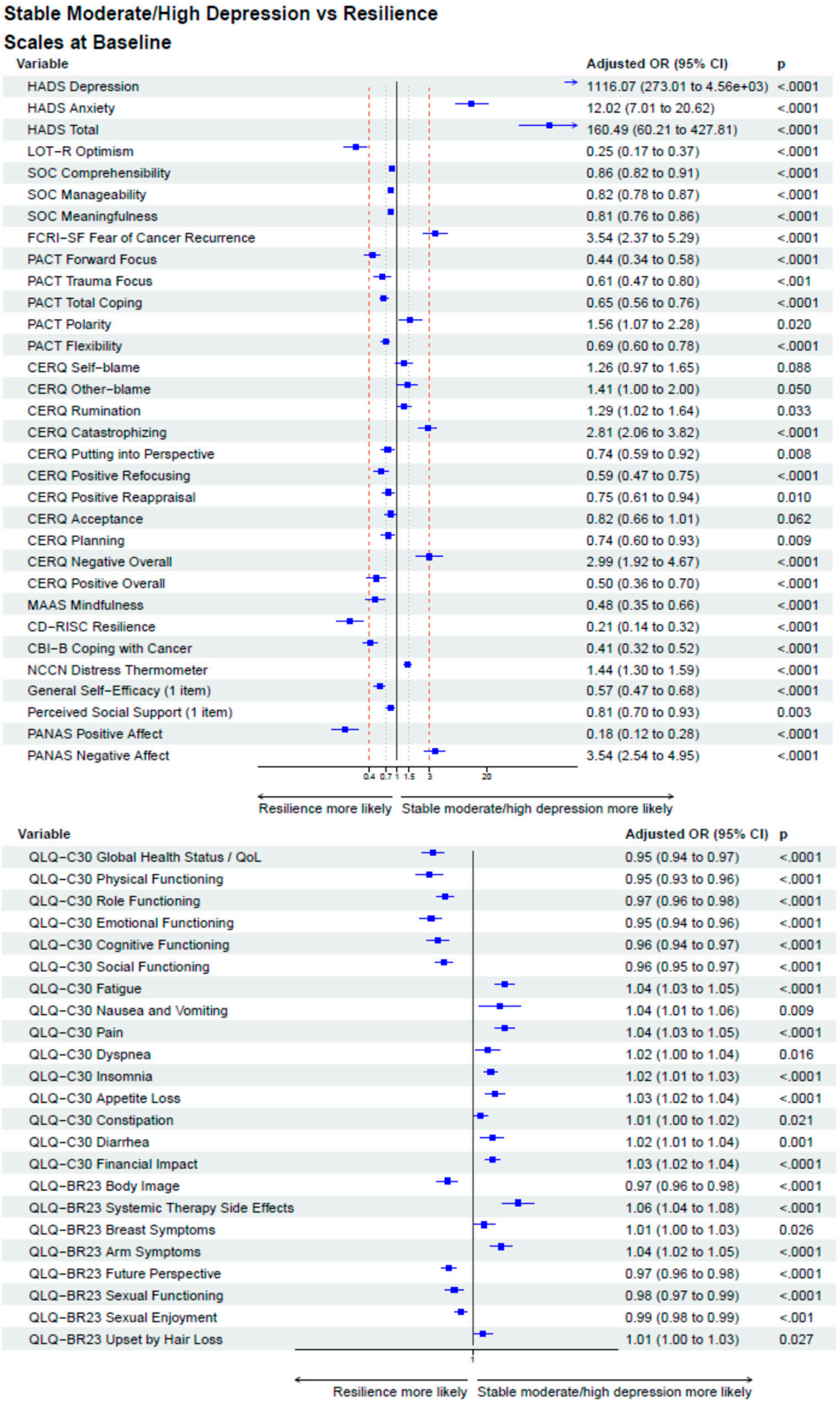

Figure S11. Odds ratios (95% CI), adjusted for clinical site, for psychological scales associated with the Stable Moderate/High Depression class versus the Resilient Depression trajectory class at baseline. Values >1 indicate higher likelihood of Stable Moderate/High Depression; values <1 indicate higher likelihood of Resilient class.

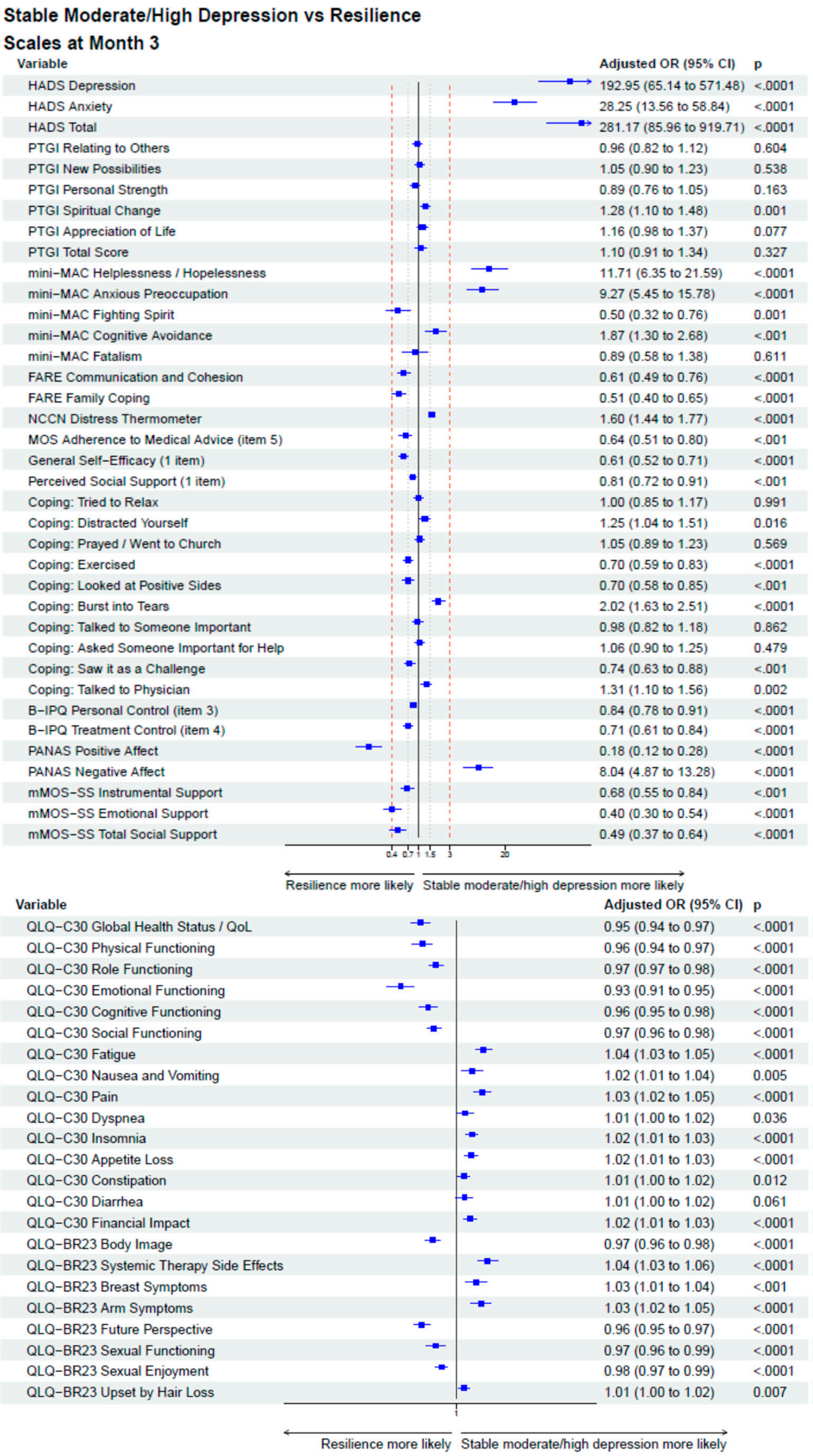

Figure S12. Odds ratios (95% CI), adjusted for clinical site, for psychological scales associated with the Stable Moderate/High Depression class versus the Resilient Depression trajectory class at month 3. Values >1 indicate higher likelihood of Stable Moderate/High Depression; values <1 indicate higher likelihood of Resilient class.

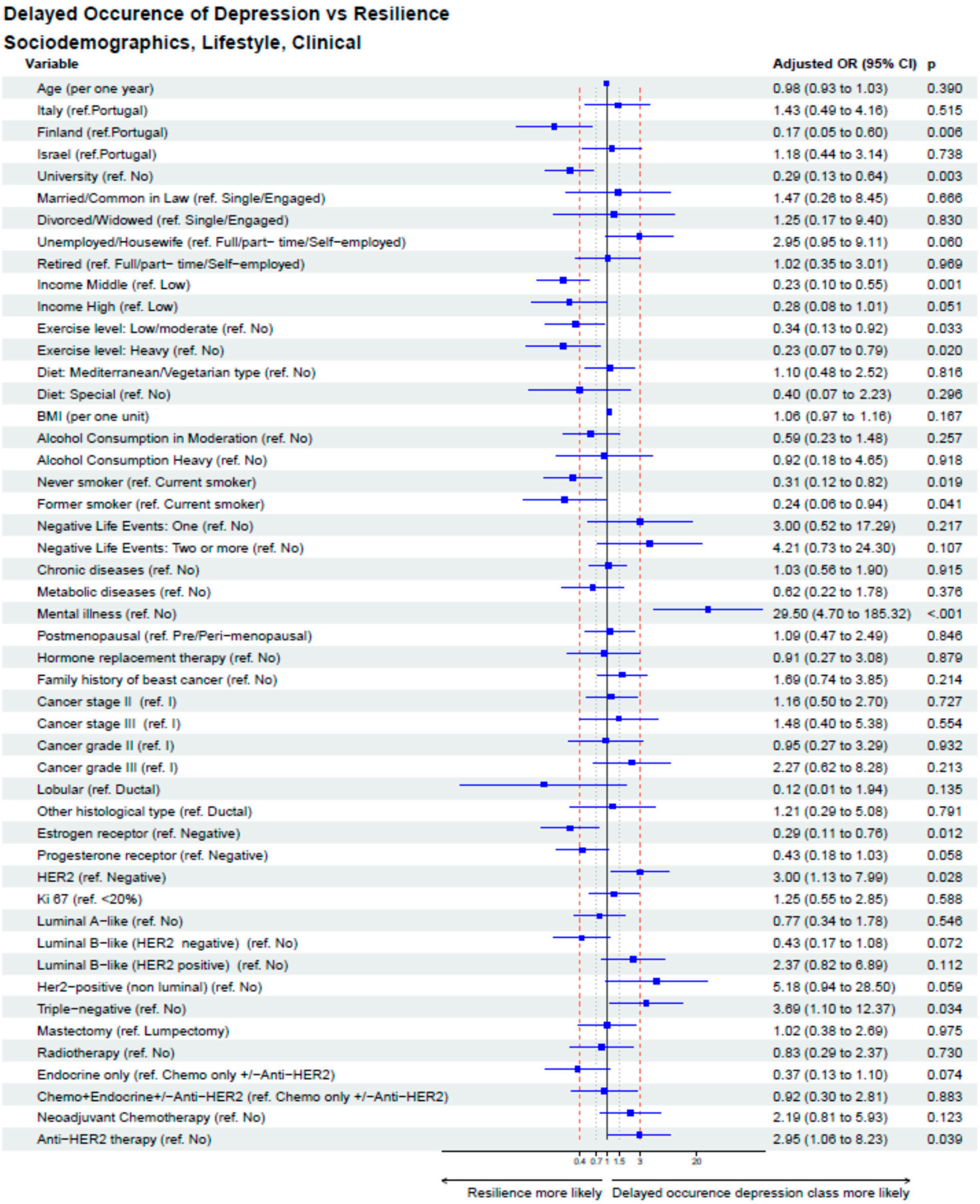

Figure S13. Odds ratios (95% CI), adjusted for clinical site, for sociodemographic, lifestyle, clinical and cancer-related factors associated with the Late Occurrence Depression class versus the Resilient Depression class at baseline. Values >1 indicate higher likelihood of Late Occurrence; values <1 indicate higher likelihood of Resilient class.

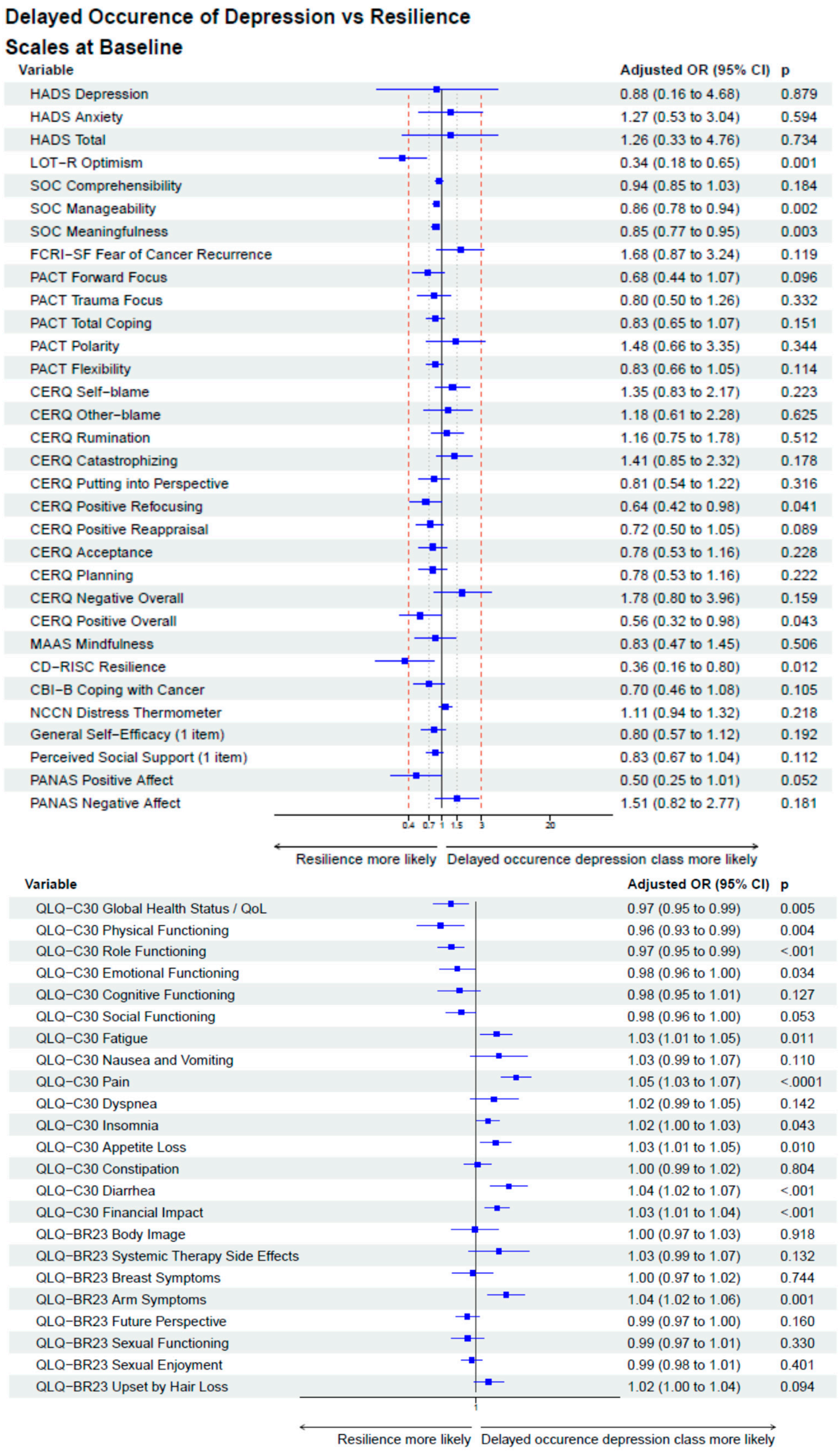

Figure S14. Odds ratios (95% CI), adjusted for clinical site, for psychological scales associated with the Late Occurrence Depression class versus the Resilient Depression class at baseline. Values >1 indicate higher likelihood of Late Occurrence; values <1 indicate higher likelihood of Resilient class.

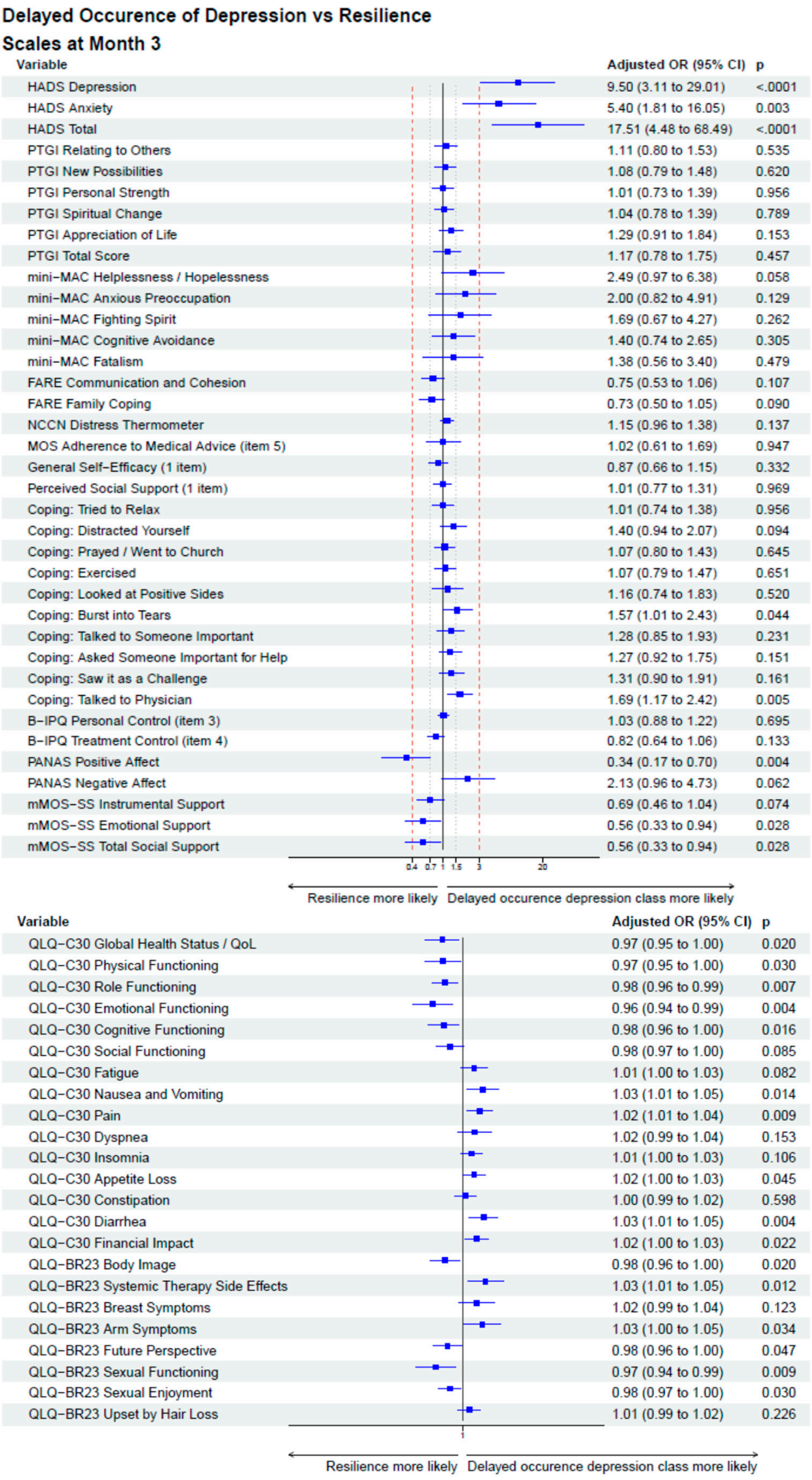

Figure S15. Odds ratios (95% CI), adjusted for clinical site, for psychological scales associated with the Late Occurrence Depression class versus the Resilient Depression class at month 3. Values >1 indicate higher likelihood of Late Occurrence; values <1 indicate higher likelihood of Resilient class.

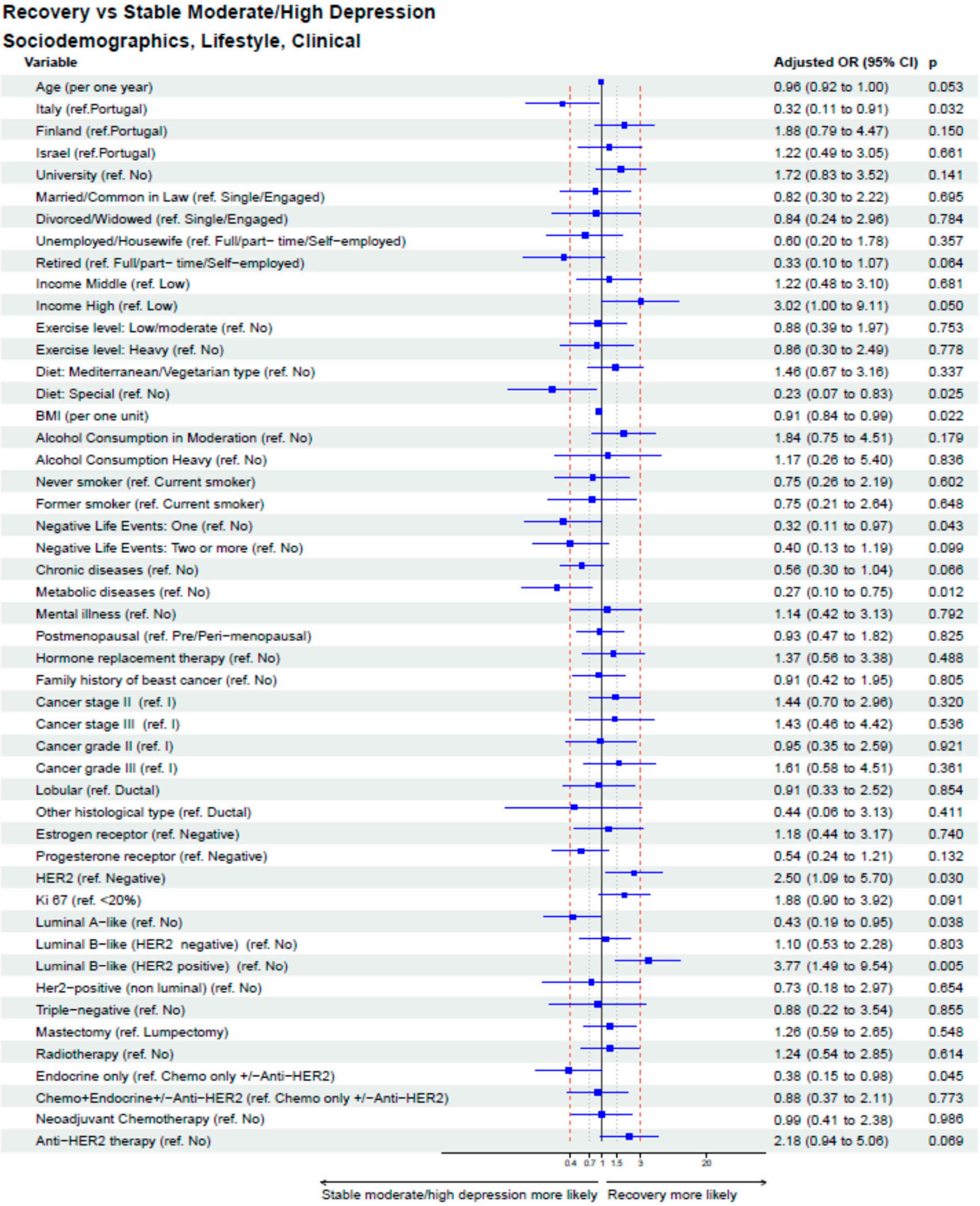

Figure S16. Odds ratios (95% CI), adjusted for clinical site, for sociodemographic, lifestyle, clinical and cancer-related factors associated with the Recovering Depression class versus the Stable Moderate/High Depression class at baseline. Values >1 indicate higher likelihood of Recovery; values <1 indicate higher likelihood of Stable Moderate/High Depression.

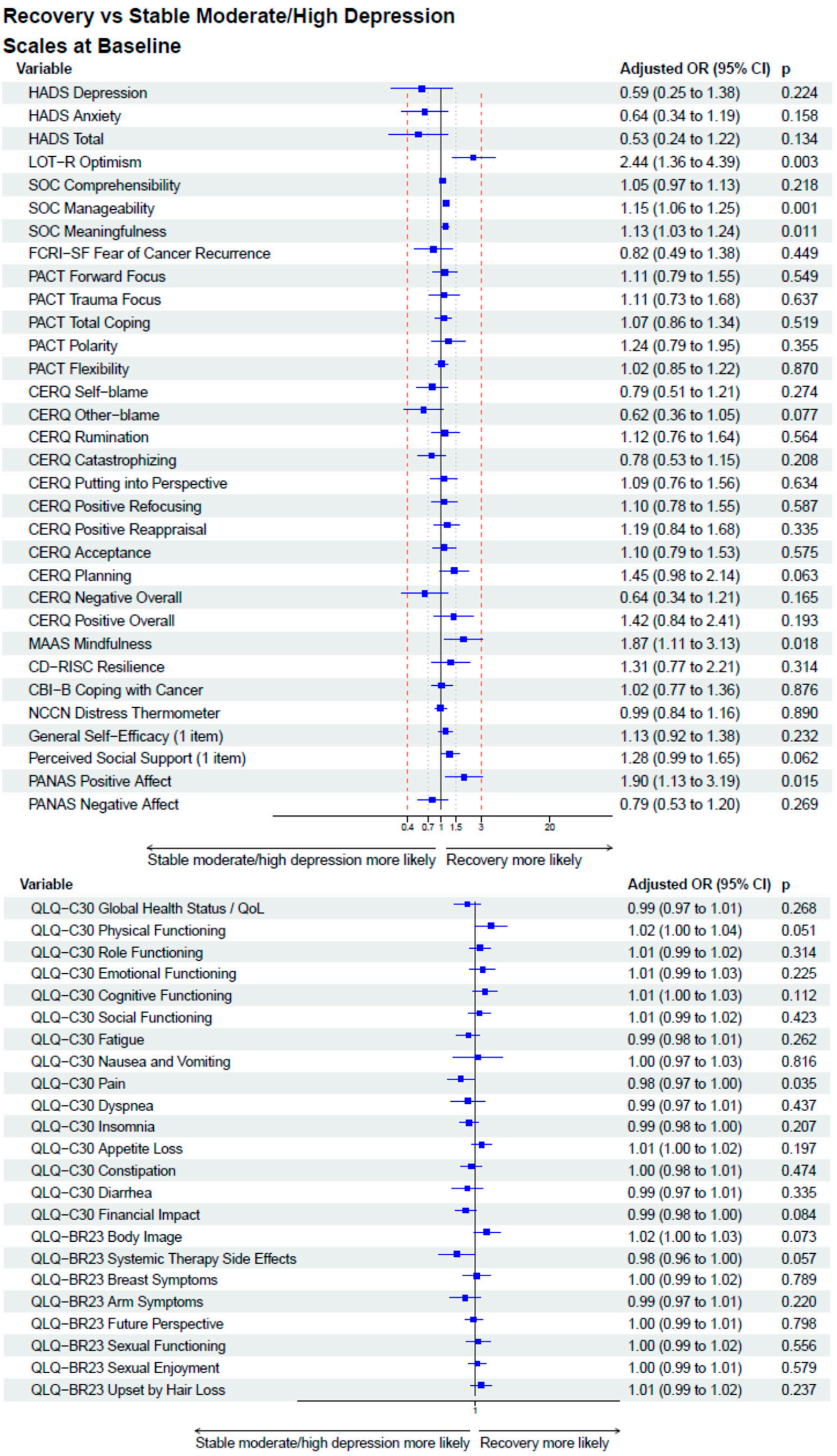

Figure S17. Odds ratios (95% CI), adjusted for clinical site, for psychological scales associated with the Recovering Depression class versus the Stable Moderate/High Depression class at baseline. Values >1 indicate higher likelihood of Recovery; values <1 indicate higher likelihood of Stable Moderate/High Depression.

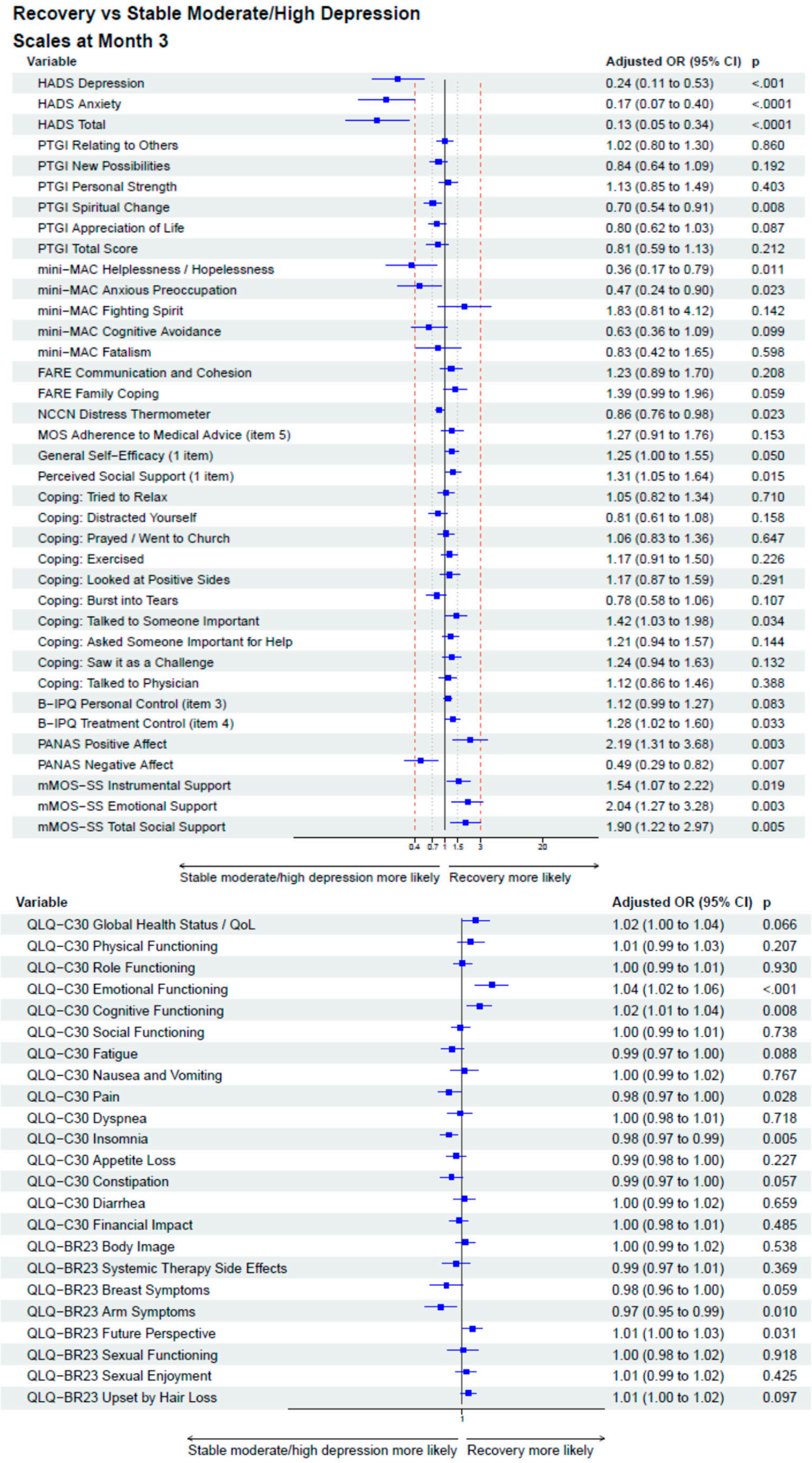

Figure S18. Odds ratios (95% CI), adjusted for clinical site, for psychological scales associated with the Recovering Depression class versus the Stable Moderate/High Depression class at month 3. Values >1 indicate higher likelihood of Recovery; values <1 indicate higher likelihood of Stable Moderate/High Depression.
