# Supplementary figures and images for: In Silico Psycho-Oncology: Understanding Resilience Pathways in Breast Cancer—Determinants of Longitudinal Depression and Quality-of-Life Trajectories
Source: J Pers Med. 2026 Apr 7;16(4):209. doi: 10.3390/jpm16040209 (PMC13117978; doi:10.3390/jpm16040209)

## Correlation Matrix at Baseline

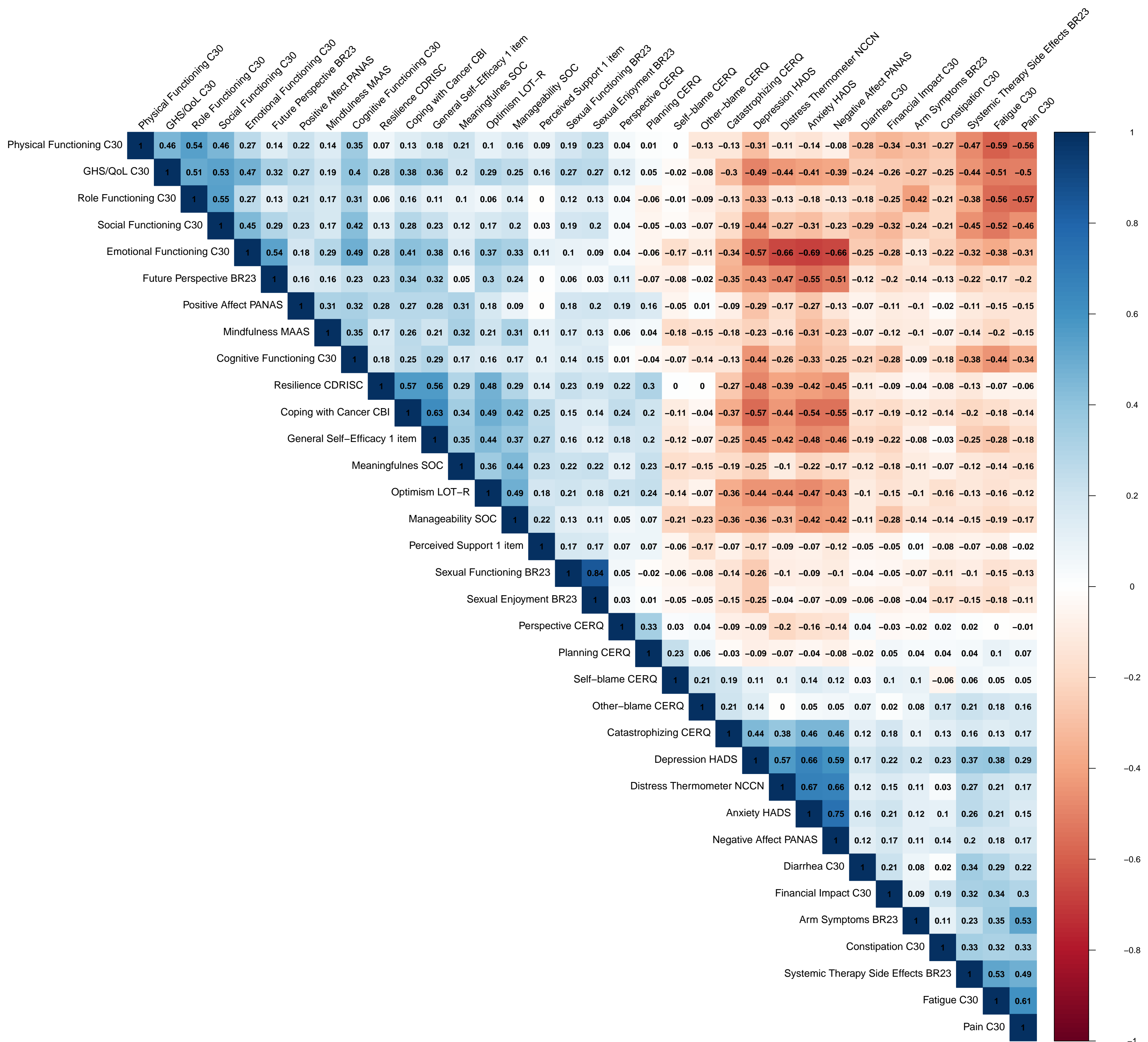

## Correlation Matrix at Month 3

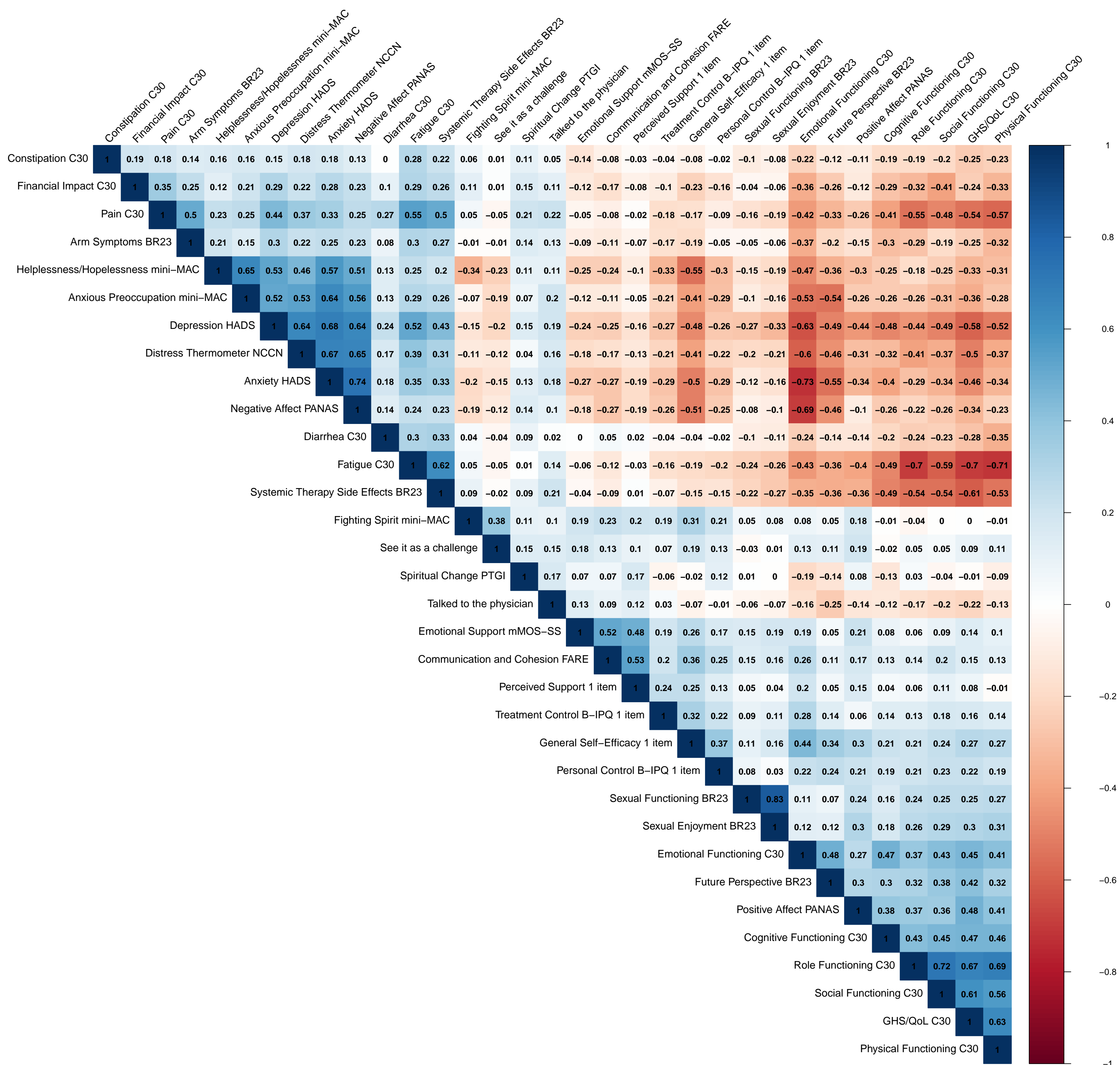

Supplement: Supplementary file 1 [file jpm-16-00209-s001.zip › Supplementary Material S2 Correlation matrices among predictors selected at baseline and month 3.pdf]
